# Expression of dehydroshikimate dehydratase in poplar induces transcriptional and metabolic changes in the phenylpropanoid pathway

**DOI:** 10.1093/jxb/erae251

**Published:** 2024-05-29

**Authors:** Emine Akyuz Turumtay, Halbay Turumtay, Yang Tian, Chien-Yuan Lin, Yen Ning Chai, Katherine B Louie, Yan Chen, Anna Lipzen, Thomas Harwood, Kavitha Satish Kumar, Benjamin P Bowen, Qian Wang, Shawn D Mansfield, Matthew J Blow, Christopher J Petzold, Trent R Northen, Jenny C Mortimer, Henrik V Scheller, Aymerick Eudes

**Affiliations:** Feedstocks Division, Joint BioEnergy Institute, Emeryville, CA, USA; Environmental Genomics and Systems Biology Division, Lawrence Berkeley National Laboratory, Berkeley, CA, USA; Recep Tayyip Erdogan University, Department of Chemistry, 53100, Rize, Turkiye; Feedstocks Division, Joint BioEnergy Institute, Emeryville, CA, USA; Environmental Genomics and Systems Biology Division, Lawrence Berkeley National Laboratory, Berkeley, CA, USA; Karadeniz Technical University, Department of Energy System Engineering, 61830, Trabzon, Turkiye; Feedstocks Division, Joint BioEnergy Institute, Emeryville, CA, USA; Environmental Genomics and Systems Biology Division, Lawrence Berkeley National Laboratory, Berkeley, CA, USA; Feedstocks Division, Joint BioEnergy Institute, Emeryville, CA, USA; Environmental Genomics and Systems Biology Division, Lawrence Berkeley National Laboratory, Berkeley, CA, USA; Feedstocks Division, Joint BioEnergy Institute, Emeryville, CA, USA; Environmental Genomics and Systems Biology Division, Lawrence Berkeley National Laboratory, Berkeley, CA, USA; Environmental Genomics and Systems Biology Division, Lawrence Berkeley National Laboratory, Berkeley, CA, USA; Joint Genome Institute, Lawrence Berkeley National Laboratory, Berkeley, CA, USA; Feedstocks Division, Joint BioEnergy Institute, Emeryville, CA, USA; Biological Systems & Engineering Division, Lawrence Berkeley National Laboratory, Berkeley, CA, USA; Joint Genome Institute, Lawrence Berkeley National Laboratory, Berkeley, CA, USA; Environmental Genomics and Systems Biology Division, Lawrence Berkeley National Laboratory, Berkeley, CA, USA; Joint Genome Institute, Lawrence Berkeley National Laboratory, Berkeley, CA, USA; Feedstocks Division, Joint BioEnergy Institute, Emeryville, CA, USA; Environmental Genomics and Systems Biology Division, Lawrence Berkeley National Laboratory, Berkeley, CA, USA; Environmental Genomics and Systems Biology Division, Lawrence Berkeley National Laboratory, Berkeley, CA, USA; Joint Genome Institute, Lawrence Berkeley National Laboratory, Berkeley, CA, USA; Department of Wood Science, University of British Columbia, Vancouver, BC, Canada; Department of Botany, University of British Columbia, Vancouver, BC, Canada; Department of Wood Science, University of British Columbia, Vancouver, BC, Canada; Department of Botany, University of British Columbia, Vancouver, BC, Canada; DOE Great Lakes Bioenergy Research Center, Wisconsin Energy Institute, Madison, WI 53726, USA; Joint Genome Institute, Lawrence Berkeley National Laboratory, Berkeley, CA, USA; Feedstocks Division, Joint BioEnergy Institute, Emeryville, CA, USA; Biological Systems & Engineering Division, Lawrence Berkeley National Laboratory, Berkeley, CA, USA; Environmental Genomics and Systems Biology Division, Lawrence Berkeley National Laboratory, Berkeley, CA, USA; Joint Genome Institute, Lawrence Berkeley National Laboratory, Berkeley, CA, USA; Feedstocks Division, Joint BioEnergy Institute, Emeryville, CA, USA; Environmental Genomics and Systems Biology Division, Lawrence Berkeley National Laboratory, Berkeley, CA, USA; School of Agriculture, Food and Wine & Waite Research Institute, University of Adelaide, Glen Osmond, SA, Australia; Feedstocks Division, Joint BioEnergy Institute, Emeryville, CA, USA; Environmental Genomics and Systems Biology Division, Lawrence Berkeley National Laboratory, Berkeley, CA, USA; Department of Plant and Microbial Biology, University of California, Berkeley, Berkeley, CA, USA; Feedstocks Division, Joint BioEnergy Institute, Emeryville, CA, USA; Environmental Genomics and Systems Biology Division, Lawrence Berkeley National Laboratory, Berkeley, CA, USA; Shenzhen Institute of Advanced Technology, China

**Keywords:** Aromatics, bioenergy, cell wall, lignin, metabolomics, *Populus*, RNA-seq, systems biology

## Abstract

Modification of lignin in feedstocks via genetic engineering aims to reduce biomass recalcitrance to facilitate efficient conversion processes. These improvements can be achieved by expressing exogenous enzymes that interfere with native biosynthetic pathways responsible for the production of the lignin precursors. *In planta* expression of a bacterial 3-dehydroshikimate dehydratase in poplar trees reduced lignin content and altered the monomer composition, which enabled higher yields of sugars after cell wall polysaccharide hydrolysis. Understanding how plants respond to such genetic modifications at the transcriptional and metabolic levels is needed to facilitate further improvement and field deployment. In this work, we acquired fundamental knowledge on lignin-modified poplar expressing 3-dehydroshikimate dehydratase using RNA-seq and metabolomics. The data clearly demonstrate that changes in gene expression and metabolite abundance can occur in a strict spatiotemporal fashion, revealing tissue-specific responses in the xylem, phloem, or periderm. In the poplar line that exhibited the strongest reduction in lignin, we found that 3% of the transcripts had altered expression levels and ~19% of the detected metabolites had differential abundance in the xylem from older stems. The changes affected predominantly the shikimate and phenylpropanoid pathways as well as secondary cell wall metabolism, and resulted in significant accumulation of hydroxybenzoates derived from protocatechuate and salicylate.

## Teaser

Poplar modified with an exogenous 3-dehydroshikimate dehydratase displays transcriptional and metabolic changes in the lignin biosynthetic pathway and overproduces large quantities of hydroxybenzoate derivatives.

## Introduction

Lignocellulosic biomass represents an important renewable source of sugars and aromatics for the production of fuels and specialty chemicals. These sugars and aromatics are contained in polymers that constitute plant cell walls: cellulose is a polysaccharide made of glucose, while hemicelluloses are largely made of mixed sugars but are often dominated by xylose. In contrast, lignin is formed via the oxidative polymerization of 4-hydroxycinnamyl alcohols (or monolignols) that derive from the phenylpropanoid pathway. These polymers are particularly abundant in tissues that develop thick secondary cell walls, and lignin deposited in these tissues confers cohesiveness, mechanical strength, and hydrophobicity. A better understanding of the biological mechanisms that govern cell wall synthesis and regulation is needed to optimize plant biomass properties for efficient and sustainable conversion into fuels, biopolymers, and specialty chemicals (de Vries *et al*., 2021b).

Woody perennials are expected to represent a significant share of the dedicated biomass grown for bioenergy (Langholtz *et al.*, 2016). Short-rotation woody crops, such as fast growing *Populus*, *Salix*, and *Eucalyptus* species produce large amounts of lignocellulose and can typically be harvested year-round and over several years, which ensures a stable and predictable supply of bioenergy feedstocks (Hinchee *et al.*, 2009; Mizrachi *et al.*, 2012). In particular, several species in the genus *Populus* display rapid growth and high productivity, do not require a large amount of inputs for cultivation, and can be grown on marginal lands to restore soil fertility (An *et al.*, 2021). The availability of the genome sequence of *Populus trichocarpa* (black cottonwood) has facilitated the genetic analysis and improvement of several *Populus* species (Tuskan *et al.*, 2006).

The efficient release of monosaccharides and simple aromatics from cell walls is an essential step for biological conversion of biomass into advanced fuels and chemicals via industrial fermentation. However, lignin impedes this process by interacting with polysaccharides and thereby protecting them from enzymatic deconstruction. Lignin itself is not easily deconstructed into simple aromatics due to its inherent complexity, but also due to molecular rearrangements occurring within the polymer during biomass pretreatment processes. These challenges have prompted the plant research community to genetically engineer plant cell walls to facilitate biomass utilization. In particular, several lignin bioengineering approaches have been designed to reduce cell wall recalcitrance in crops (Mahon and Mansfield, 2019; Liu and Eudes, 2022). One classic approach is the generation of plant lines affected in one or several genes involved in a specific step of the lignin biosynthetic pathway, resulting in plants with reduced lignin and enhanced biomass saccharification efficiency (de Vries *et al*., 2021a; Sulis *et al.*, 2023). Our group has shown that heterologous expression of bacterial 3-dehydroshikimate dehydratase (QsuB) targeted to plastids is an effective approach to reroute the shikimate pool away from lignin biosynthesis, resulting in plants that accumulate the simple aromatic protocatechuate (3,4-dihydroxybenzoic acid, DHBA) at the expense of lignin (Eudes *et al.*, 2015). This strategy was translated to poplar to design lines with reduced lignin and higher DHBA content in biomass (Unda *et al.*, 2022). Engineered QsuB poplar lines show reduced recalcitrance to enzymatic saccharification, release higher amounts of simple aromatics, and enable higher titres of biofuels and bioproducts after deconstruction and microbial fermentation (C.-Y. Lin *et al.*, 2022; Umana *et al.*, 2022).

Systems biology approaches have been used to gain a better understanding of various physiological processes in plants, and technologies such as transcriptomics and untargeted metabolomics are commonly used to generate the underlying data. For example, omics data have been generated in trees to study genotype discrimination (Robinson *et al*., 2005), nitrogen nutrition (Chen *et al.*, 2021b), seed germination (Qu *et al.*, 2019), vascular development (Chen *et al.*, 2021a), wood formation (Robinson *et al*., 2007, 2022; Sundell *et al.*, 2017), and responses to stresses such as drought (Barchet *et al*., 2014; Hamanishi *et al.*, 2015), heat (Zhao *et al.*, 2022), autumn cold (Dauwe *et al.*, 2012), pathogens (Zhang *et al.*, 2023), and ultraviolet light (Kaling *et al.*, 2015), as well as regenerative capacity of somatic embryos (Robinson *et al*., 2009). This approach is also useful to investigate global transcriptional and metabolomic changes occurring in genetically modified *Populus* (Xue *et al.*, 2013; Robinson *et al*., 2018; Wang *et al.*, 2018; Zhang *et al.*, 2019; Hu *et al.*, 2022).

**Fig. 1.**
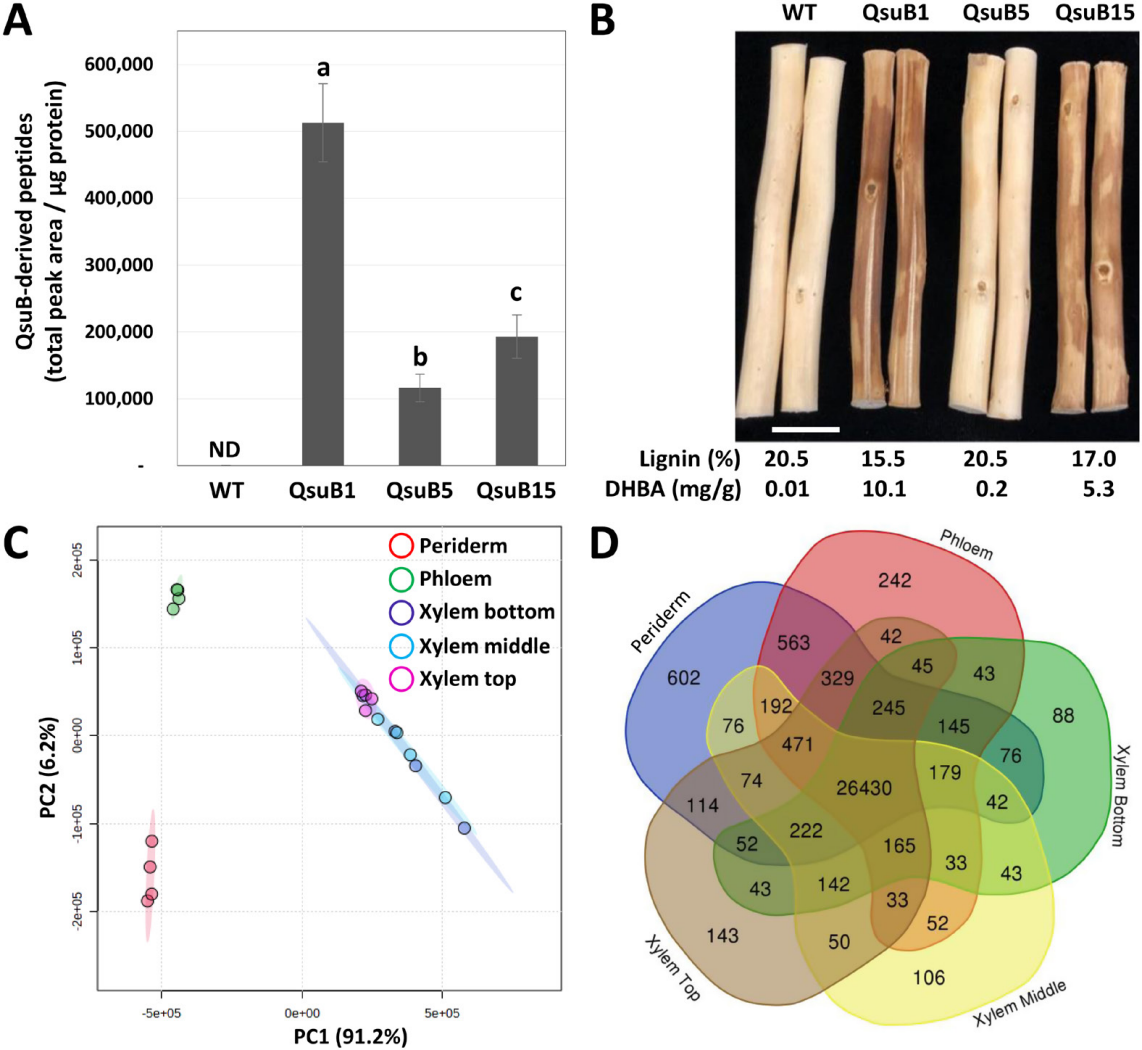
3-Dehydroshikimate dehydratase (QsuB) poplar lines used in this study and analysis of transcripts isolated from stems of wild type (WT). (A) QsuB protein abundance in the xylem from intermediate stems of QsuB poplar lines. Values are means and SD of four biological replicates. Means with different letters represent statistically significant differences using Tukey’s pairwise comparison (*P*<0.05). ND, not detected. (B) Average lignin and protocatechuate (3,4-dihydroxybenzoic acid, DHBA) contents in stems from WT and QsuB1, QsuB5, and QsuB15 transgenic lines. Debarked stems are shown. Scale bar is 1 cm. Values are from C-Y. Lin *et al.* (2022). (C, D) Principal component analysis plot (C) and Venn diagram (D) of the 31 082 unique transcripts identified in stem tissues from WT.

In this study, considering the important role of the shikimate and phenylpropanoid pathways in plant secondary metabolism (Maeda and Dudareva, 2012; Dong and Lin, 2021), we investigated the metabolic and transcriptional changes that occur in stems of transgenic QsuB poplar. In order to gain a comprehensive overview of these changes we analysed separately the xylem, phloem, and periderm tissues. The xylem consists of fibre cells and vessels with lignified thickened secondary cell walls that participate in the transport of water and provide mechanical strength for vertical growth; the phloem is composed of sieve elements for the transport of nutrients as well as fibres; and the periderm is rich in suberized cells and has protective functions. Moreover, the xylem tissue was collected from three different parts of the stem (i.e. older, intermediate, and younger) to obtain three distinct stages of growth and secondary cell wall formation. In addition to the wild-type control, three QsuB transgenic lines with varying levels of QsuB protein abundance, lignin reduction, and DHBA accumulation were analysed (Fig. 1A, B).

## Materials and methods

### Plant material

Hybrid poplar (*Populus alba × grandidentata*; *P39*) wild type (WT) and QsuB transgenic lines were previously described (Unda *et al.*, 2022). WT and lines QsuB1, QsuB5, and QsuB15 were grown in a greenhouse as described in C.-Y. Lin *et al.*, 2022. Samples for transcriptomic and metabolomic analyses were collected following 5 months’ growth. Stems were cut 5 cm above the root collar and segments of 5 cm were collected above the first (bottom), 20th (middle), and 40th (top) internodes. The bark was peeled and developing xylem tissue was collected by scraping the surface of the debarked xylem segments using a sterile razor blade. This approach generated developing xylem samples from the older (bottom segment), intermediate (middle segment), and younger (top segment) parts of the stem (Supplementary Fig. S1A). The bark collected from the bottom stem segment was used to generate periderm (outer bark) and phloem (inner bark) tissue samples using a razor blade. Since the tissues were manually separated, some cork and vascular cambium tissues were possibly present in the phloem samples. All the samples were immediately placed in liquid nitrogen, and stored at −80 °C until time of use. Frozen samples were pulverized using a freezer mill (model 6875D, SPEX SamplePrep LLC, Metuchen, NJ, USA). Each sample was ground twice for 1 min at a grinding rate of 15 counts per second, including a 15 s break between the two cycles.

### RNA extraction, library preparation, and sequencing

Four biological replicates from each transgenic poplar line and WT control were used for large-scale transcriptomic analysis. Total RNA was extracted using the RNeasy Plant Mini Kit (Qiagen) and treated with RNAse-free DNAse. Total RNA was checked for integrity using a BioAnalyzer with an Agilent RNA 6000 Nano Chip, following the manufacturer’s instructions (Agilent). Plate-based RNA sample preparation was performed on the Sciclone NGS robotic liquid handling system (PerkinElmer), using the TruSeq Stranded mRNA HT sample prep kit with poly-A selection of mRNA following the manufacturer’s instructions (Illumina). Total RNA starting material was 1 μg per sample and eight PCR cycles were used for library amplification. The prepared libraries were then quantified by qPCR using the Kapa SYBR Fast Illumina Library Quantification Kit (Kapa Biosystems), and run on a LightCycler 480 real-time PCR instrument (Roche). The quantified libraries were prepared for sequencing on the Illumina HiSeq sequencing platform using a TruSeq paired-end cluster kit v4 and Illumina’s cBot instrument to generate a clustered flow cell for sequencing. Sequencing of the flow cell was performed on the Illumina HiSeq2500 sequencer using HiSeq TruSeq SBS sequencing kits v4 following a 2 × 150 indexed run procedure.

### RNA-seq data processing

Raw fastq file reads were filtered and trimmed using the Joint Genome Institute QC pipeline and BBDuk (https://sourceforge.net/projects/bbmap/). Raw reads were evaluated for artifact sequence by kmer matching (kmer=25), allowing one mismatch, and detected artifact trimmed from the 3ʹ end of the reads. RNA spike-in reads, PhiX reads, and reads containing any Ns were removed. Quality trimming was performed using the phred trimming method set at Q6. Finally, following trimming, reads under the length threshold were removed (minimum length 25 bases or a third of the original read length—whichever is longer). Filtered reads from each library were aligned to the *Populus trichocarpa* v4.1 reference genome (Tuskan *et al.*, 2006; https://phytozome-next.jgi.doe.gov/info/Ptrichocarpa_v4_1) using HISAT2 version 2.2.1 (Kim *et al.*, 2015). Strand-specific coverage bigWig files were generated using deepTools v3.1 (Ramírez *et al.*, 2014). FeatureCounts was used to generate the raw gene counts file using gff3 annotations (Liao *et al.*, 2014). Only primary hits assigned to the reverse strand were included in the raw gene counts.

### Metabolite extraction and liquid chromatography–mass spectrometry

To obtain metabolite extracts, frozen tissue powder was weighed and mixed with methanol at a ratio of 100 µl of solvent per mg of powder. Samples were vortexed for 1 min, then incubated at room temperature for 20 min with continuous mixing, centrifuged at 20 000×*g* for 5 min, and the supernatant filtered through 0.45 µm polytetrafluoroethylene filters. Three extraction control samples were obtained by following the same procedure, but without any tissue powder. Filtered metabolite extracts and controls were dried in a SpeedVac (Thermo Fisher Scientific, Waltham, MA, USA) and resuspended in 100% methanol containing an internal standard mix of isotopically labelled compounds (QC mix: ~15 µM average of 5–50 µM of ^13^C/^15^N cell free amino acid mixture; 10 µg ml^–1^ [^13^C]trehalose; 10 µg ml^–1^ [^13^C]mannitol; 2 µg ml^–1^ [^13^C/^15^N]uracil; 5.5 µg ml^–1^ [^15^N]inosine; 4 µg ml^–1^ [^15^N]adenine; 3 µg ml^–1^ [^15^N]hypoxanthine; 5 µg ml^–1^ [^13^C/^15^N]cytosine; 2.5 µg ml^–1^ [^13^C/^15^N]thymine; 1 µg ml^–1^ 2-amino-3-bromo-5-methylbenzoic acid), with resuspension volume of each varied to normalize by biomass for each sample.

UHPLC normal phase chromatography was performed using an Agilent 1290 LC stack, with MS and MS/MS data collected using a QExactive HF Orbitrap MS (Thermo Fisher Scientific). Full MS spectra were collected from *m*/*z* 70 to 1050 at 60k resolution in both positive and negative ionization mode, with MS/MS fragmentation data acquired using stepped then averaged 10, 20, and 40 eV collision energies at 15 000 resolution. Mass spectrometer source settings included a sheath gas flow rate of 55 (au), auxiliary gas flow of 20 (au), spray voltage of 3 kV (for both positive and negative ionization modes), and capillary temperature or 400 °C. For polar metabolites, normal phase chromatography was performed using a HILIC column (InfinityLab Poroshell 120 HILIC-Z, 2.1 × 150 mm, 2.7 µm, Agilent), at a flow rate of 0.45 ml min^–1^ with a 2 μl injection volume. The column was held at 40 °C equilibrated with 100% buffer B (99.8% 95:5 v/v ACN:H_2_O, 0.2% acetic acid, 5 mM ammonium acetate) for 1 min, diluting buffer B to 89% with buffer A (99.8% H_2_O, 0.2% acetic acid, 5 mM ammonium acetate, 5 µM methylene-di-phosphonic acid) over 10 min, down to 70% over 4.75 min, to 20% over 0.5 min, and finally isocratic elution for 2.25 min, followed by column re-equilibration by returning to 100% B over 0.1 min and isocratic elution for 3.9 min. For non-polar metabolites, reverse phase chromatography was performed using a C_18_ column (Zorbax Eclipse Plus C_18_, Rapid Resolution HD, 2.1 × 50 mm, 1.8 μm, Agilent) at a flow rate of 0.4 ml min^–1^ with a 2 μl injection volume. To detect metabolites with C_18_ chromatography, samples were run on the column at 60 °C equilibrated with 100% buffer A (100% H_2_O, 0.1% formic acid) for 1 min, diluting buffer A to 0% with buffer B (100% acetonitrile, 0.1% formic acid) over 7 min, and isocratic elution for 1.5 min, followed by column re-equilibration by returning to 100% A over 1 min and isocratic elution for 1 min. Samples consisted of four biological replicates each and extraction controls, with sample injection order randomized and an injection blank of 100% methanol run between each sample, with the blank replaced by an injection of internal standard mix every third sample, as well as QC mix every 15 samples. The raw data from the metabolite LC-MS runs are provided in Supplementary Dataset S1.

For data analysis, features with 0.5 min<retention time (RT)<9.5 min (C_18_ column) and 0.8 min<RT<18.5 min (HILC column), and max peak height fold-change between sample and extraction control >3 were selected. LC-MS data were analysed using custom Python code (Yao *et al.*, 2015), with each detected peak assigned a level of confidence indicated by a score from 0 to 3 in the compound identification. We performed a Feature-Based Molecular Networking (FBMN) workflow using MZmine 2 (Pluskal *et al.*, 2010) and Global Natural Products Social Molecular Networking (GNPS; http://gnps.ucsd.edu; Wang *et al.*, 2016). The MZmine workflow was used to generate a list of features obtained from extracted ion chromatograms containing chromatographic peaks within a narrow *m*/*z* range and filtered to remove isotopes. For each feature, the most intense fragmentation spectrum was uploaded to GNPS for putative identification by comparison with mass spectra deposited in the database. Compound classes were attributed to identified compounds using ClassyFire (Djoumbou Feunang *et al.*, 2016) or NPClassifier (Kim *et al.*, 2021). Putative metabolite structures were assigned based on RT and fragmentation patterns. For some metabolites, identification was based on exact mass and comparing RT and fragmentation spectra with those of standard compounds using the same LC-MS methods (Supplementary Dataset S2; Supplementary Table S1). For each compound, the measured masses agreed with the expected theoretical masses within less than 5 ppm mass error.

### Protein extraction and proteomic liquid chromatography–tandem mass spectrometry analysis

Tryptic peptides were prepared by following an established proteomic sample preparation protocol (Chen *et al*., 2023). Proteins were extracted from the xylem of intermediate stems by homogenizing frozen tissue powder (200 mg) with 500 µl of protein extraction buffer consisting of 50 mM Tris–HCl (pH 8.0), 100 mM NaCl, and 5 mM DTT in a centrifuge tube and using a clean pestle. Extracts were cleared by centrifugation (20 000×*g*) and proteins were precipitated with addition of 1 mM NaCl and 4 volumetric equivalents of acetone, followed by two additional washes with 80% acetone in water. The recovered protein pellet was homogenized by pipette mixing with 100 mM ammonium bicarbonate in 20% methanol. Protein concentration was determined by the DC protein assay (Bio-Rad Laboratories, Hercules, CA, USA). Protein reduction (15–20 µg total protein) was accomplished using 5 mM tris 2-(carboxyethyl)phosphine for 30 min at room temperature, and alkylation was performed with 10 mM iodoacetamide for 30 min at room temperature in the dark. Overnight digestion with trypsin was accomplished at a 1:50 trypsin:total protein ratio. The resulting peptide samples were analysed on an Agilent 1290 UHPLC system coupled to a Thermo Fisher Scientific Orbitrap Exploris 480 mass spectrometer for discovery proteomics (Chen *et al*., 2022). Briefly, peptide samples were loaded onto an Ascentis ES-C18 Column (Sigma-Aldrich, St Louis, MO, USA) and separated over a 10 min gradient from 98% solvent A (0.1 % formic acid in H_2_O) and 2% solvent B (0.1% formic acid in acetonitrile) to 65% solvent A and 35% solvent B. Eluting peptides were introduced to the mass spectrometer operating in positive-ion mode and were measured in data-independent acquisition (DIA) mode with a duty cycle of three survey scans from *m/z* 380 to *m/z* 985, and 45 MS2 scans with precursor isolation width of 13.5 *m/z* to cover the mass range. DIA raw data files were analysed by an integrated software suite DIA-NN (Demichev *et al*., 2020). The database used in the DIA-NN search (library-free mode) is the latest *Populus alba* (white poplar) UniProt proteome fasta sequences (UP000309997) plus the protein fasta sequences of QsuB (UniProt accession number Q8NT86) and common proteomic contaminants. DIA-NN determines mass tolerances automatically based on first pass analysis of the samples with automated determination of optimal mass accuracies. The retention time extraction window was determined individually for all MS runs analysed via the automated optimization procedure implemented in DIA-NN. Protein inference was enabled, and the quantification strategy was set to Robust LC=High Accuracy. Output main DIA-NN reports were filtered with a global false discovery rate (FDR)=0.01 on both the precursor level and protein group level. The sum of peak areas of identified tryptic peptides that derive from the QsuB protein was used to quantify the QsuB protein abundance in the samples. Four biological replicates were used for each genotype.

### Data analysis

Principal component analysis plots were obtained with MetaboAnalyst 5.0 available at https://www.metaboanalyst.ca/ (Pang *et al*., 2021) and venn diagrams were generated with the webtool available at https://bioinformatics.psb.ugent.be/webtools/Venn/. For the Gene Ontology (GO) enrichment analysis, the web-based g:Profiler tool was used to identify over-represented biological processes in the QsuB lines (Kolberg *et al*., 2023). The built-in algorithms of g:Profiler were used to correct for multiple testing and to identify the leading GO terms and remove the redundant terms in each function component. Leading GO terms that were significantly enriched (adjusted *P*-value≤0.05) in at least one QsuB line were visualized in a dot plot using the ggplot2 package v3.4.4 in R v4.3.1 (Wickham, 2009; R Core Team, 2018). For the Kyoto Encyclopedia of Genes and Genomes (KEGG) analysis, the *enrichKEGG* function with the clusterProfiler package v4.8.3 (Wu *et al*., 2021) in R was applied to identified over-represented KEGG categories in the QsuB lines based on the UniProt IDs of their differentially expressed genes. The *P*-value was adjusted using the FDR method with a cut-off of 0.1. The significantly enriched KEGG categories were visualized in a dot plot, as described above.

## Results

### Transcriptional changes in stem tissues of engineered QsuB poplar

**Fig. 2.**
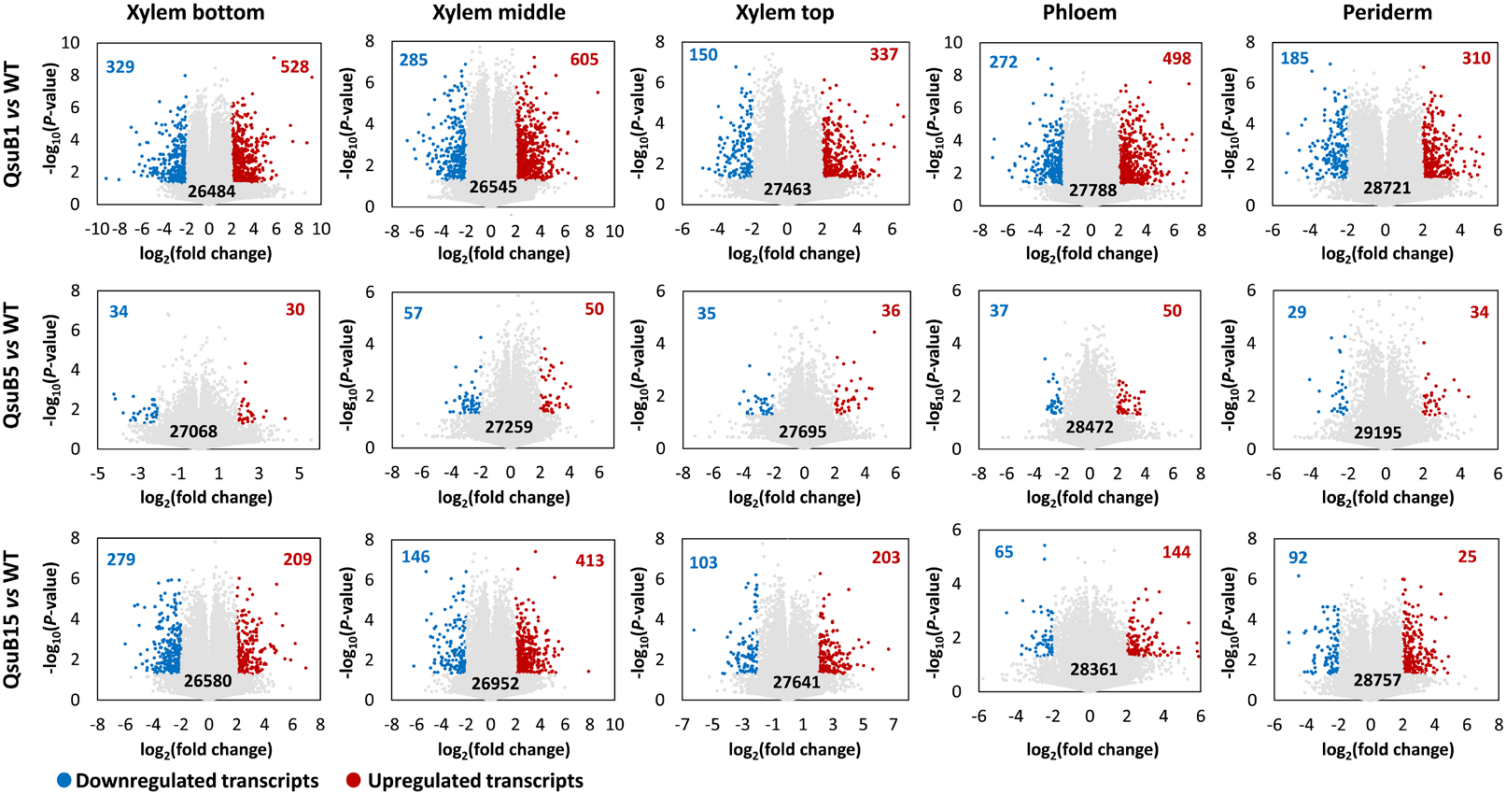
Volcano plots of transcripts identified in different stem tissues from wild type (WT) and QsuB transgenic lines. The number of down-regulated (in blue) and up-regulated (in red) transcripts in transgenics compared with WT control is indicated on each plot (log_2_ fold change +2/−2 and *P*-value<0.05). Grey dots represent transcripts not differentially expressed.

**Fig. 3.**
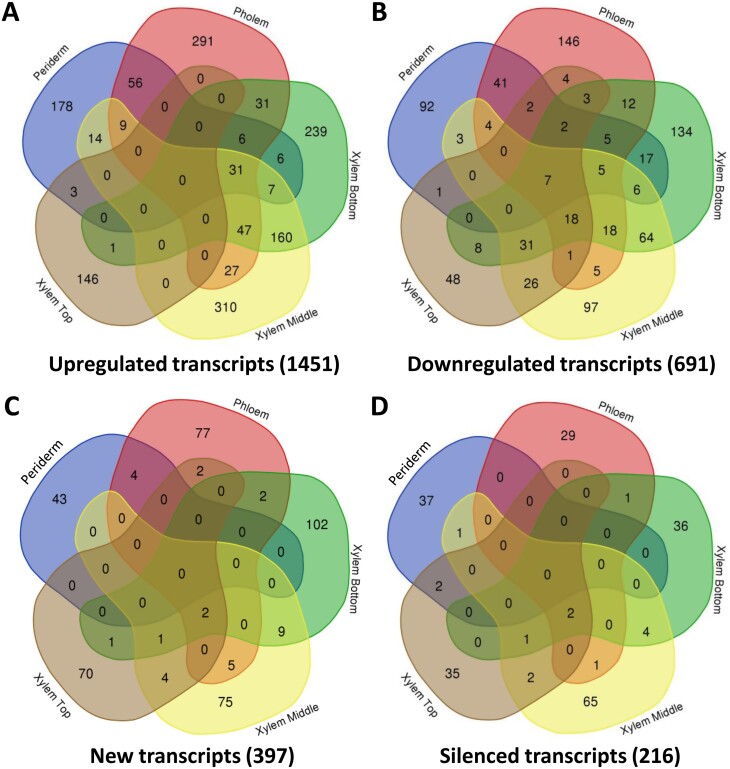
Venn diagrams of transcripts up-regulated (A), down-regulated (B), new (C), and silenced (D) in different tissues of line QsuB1. The number of unique transcripts is indicated in brackets.

The periderm and phloem tissues collected from the older part of the stem, as well as xylem tissues collected from the older, intermediate, and younger parts of the stem, were analysed by transcriptomics. Three selected genes known to be preferentially expressed in either the periderm (*PtCER1*, Rains *et al.*, 2018), or the phloem (*PtSEOR*, Chen *et al.*, 2021a), or the xylem (*PtCESA8*, Sundell *et al.*, 2017) were used to validate sampling quality (Supplementary Fig. S1B). The *QsuB* transcript levels in the transgenic lines were higher in the xylem tissues compared with the phloem and periderm, and line QsuB1 showed higher *QsuB* expression compared with QsuB15. *QsuB* transcripts levels were comparable in lines QsuB1 and QsuB5, which does not correlate with QsuB protein abundance in these lines and suggests reduced translation of the *QsuB* transcripts or lower QsuB protein stability in QsuB5 (Supplementary Fig. S1C). In WT control trees, principal component analysis (PCA) demonstrated a clear distinction between the periderm, phloem, and xylem tissues, whereas the xylem samples from older, intermediate, and younger stem sections were more similar to each other (Fig. 1C). A total of 31 082 transcripts were identified across all tissue types in WT, representing 88% of the 34 699 predicted protein-coding genes found in the poplar genome. The expression level of these genes in the different tissues is shown in Supplementary Table S2. We found transcripts specific to the periderm (602), phloem (242), and xylem (615 including 88, 106, and 143 transcripts specific to the xylem from older, intermediate, and younger stem sections, respectively), while 26 430 genes were found to be expressed in all tissue types (Fig. 1D). A list of tissue-specific transcripts identified in stems of the WT is provided in Supplementary Table S3. In each tissue, PCA of the transcripts permitted the discrimination of different genotypes and showed a separation between the low-lignin QsuB lines (QsuB1 and QsuB15), which grouped together, and QsuB5, which was closer to the WT (Supplementary Fig. S2). These differences were more important in xylem tissues compared with the phloem and periderm. In more detail, the data showed that line QsuB1 had the largest number of differentially expressed genes (DEGs) compared with WT in all the tissues analysed, followed by QsuB15 and QsuB5, which correlates with the levels of lignin reduction and DHBA accumulation in these lines (C.-Y. Lin *et al.*, 2022; Unda *et al.*, 2022) (Fig. 2; Supplementary Dataset S3). In the xylem from older stem sections of lines QsuB1 and QsuB15, a total of 857 (329 down-regulated, 528 up-regulated) and 488 (279 down-regulated, 309 up-regulated) transcripts were differentially expressed, respectively. These values indicate that 3.1% and 1.8% of the genes expressed in this tissue had a different expression level in QsuB1 and QsuB15 compared with WT, respectively. The smallest number of DEGs was found in the periderm in QsuB1, accounting for 495 transcripts (185 down-regulated, 310 up-regulated), while the phloem showed the fewest DEGs in QsuB15 (209 transcripts, 65 down-regulated, 144 upregulated) (Fig. 2). Line QsuB5 had a much smaller number of DEGs, with the xylem of intermediate stem sections displaying the highest count at 107 DEGs (Fig. 2). A list of the 10 most up-regulated and down-regulated transcripts in the different tissues of each QsuB line is provided in Supplementary Table S4. A majority of the DEGs were only observed in a specific tissue: In the case of QsuB1, 178, 291, and 856 transcripts were up-regulated specifically in the periderm, phloem, and xylem, respectively (Fig. 3A). Similarly, 92, 146, and 408 transcripts were down-regulated specifically in the aforementioned tissues (Fig. 3B). Moreover, new and silenced transcripts with distinct tissue-specific patterns were also observed in transgenics. In line QsuB1, 43, 77, and 262 new transcripts were detected in the periderm, phloem, and xylem, respectively (Fig. 3C), whereas 37, 29, and 143 silenced transcripts were identified in these tissues, respectively (Fig. 3D). Similar observations were made regarding the tissue specificity of up-regulated, down-regulated, new, and silenced transcripts identified in lines QsuB5 and QsuB15 (Supplementary Fig. S3; Supplementary Dataset S3).

**Fig. 4.**
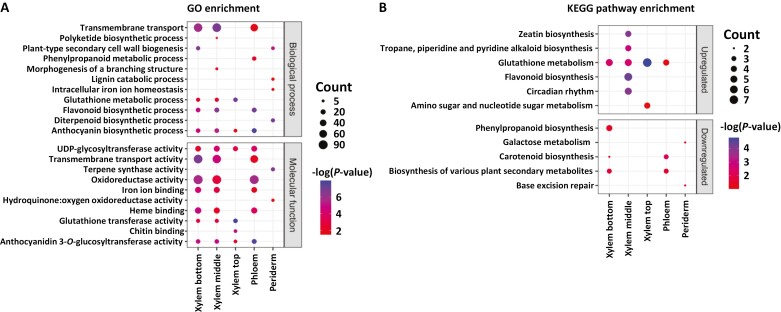
Dot plots of Gene Ontology (GO) (A) and Kyoto Encyclopedia of Genes and Genomes (KEGG) (B) enrichment analyses of differentially expressed genes identified in line QsuB1. The size of the dots represents the number of genes associated with each ontology term and pathway.

We performed GO and KEGG analyses to determine whether the DEGs identified in the transgenic plants were significantly enriched in specific biological processes, molecular functions, and metabolic pathways. Among DEGs found in lines QsuB1 and QsuB15, we noticed an enrichment in genes associated with transmembrane transport, oxidoreductase activity, glutathione metabolism, secondary metabolism (phenylpropanoids, flavonoids, anthocyanins, and cytokinins), glycosylation, iron binding, and secondary cell wall synthesis in the xylem and phloem tissues. DEGs in line QsuB5 were only enriched in genes linked to transport and glycosylation activity in the xylem (Fig. 4; Supplementary Fig. S4).

### Metabolic changes in stem tissues of engineered QsuB poplar

Separate aliquots of tissue samples used for transcriptome analysis were used for non-targeted metabolomics. We extracted metabolites with methanol and conducted metabolite profiling with a LC-MS platform that utilized gradient-based hydrophilic interaction liquid chromatography (HILIC) and reverse phase (C_18_) chromatography, in both the positive and the negative ionization modes.

**Fig. 5.**
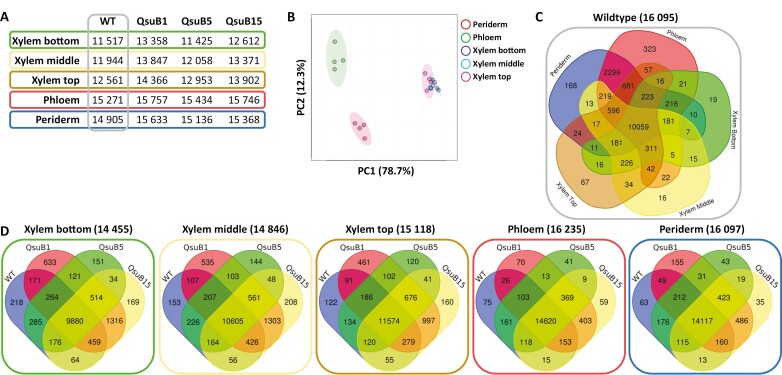
Features detected in wild type (WT) and transgenic QsuB poplar lines using hydrophilic interaction liquid chromatography (positive ionization mode). (A) Number of features detected in each tissue from the different lines. (B, C) Principal component analysis plot (B) and Venn diagram (C) of features detected in different tissues from WT stems. (D) Venn diagram of features detected in WT and QsuB lines for each tissue. The number of unique features is indicated in brackets for each tissue.

**Fig. 6.**
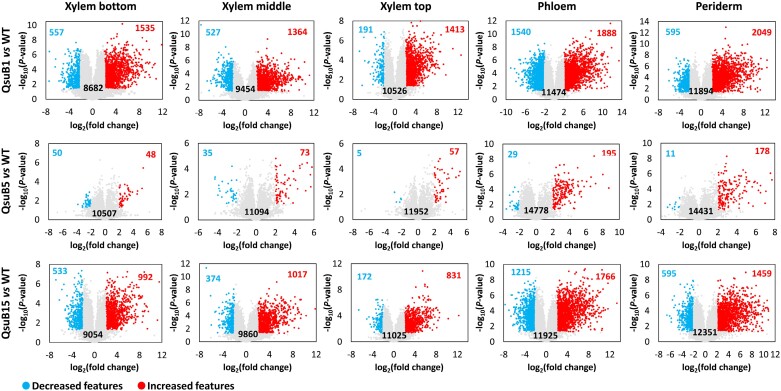
Volcano plots of features detected in different stem tissues from wild type (WT) and QsuB transgenic lines using hydrophilic interaction liquid chromatography (positive ionization mode). The number of decreased (in blue) and increased (in red) features in transgenic lines compared with WT control is indicated on each plot (log_2_ fold change +2/−2 and *P*-value<0.05). Grey dots represent features not differentially abundant.

**Fig. 7.**
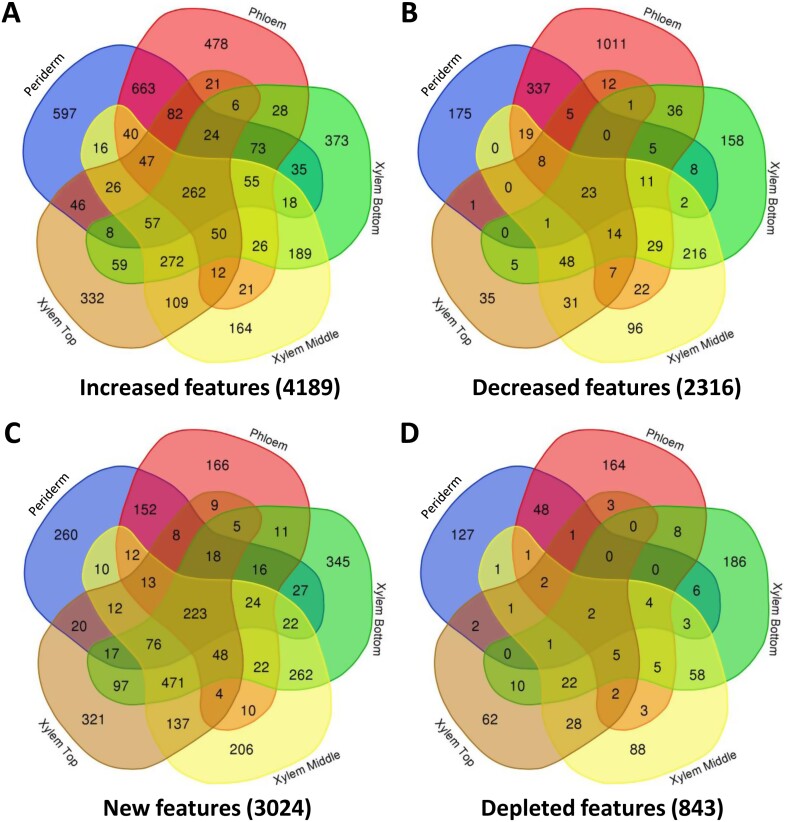
Venn diagrams of features more abundant (A), less abundant (B), new (C), and depleted (D) in different tissues of line QsuB1 (hydrophilic interaction liquid chromatography, positive ionization mode). The number of features is indicated in brackets.

**Fig. 8.**
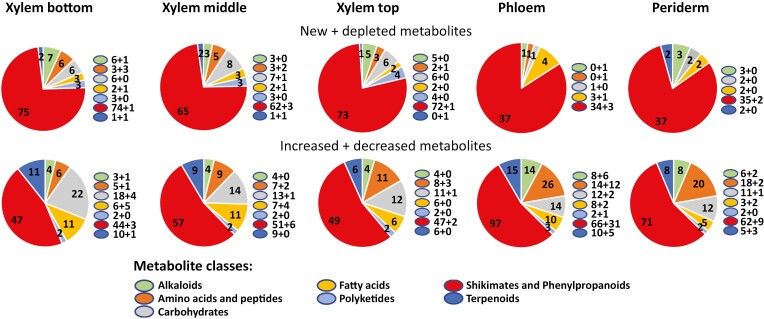
Classification of differentially abundant metabolites identified in each tissue of QsuB1 using hydrophilic interaction liquid chromatography (positive ionization mode). For each class, the number of metabolites is indicated inside the corresponding slice of the pie chart. A breakdown of new and depleted metabolites (upper panels) and of increased and decreased metabolites (lower panels) is indicated next to each colour class symbol.

A summary of the number of features detected in each stem tissue for each genotype using HILIC in the positive ionization mode is presented in Fig. 5A. In stem sections from WT trees, PCA showed a clear distinction between the periderm, phloem, and xylem tissues, whereas the xylem samples from older, intermediate, and younger stem sections were more similar to each other (Fig. 5B). A total of 16 095 features were identified in WT, including several specific to the periderm (168), phloem (323), and xylem (786 including 19, 16, and 67 features specific to the xylem from older, intermediate, and younger stems, respectively), whereas 10 059 features were detected in all tissue types (Fig. 5C). In every tissue examined, PCA grouped QsuB1 and QsuB15 together, separating them from the QsuB5/WT group (Supplementary Fig. S5). Most of the features detected in each tissue were present in all four genotypes, but some were only found in transgenics or the WT (Fig. 5D). In particular, 9880, 10 605, 11 574, 14 620, and 14 117 features were found in all genotypes in the xylem bottom, xylem middle, xylem top, phloem, and periderm stem tissues, respectively. A total of 10 774, 11 345, 12 130, 14 902, and 14 538 features were common to both WT and QsuB1 in the aforementioned tissues, respectively (Fig. 5D). We examined the relative abundance of these metabolites detected in both the WT control and transgenics (Fig. 6; Supplementary Dataset S4). In QsuB1, the line featuring the lowest lignin and highest DHBA content, we found that 19.4% (2092 out of 10 774) of the features detected in the xylem from older stem sections were differentially abundant compared with WT. This percentage was reduced to 16.7% and 13.2% in the xylem from intermediate and younger stems, respectively, but represented 23.0% in the phloem and 18.2% in the periderm (Fig. 6, top panels). In QsuB5 and QsuB15, the highest percentage of differentially abundant features was also found in the phloem (1.5% and 20.0%, respectively) (Fig.6, middle and bottom panels). Overall, more features increased than decreased in the transgenics, across all tissues (Fig. 6; Supplementary Dataset S4). We next focused on the distribution of the differentially abundant features across different tissues (Fig. 7; Supplementary Fig. S6). In QsuB1, a total of 4189 unique features were significantly more abundant compared with WT across all tissues combined, including 262 (6.3%) that were increased in all five tissue types, and 597, 478, and 1107 that were more abundant specifically in the periderm, phloem, and xylem, respectively (Fig. 7A). In contrast, 2316 unique features were significantly less abundant in QsuB1 in all tissues combined, including 23 (1.0%) that were reduced in all five tissue types, and others that were less abundant specifically in the periderm (175), phloem (1011), and xylem (589) (Fig. 7B). These results show that the highest number of more abundant features was found in the xylem, whereas the phloem contained the highest number of less abundant features when comparing QsuB1 to WT. The total number of unique, differentially abundant features was higher in QsuB1 (6505) compared with QsuB5 (492) and QsuB15 (5199). Several features were new or depleted (i.e. absent) across all tissue types in the different transgenic lines compared with WT (Fig. 7; Supplementary Fig. S6; Supplementary Dataset S4). In QsuB1, 223 new features were found in all tissue types (Fig. 7C), with only two features absent from all tissues analysed (Fig. 7D). The majority of these features were specific to the periderm (260 new/127 depleted), phloem (166 new/164 depleted), and xylem (1839 new/2293 depleted), with again some specificity within the xylem collected from old, intermediate, or young stem sections (Fig. 7C, D). Similarly, in line QsuB15, a large number of more abundant features (1033 out of 3158) was found specifically in the xylem, whereas the phloem contained the largest number of less abundant features (776 out of 2041), and the largest number of both new and depleted features was found in the xylem (Supplementary Fig. S6). QsuB15 showed fewer unique new features (2392) compared with QsuB1 (3024), but had a larger number of depleted features (942 *vs.* 843) (Fig. 7; Supplementary Fig. S6). Next, using GNPS, a subset of differentially abundant features observed in transgenics were classified and putatively identified. Consistent with the activity of QsuB within the shikimate pathway, a majority of the differentially abundant metabolites identified in transgenics were found to belong to the class of ‘shikimates and phenylpropanoids’ (Fig. 8; Supplementary Fig. S7). Finally, 10 962 unique features were detected across all stem tissues and genotypes using HILIC in the negative ionization mode. Their characteristics and the analysis of relative abundance and tissue distribution are presented in Supplementary Dataset S5 and Supplementary Figs S8–S10.

The analysis performed with reverse phase C_18_ chromatography resulted in the detection of 17 023 and 8512 unique features in the positive and negative ionization modes, respectively (Supplementary Datasets S6–S8). Comparably, the data indicate important metabolic changes in transgenic lines, including the presence of several new features and the absence of others, as well as important percentages of differentially abundant features. For example, compared with WT and across all five tissues, line QsuB1 had 3180 and 1652 new and depleted features, respectively. In the xylem of older stems, 21.7% and 17.7% of the detected features were differentially abundant in QsuB1 and QsuB15, respectively. These metabolites mainly belong to the class of ‘shikimates and phenylpropanoids’, as previously observed in the metabolite analysis conducted with HILIC.

### Impact of QsuB expression on the shikimate and lignin biosynthetic pathways

**Fig. 9.**
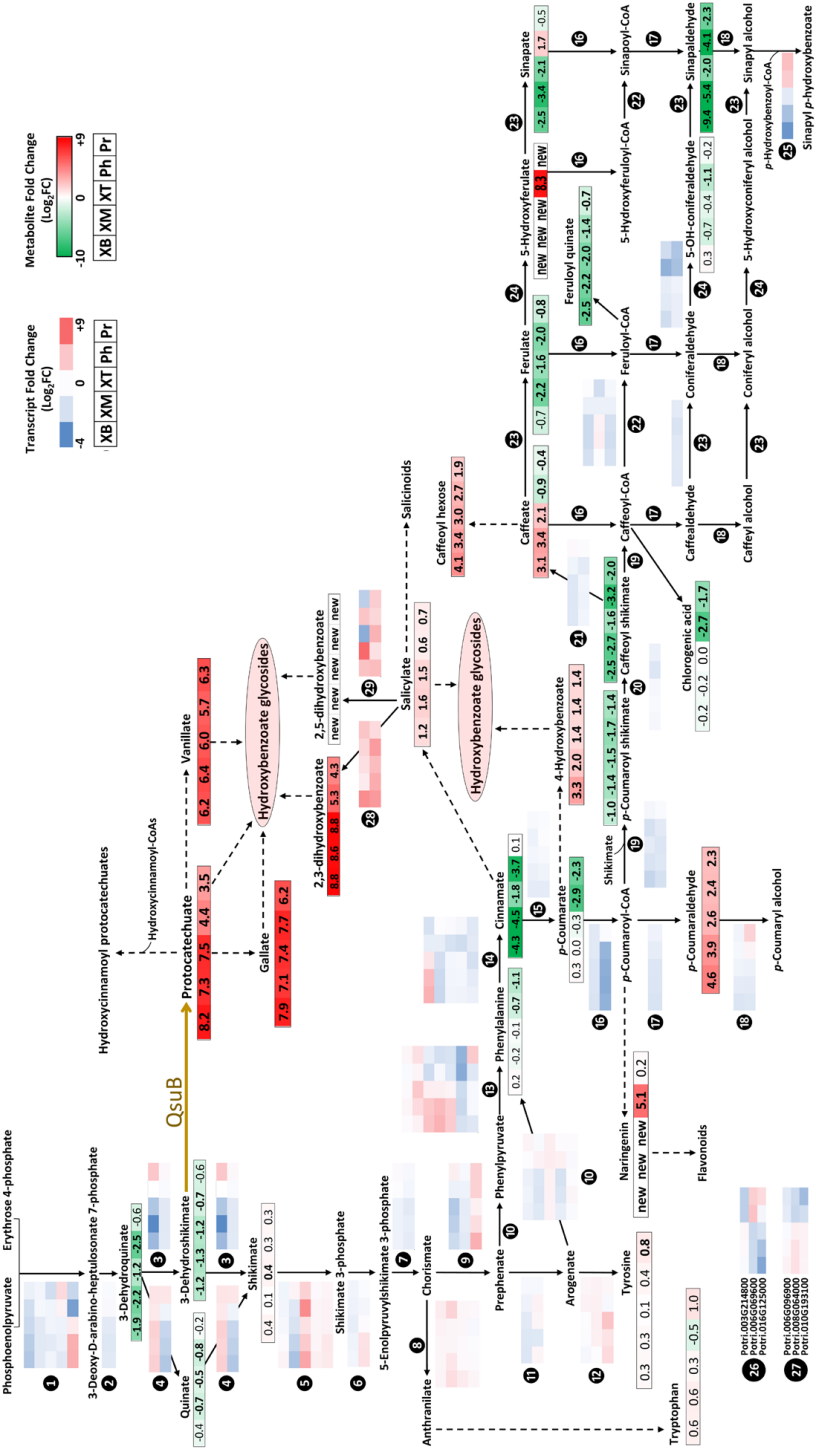
Transcriptional and metabolic changes in the shikimate and lignin pathways in QsuB poplar. Changes (log_2_ fold change) in transcripts and metabolites in different tissues of line QsuB1 are shown. Values in bold are significantly different from the WT (*P*<0.05). Transcript up-regulation is indicated as positive values (red) and down-regulation as negative values (blue). Increased and decreased metabolite abundances are shown in red and green, respectively. XB, XM, and XT, xylem tissue from older, intermediate, and younger sections of the stem, respectively; Ph, phloem; Pr, periderm. Black circled numbers denote enzymatic steps. Dashed arrows indicate multiple enzymatic steps or uncharacterized enzymes. ‘new’ indicates metabolites that are detected in QsuB1 but are below the detection limit in WT. See Supplementary Dataset S9 for detailed information on genes and metabolites, as well as the data obtained from lines QsuB5 and QsuB15.

We analysed specifically the metabolites and genes involved in the different steps of the shikimate and lignin pathways (Fig. 9; Supplementary Dataset S9). Notably, these pathways are responsible for the synthesis of the monolignols *p*-coumaryl, coniferyl, and sinapyl alcohols that give rise to the *p*-hydroxyphenyl (H), guaiacyl (G), and syringyl (S) subunits of lignin. Consistent with the enzymatic reaction catalysed by QsuB, large increases of DHBA and significant decreases of 3-dehydroshikimate were measured in the different stem tissues from lines QsuB1 and QsuB15. These changes were more important in the xylem tissues where QsuB expression was higher compared with the phloem and periderm (Supplementary Dataset S9). The content of 3-dehydroquinate, the precursor of 3-dehydroshikimate, was also reduced in the xylem and phloem, whereas shikimate was unchanged or even increased in the ‘xylem top’, despite the down-regulation of the two 3-dehydroquinate dehydratase/shikimate dehydrogenase genes (step 3, *Potri.013G029800* and *Potri.010G019000*). A conversion of 3-dehydroquinate into shikimate mediated by quinate dehydratase/dehydrogenase could explain these observations (step 4, *Potri.013G029900* and *Potri.005G043400*) (Guo *et al.*, 2014). Downstream of the shikimate pathway, the levels of the aromatic amino acids tryptophan, tyrosine, and phenylalanine were unchanged in the three xylem types. Nonetheless, a significant increase of tyrosine in the periderm and decrease of phenylalanine in the phloem and periderm was observed.

A major metabolic change in lines QsuB1 and QsuB15 was the increased levels of several metabolites derived from cinnamate and *p*-coumarate. 4-Hydroxybenzoate, a metabolite known to be synthesized from *p*-coumarate via β-oxidative and non-β oxidative routes, was higher in both lines. Salicylate, which is preferentially made from cinnamate in poplar (Ullah *et al*., 2023), and its derivatives 2,3-dihydroxybenzoate and 2,5-dihydroxybenzoate were also increased in the transgenics. The closest homologs of the Arabidopsis genes encoding salicylate 3-hydroxylase (*Potri.011G150100* and *Potri.011G150200*) and salicylate 5-hydroxylase (*Potri.015G002800* and *Potri.012G006300*) were overexpressed and thus represent strong candidates for salicylate hydroxylation in QsuB1 and QsuB15. The higher abundance of hydroxybenzoates and dihydroxybenzoates led to a large accumulation of multiple hydroxybenzoate and dihydroxybenzoate glycosides in QsuB poplar: 59 distinct metabolites featuring *m*/*z* and fragmentation patterns that are characteristic of hydroxybenzoates conjugated to sugar moieties were found to be increased or only detectable in QsuB1 and QsuB15 (Supplementary Dataset S9). A significant increase in caffeate was also apparent in the xylem from these two lines, possibly due to the overexpression of phenylalanine ammonia lyase (*Potri.006G126800*) and of the two putative *p*-coumarate 3-hydroxylase genes (Supplementary Dataset S9) (Barros *et al*., 2019), whereas ferulate and sinapate were reduced in this tissue, and, counterintuitively, 5-hydroxyferulate could only be detected in QsuB1 and QsuB15. In addition, metabolites that are derived from *p*-coumaroyl-CoA such as naringenin and *p*-coumaraldehyde were increased in the transgenic lines. Naringenin was only detected in lines QsuB1 and QsuB15 in the xylem, and showed a 32-fold increase in the phloem from these two lines. The increase of *p*-coumaraldehyde observed in all tissues from QsuB poplar is consistent with the higher content of H lignin units measured in transgenic trees, since they are derived from the polymerization of *p*-coumaryl alcohol (Unda *et al*., 2022). This change in lignin monomer composition is presumably the consequence of reduced hydroxycinnamoyl-CoA: shikimate hydroxycinnamoyl transferase (HCT) expression and/or activity. To support this hypothesis, we observed a down-regulation of the two HCT genes involved in lignin synthesis (step 19, *Potri.003G183900*/*PtHCT1* and *Potri.001G042900/PtHCT6*) and a reduction of the HCT product *p*-coumaroyl shikimate in the different tissues from QsuB1 and QsuB15 (Fig. 9). More generally, all the genes from the phenylpropanoid and monolignol pathways appeared down-regulated in most tissues analysed, except for one cinnamyl alcohol dehydrogenase gene (*Potri.016G078300*) that was up-regulated in the periderm and for the gene encoding *p*-hydroxybenzoyl-CoA monolignol transferase (*Potri.001G448000*/*pHBMT*; Zhao *et al*., 2021; de Vries *et al*., 2022) that was up-regulated in both the phloem and periderm. We examined the expression of peroxidase and laccase genes predicted to be involved in lignin polymerization and observed mixed expression profiles showing either up-regulation or down-regulation across the different tissues from QsuB1. For example, focusing on three peroxidase and three laccase genes that have been implicated genetically in lignification, we found inconsistent fold changes in expression when comparing laccases and peroxidases, and also when comparing the xylem tissues with phloem and periderm (steps 26/27 on Fig. 9; Supplementary Dataset S9).

## Discussion

The transcriptome and metabolome are commonly remodelled in response to transgene expression in plants. Our current results show that heterologous expression of a plastid-targeted bacterial 3-dehydroshikimate dehydratase induces noticeable transcriptional and metabolic changes in poplar (Figs 2, 6). In the developing xylem of the older stem section of a line displaying high QsuB expression, we found that 3% of the transcripts had altered expression levels and that ~19% of the detected metabolites had differential abundance. Similar observations were made in lignin-modified poplar lines that express a bacterial shikimate kinase and feature up to 2464 DEGs and 2025 differentially abundant metabolites (or ~27% of the total detected features) in the xylem compared with WT (Hu *et al.*, 2022). These results are consistent with the modification of secondary cell wall synthesis via overexpression of serine hydroxymethyltransferase, which resulted in the deregulation of up to 1159 genes in poplar leaves (Zhang *et al.*, 2019). In connection with the shikimate pathway, the heterologous expression of bacterial salicylate synthase targeted to plastids led to 1716 DEGs (~8% of the total transcripts) in poplar leaves (Xue *et al.*, 2013). Interestingly, field-grown engineered salt-tolerant poplar expressing a hormone-responsive transcription factor from tomato had 1.3% of DEGs in apical buds compared with WT, but this percentage was actually greater (~5% of the transcripts) when comparing identical poplar genotypes grown at two different field locations, indicating a large effect of environmental factors on gene expression (Zhang *et al.*, 2022). This observation is perhaps unsurprising considering that a single transient bending of the stem in poplar can affect the expression of ~6% of the transcripts (Pomiès *et al.*, 2017).

Alteration in the expression of multiple monolignol biosynthetic genes is often detected in poplar RNAi lines silenced for one particular gene of the lignin pathway, suggesting genetic interactions between these genes such as epistasis or other unknown higher-order regulation (Wang *et al.*, 2018; Matthews *et al.*, 2020). In the case of QsuB poplar, we observed a down-regulation of genes from the core phenylpropanoid and monolignol pathways, indicating that modifying the pools of 3-dehydroshikimate and DHBA (i.e. the substrate and product of QsuB, respectively) indirectly changes the expression of these genes. Such observations on gene down-regulation were also made in engineered poplar trees that express a bacterial shikimate kinase (Hu *et al.*, 2022). Shikimate and quinate are synthesized inside plastids, while the actual pools of shikimate and quinate available in the cytosol for enzymes such as HCT and hydroxycinnamoyl-CoA:quinate hydroxycinnamoyl transferase (HQT) remain undetermined in QsuB poplar. The reduction of caffeoyl quinate (chlorogenic acid) and feruloyl quinate in the xylem and phloem cells in transgenics could be the result of low quinate levels in the cytosol since *HQT* expression is unchanged (Fig. 9; Supplementary Dataset S9) (Zhang *et al.*, 2018). Previous work showed that silencing *PtHCT1* and *PtHCT6* reduces lignin and increases the relative amount of H units, similar to the lignin characteristics observed in QsuB poplar (Wang *et al*., 2018; Unda *et al.*, 2022). Besides its canonical substrate shikimate, PtHCT6 was shown to accept DHBA, 2,3-dihydroxybenzoate, and 2,5-dihydroxybenzoate (Eudes *et al*., 2016), suggesting that higher concentrations of these dihydroxybenzoates in the QsuB poplar lines could partially reduce HCT activity via competitive inhibition. The identification of features with mass ions matching those of *p*-coumaroyl dihydroxybenzoates and caffeoyl dihydroxybenzoates that are found to be more abundant or only detected in the QsuB lines supports this hypothesis (Fig. 9; Supplementary Dataset S9).

The occurrence of high amount of DHBA glycosides in QsuB poplar implies the export of DHBA from chloroplasts to the cytosol, the activity of UDP-glycosyltransferases (UGTs), and presumably the storage of DHBA conjugates in the vacuole, as previously described for other benzoates synthesized in plastids (Eudes *et al.*, 2008). Both ATP- and proton gradient-dependent transporters have been implicated in the uptake of benzoate glucose conjugates by plant vacuoles, but their identification is still pending (Bartholomew *et al.*, 2002; Vaca *et al.*, 2017). Very few plastidic transporters involved in the export of metabolites derived from the shikimate pathway have been characterized (Serrano *et al.*, 2013; Widhalm *et al.*, 2015), and whether DHBA produced in chloroplasts in QsuB poplar is exported via a transport protein remains unknown. Our transcriptomic data showed that DEGs in the QsuB lines are largely enriched in genes involved in transmembrane transport (Fig. 4; Supplementary Fig. S4). A gene encoding a proton-dependent transporter from the major facilitator superfamily (*Potri.018G041700*) and predicted to localize to plastids is among the 10 most overexpressed genes in all tissue types in QsuB1 and QsuB15 (Supplementary Table S4), making it a possible candidate involved in the non-specific export of DHBA. Moreover, we cannot exclude that DHBA export competes with the export of shikimate from plastids, hence affecting the cytosolic shikimate pool. Interestingly, results from previous studies suggest that the cytosolic shikimate pool could act as a sensor of the flux into lignin, and its reduction may favour the production of other phenylpropanoids (e.g. flavonoids) at the expense of lignin (Adams *et al*., 2019; Dixon and Barros, 2019). Such a redirection of the metabolic flux might also contribute to the overproduction of salicylates observed in QsuB poplar. DEGs were found to be significantly enriched in genes related to glycosyltransferase activity, including several encoding UGTs from the CAZy GT1 family (Fig. 4, Supplementary Fig. S4; Supplementary Table S4). UGT-7 (*Potri.007G132400*), UGT-37 (*Potri.016G016800*), and UGT-40 (*Potri.018G096000*), which are found among the 10 most overexpressed genes in QsuB1 and QsuB15, represent strong candidates for synthesizing DHBA and other benzoate glycosides in transgenics considering their known activity towards hydroxybenzoates (Saint-Vincent *et al.*, 2023).

We previously observed an increase of xylan content and changes in the meso-scale structure and organization of cellulose microfibrils in the cell walls of QsuB poplar, but the molecular basis for these observations is unclear (C-Y. Lin *et al.*, 2022; Unda *et al.*, 2022; Senanayake *et al.*, 2024). GO analysis retrieved 22 genes associated with ‘secondary cell wall biogenesis’ among the DEGs identified in the xylem from older stem sections of QsuB poplar (Fig. 4; yellow highlights in Supplementary Dataset S3). These include 10 fasciclin-like arabinogalactan protein (FLA) genes that are down-regulated in both QsuB1 (up to 60-fold) and QsuB15. The same FLA genes (*PtFLA1*–*PtFLA10*) were shown to be up-regulated in the tension wood produced in response to bending and characterized by a ‘G-layer’ of crystalline cellulose in fibre cells (Lafarguette *et al.*, 2004; Andersson-Gunnerås *et al.*, 2006). FLA genes have been implicated in cellulose synthesis and deposition in woody tissues, but their exact mechanisms of action remain unclear (MacMillan *et al.*, 2010; S. Lin *et al.*, 2022). Noteworthily, a recent study in Arabidopsis proposed that FLAs could act as cell surface sensors to fine-tune the balance of lignin and cellulose synthesis in secondary cell walls during development (Ma *et al*., 2022). In QsuB poplar, *PtFLA1*–*PtFLA10* are specifically down-regulated in the xylem from the bottom part of the stem, suggesting that changes induced by QsuB affect *PtFLA* expression only at the later stages of secondary cell wall deposition. Whether the modification and reduced crystallinity of cellulose observed in QsuB poplar results, in part, from the down-regulation of *PtFLA* genes warrants further investigation (Senanayake *et al.*, 2024). Moreover, a gene encoding a COBRA-Like (COBL) protein (*Potri.004G117200—COBL4*) is highly down-regulated in the ‘bottom xylem’ of transgenics QsuB1 and QsuB15 (Supplementary Dataset S3). COBL proteins are putative glycosylphosphatidylinositol-anchored proteins that control cellulose deposition and microfibril orientation in the cell wall, and *COBL4* is also known to be up-regulated during tension wood formation (Andersson-Gunnerås *et al.*, 2006; Liu *et al.*, 2021). Although glucan content is unchanged in QsuB poplar stems (C-Y. Lin *et al.*, 2022; Unda *et al*., 2022), one cellulose synthase-like (GT2) gene was up-regulated in the ‘bottom xylem’ in both QsuB1 and QsuB15, and one α-expansin gene was found overexpressed in this tissue in QsuB1 (Supplementary Dataset S3). Xyloglucan is another component of the G-layer in poplar tension wood fibre cells, and xyloglucan endo-transglycosylases (XETs) are generally up-regulated during G-layer formation (Nishikubo *et al.*, 2007). We found that one XET (GH16) and one putative xyloglucan fucosyltransferase (GT37) are strongly down-regulated (up to 74-fold) and up-regulated (up to 18-fold), respectively, in both QsuB1 and QsuB15 (Supplementary Dataset S3). Further, the up-regulation in the xylem of genes involved in cytokinin production may be part of a response to cell wall modifications considering the role of these hormones in vascular cambium development and secondary growth (Fig. 4B) (Nieminen *et al*., 2008). Collectively, these results also suggest that tension wood synthesis upon mechanical stress could be affected in QsuB poplar, in contrast with the observations made in low-lignin antisense 4-coumarate:CoA ligase (4CL) poplar trees that produce more tension wood (Voelker *et al.*, 2011). Three genes encoding GH3, CE13, and CE8 enzymes related to pectin synthesis were differentially expressed in the xylem of QsuB1 but not in QsuB15 (Supplementary Dataset S3), suggesting some indirect effects on pectin metabolism with higher QsuB expression levels.

The higher content of 4-hydroxybenzoate and its glycosides measured in methanolic extracts of QsuB poplar somehow coincides with the observed reductions in 4-hydroxybenzoate ester linked to lignin previously shown in the transgenics (Unda *et al*., 2022) (Fig. 9; Supplementary Dataset S9). One plausible explanation is the down-regulation in the xylem of the gene encoding the transferase responsible for these 4-hydroxybenzoate groups to be conjugated to monolignols, which can then be transported and participate in lignification (*p*HBMT, step 25 in Fig. 9). The preferential expression of *p*HBMT in the xylem as determined from our RNA-seq data is consistent with the occurrence of 4-hydroxybenzoate groups specifically in the cell walls of xylem fibers (Supplementary Dataset S3) (Goacher *et al*., 2021). *p*HBMT uses 4-hydroxybenzoyl-CoA and monolignols as substrates, but its activity towards dihydroxybenzoyl-CoA donors such as protocatechuyl-CoA has not been explored, and the transferase responsible for the formation of DHBA ester groups on lignin in QsuB poplar remains to be identified (Zhao *et al*., 2021; de Vries *et al*., 2022; Unda *et al*., 2022).

An increase of the stress hormone salicylate and salicinoids (i.e. grandidentatin, salicyloylsalicin, and tremulacin) were also observed in QsuB1 and QsuB15. Our RNA-seq data agree with other studies indicating a feedback inhibition of the shikimate pathway in modified poplar that features enhanced salicylate levels (Gordon *et al*., 2022). The higher levels of naringenin, taxifolin, myricetin, isorhamnetin, eriodictyol, and quercetin measured in QsuB transgenics are also consistent with previous observations that linked increases of flavonoids with higher salicylate levels (Supplementary Dataset S9) (Ullah *et al*., 2022). Notably, a few genes encoding transcriptional activators of flavonoid synthesis that are known to be induced by salicylate such as *MYB115* (*Potri.002G173900*), *MYB134* (*Potri.006G221800*), *bHLH131* (*Potri.005G208600*), and *WD40-1* (*Potri.016G075800*) are significantly overexpressed in the xylem of QsuB1 and/or QsuB15 (Ullah *et al*., 2019) (Supplementary Dataset S3). Moreover, an important enrichment of genes related to oxidoreductase, glutathione transferase, and chitinase activities among the DEGs is indicative of a stress state prevailing in QsuB poplar (Fig. 4; Supplementary Fig. S4). Iron is abundant in chloroplasts and essential for photosynthetic electron transport. DHBA produced in chloroplasts has the capacity to chelate ferric iron via its catechol moiety, which may disturb iron homeostasis, generate oxidative stress, and could explain the observed enrichment of genes involved in iron binding among the identified DEGs (Fig. 4; Supplementary Fig. S4). In this regard, specific secondary metabolites containing catechol moieties (e.g. coumarins) are typically secreted via root exudates to facilitate iron acquisition (Tsai and Schmidt, 2017), and possible changes in iron nutrition capacity remain to be explored in QsuB poplar. Considering that DHBA is a central intermediate in bacterial lignin catabolism and that lignin modifications can influence the endosphere microbiome and ectomycorrhizal colonization in poplar, it would be informative to study the microbiome in QsuB poplar (Beckers *et al.*, 2016; Kamimura *et al.*, 2017; Behr *et al.*, 2020).

The differential abundance in transgenics of select metabolites from the phenylpropanoid pathway, such as the accumulation of 5-hydroxyferulate and concomitant decrease of ferulate and sinapate, cannot be explained from the observed changes in gene expression of the biosynthetic enzymes. These differences may be due to post-transcriptional regulation mechanisms and various degrees of inhibition of the enzymes by several pathway intermediates, as shown previously for phenylalanine ammonia-lyase, 4CL, caffeate 3-*O*-methyltransferase, ferulate 5-hydroxylase, and cinnamyl alcohol dehydrogenase (Wang *et al*., 2014; Zhang *et al.*, 2020). Thus, metabolic flux analyses assisted by mathematical modelling that integrates these complex regulatory effects is needed to obtain a more comprehensive overview of the modified shikimate and lignin pathways in QsuB poplar (Wang *et al*., 2019; Rao and Barros, 2023).

Finally, the identification in this study of poplar transcripts that are specific to certain tissues (i.e. developing xylem at different growth stages, phloem, or periderm) provides concomitantly a comprehensive list of gene promoter candidates with wide range of activities that can be leveraged to achieve more precise spatiotemporal expression of transgenes in poplar stems (Supplementary Table S3). Fine-tuning transgene expression, notably with the design of synthetic promoters (Belcher *et al*., 2020; De Meester *et al*., 2021), should enable adequate expression of the desired engineered traits without compromising plant growth.

## Supplementary data

The following supplementary data are available at *JXB* online.

Fig. S1. Stem tissue sampling approach used in this study.

Fig. S2. PCA plots of the transcripts identified in WT and QsuB lines in each tissue.

Fig. S3. Venn diagrams of up-regulated, down-regulated, new, and silenced transcripts in different tissues of transgenic lines QsuB5 and QsuB15.

Fig. S4. Dot plots of GO and KEGG enrichment analyses of DEGs identified in line.

Fig. S5. PCA plots of the features detected in WT and QsuB lines in each tissue (HILIC, positive ionization mode).

Fig. S6. Venn diagrams of features more abundant, less abundant, new, and depleted in different tissues of lines QsuB5 and QsuB15 (HILIC positive ionization mode).

Fig. S7. Classification of differentially abundant metabolites identified in each tissue of QsuB5 and QsuB15 using HILIC (positive ionization mode).

Fig. S8. Features detected in WT and transgenic QsuB poplar lines using HILIC (negative ionization mode).

Fig. S9. Volcano plots of features detected in WT and QsuB transgenic lines in different stem tissues using HILIC (negative ionization mode).

Fig. S10. Venn diagrams of features more abundant, less abundant, new, and depleted in different tissues of the QsuB lines (HILIC, negative ionization mode).

Table S1. List and LC-MS characteristics of standard compounds used in this study.

Table S2. Gene expression levels of poplar genes (DESeq2 normalized counts) in the xylem, phloem, and periderm from stems of WT plants.

Table S3. List of tissue-specific transcripts identified in the periderm, phloem, and xylem from stems of WT poplar.

Table S4. Gene functional annotation of the 10 most highly up-regulated and down-regulated transcripts in the QsuB lines.

Dataset S1. Raw data from the metabolite LC-MS runs.

Dataset S2. Mirror plots of LC-MS standard compounds used in this study.

Dataset S3. List of DEGs in QsuB poplar.

Dataset S4. List of differentially abundant features in QsuB poplar (HILIC positive mode).

Dataset S5. List of differentially abundant features in QsuB poplar (HILIC negative mode).

Dataset S6. List of differentially abundant features in QsuB poplar (C_18_ positive mode).

Dataset S7. List of differentially abundant features in QsuB poplar (C_18_ negative mode).

Dataset S8. Nontargeted metabolomic analysis using reverse phase C_18_ chromatography

Dataset S9. Information on transcripts and metabolites displayed in Fig. 9.

erae251_suppl_Supplementary_Materials

## Data Availability

All data supporting the findings of this study are available within the paper and within its supplementary data published online. The RNA-seq raw datasets in this study are available at the NCBI Sequence Read Archive under BioProject IDs PRJNA1069121-PRJNA1069191 and PRJNA1069191-PRJNA1069222. The raw metabolite data can be accessed at the MassIVE public depository under the dataset identifier MSV000094089. The generated mass spectrometry proteomics data have been deposited to the ProteomeXchange Consortium via the PRIDE partner repository with the dataset identifier PXD049389.

## References

[CIT0001] Adams ZP , Ehlting J, Edwards R. 2019. The regulatory role of shikimate in plant phenylalanine metabolism. Journal of Theoretical Biology 462, 158–170. 30412698 10.1016/j.jtbi.2018.11.005

[CIT0002] An Y , Liu Y, Liu Y, Lu M, Kang X, Mansfield SD, Zeng W, Zhang J. 2021. Opportunities and barriers for biofuel and bioenergy production from poplar. GCB Bioenergy 13, 905–913.

[CIT0003] Andersson-Gunnerås S , Mellerowicz EJ, Love J, Segerman B, Ohmiya Y, Coutinho PM, Nilsson P, Henrissat B, Moritz T, Sundberg B. 2006. Biosynthesis of cellulose-enriched tension wood in *Populus*: global analysis of transcripts and metabolites identifies biochemical and developmental regulators in secondary wall biosynthesis. The Plant Journal 45, 144–165. 16367961 10.1111/j.1365-313X.2005.02584.x

[CIT0004] Barchet GLH , Dauwe R, Guy RD, Schroeder WR, Soolanayakanahally RY, Campbell MM, Mansfield SD. 2014. Investigating the drought-stress response of hybrid poplar genotypes by metabolite profiling. Tree Physiology 34, 1203–1219. 24178982 10.1093/treephys/tpt080

[CIT0005] Barros J , Escamilla-Trevino L, Song L, et al. 2019. 4-Coumarate 3-hydroxylase in the lignin biosynthesis pathway is a cytosolic ascorbate peroxidase. Nature Communications 10, 1994. 10.1038/s41467-019-10082-7 PMC6491607 31040279

[CIT0006] Bartholomew DM , Van Dyk DE, Lau S-MC, O’Keefe DP, Rea PA, Viitanen PV. 2002. Alternate energy-dependent pathways for the vacuolar uptake of glucose and glutathione conjugates. Plant Physiology 130, 1562–1572. 12428021 10.1104/pp.008334 PMC166675

[CIT0007] Beckers B , Op De Beeck M, Weyens N, Van Acker R, Van Montagu M, Boerjan W, Vangronsveld J. 2016. Lignin engineering in field-grown poplar trees affects the endosphere bacterial microbiome. Proceedings of the National Academy of Sciences, USA 113, 2312–2317. 10.1073/pnas.1523264113 PMC4776533 26755604

[CIT0008] Behr M , Baldacci-Cresp F, Kohler A, et al. 2020. Alterations in the phenylpropanoid pathway affect poplar ability for ectomycorrhizal colonisation and susceptibility to root-knot nematodes. Mycorrhiza 30, 555–566. 32647969 10.1007/s00572-020-00976-6

[CIT0009] Belcher MS , Vuu KM, Zhou A, Mansoori N, Agosto Ramos A, Thompson MG, Scheller HV, Loqué D, Shih PM. 2020. Design of orthogonal regulatory systems for modulating gene expression in plants. Nature Chemical Biology 16, 857–865. 32424304 10.1038/s41589-020-0547-4

[CIT0010] Chen M , Yin Y, Zhang L, Yang X, Fu T, Huo X, Wang Y. 2021b. Metabolomics and transcriptomics integration of early response of *Populus tomentosa* to reduced nitrogen availability. Frontiers in Plant Science 12, 769748. 34956269 10.3389/fpls.2021.769748 PMC8692568

[CIT0011] Chen Y , Gin J, Petzold CJ. 2022. Discovery proteomic (DIA) LC-MS/MS data acquisition and analysis v2. https://www.protocols.io/view/discovery-proteomic-dia-lc-ms-ms-data-acquisition-e6nvwk1z7vmk/v2

[CIT0012] Chen Y , Gin JW, Wang Y, de Raad M, Tan S, Hillson NJ, Northen TR, Adams PD, Petzold CJ. 2023. Alkaline-SDS cell lysis of microbes with acetone protein precipitation for proteomic sample preparation in 96-well plate format. PLoS One 18, e0288102. 37418444 10.1371/journal.pone.0288102 PMC10328223

[CIT0013] Chen Y , Tong S, Jiang Y, et al. 2021a. Transcriptional landscape of highly lignified poplar stems at single-cell resolution. Genome Biology 22, 319. 34809675 10.1186/s13059-021-02537-2 PMC8607660

[CIT0014] Dauwe R , Holliday JA, Aitken SN, Mansfield SD. 2012. Metabolic dynamics during autumn cold acclimation within and among populations of Sitka spruce (*Picea sitchensis*). New Phytologist 194, 192–205. 22248127 10.1111/j.1469-8137.2011.04027.x

[CIT0015] De Meester B , Vanholme R, de Vries L, Wouters M, Van Doorsselaere J, Boerjan W. 2021. Vessel- and ray-specific monolignol biosynthesis as an approach to engineer fiber-hypolignification and enhanced saccharification in poplar. The Plant Journal 108, 752–765. 34403547 10.1111/tpj.15468

[CIT0016] Demichev V , Messner CB, Vernardis SI, Lilley KS, Ralser M. 2020. DIA-NN: neural networks and interference correction enable deep proteome coverage in high throughput. Nature Methods 17, 41–44. 31768060 10.1038/s41592-019-0638-x PMC6949130

[CIT0017] de Vries L , Brouckaert M, Chanoca A, et al. 2021a. CRISPR-Cas9 editing of CAFFEOYL SHIKIMATE ESTERASE 1 and 2 shows their importance and partial redundancy in lignification in *Populus tremula* × *P. alba*. Plant Biotechnology Journal 19, 2221–2234. 34160888 10.1111/pbi.13651 PMC8541784

[CIT0018] de Vries L , Guevara-Rozo S, Cho M, Liu L-Y, Renneckar S, Mansfield SD. 2021b. Tailoring renewable materials via plant biotechnology. Biotechnology for Biofuels 14, 167. 34353358 10.1186/s13068-021-02010-z PMC8344217

[CIT0019] de Vries L , MacKay HA, Smith RA, et al. 2022. pHBMT1, a BAHD-family monolignol acyltransferase, mediates lignin acylation in poplar. Plant Physiology 188, 1014–1027. 34977949 10.1093/plphys/kiab546 PMC8825253

[CIT0020] Dixon RA , Barros J. 2019. Lignin biosynthesis: old roads revisited and new roads explored. Open biology 9, 190215. 31795915 10.1098/rsob.190215 PMC6936255

[CIT0021] Djoumbou Feunang Y , Eisner R, Knox C, et al. 2016. ClassyFire: automated chemical classification with a comprehensive, computable taxonomy. Journal of Cheminformatics 8, 61. 27867422 10.1186/s13321-016-0174-y PMC5096306

[CIT0022] Dong N-Q , Lin H-X. 2021. Contribution of phenylpropanoid metabolism to plant development and plant-environment interactions. Journal of Integrative Plant Biology 63, 180–209. 33325112 10.1111/jipb.13054

[CIT0023] Eudes A , Bozzo GG, Waller JC, Naponelli V, Lim E-K, Bowles DJ, Gregory JF, Hanson AD. 2008. Metabolism of the folate precursor *p*-aminobenzoate in plants: glucose ester formation and vacuolar storage. The Journal of Biological Chemistry 283, 15451–15459. 18385129 10.1074/jbc.M709591200 PMC2397476

[CIT0024] Eudes A , Pereira JH, Yogiswara S, Wang G, Teixeira Benites V, Baidoo EEK, Lee TS, Adams PD, Keasling JD, Loqué D. 2016. Exploiting the substrate promiscuity of hydroxycinnamoyl-CoA:shikimate hydroxycinnamoyl transferase to reduce lignin. Plant & Cell Physiology 57, 568–579. 26858288 10.1093/pcp/pcw016 PMC4790474

[CIT0025] Eudes A , Sathitsuksanoh N, Baidoo EEK, George A, Liang Y, Yang F, Singh S, Keasling JD, Simmons BA, Loqué D. 2015. Expression of a bacterial 3-dehydroshikimate dehydratase reduces lignin content and improves biomass saccharification efficiency. Plant Biotechnology Journal 13, 1241–1250. 25583257 10.1111/pbi.12310 PMC6680230

[CIT0026] Goacher RE , Mottiar Y, Mansfield SD. 2021. ToF-SIMS imaging reveals that *p*-hydroxybenzoate groups specifically decorate the lignin of fibres in the xylem of poplar and willow. Holzforschung 75, 452462.

[CIT0027] Gordon H , Fellenberg C, Lackus ND, Archinuk F, Sproule A, Nakamura Y, Llner TGK, Gershenzon J, Overy DP, Constabel CP. 2022. CRISPR/Cas9 disruption of UGT71L1 in poplar connects salicinoid and salicylic acid metabolism and alters growth and morphology. The Plant Cell 34, 2925–2947. 35532172 10.1093/plcell/koac135 PMC9338807

[CIT0028] Guo J , Carrington Y, Alber A, Ehlting J. 2014. Molecular characterization of quinate and shikimate metabolism in *Populus trichocarpa*. The Journal of Biological Chemistry 289, 23846–23858. 24942735 10.1074/jbc.M114.558536 PMC4156088

[CIT0029] Hamanishi ET , Barchet GLH, Dauwe R, Mansfield SD, Campbell MM. 2015. Poplar trees reconfigure the transcriptome and metabolome in response to drought in a genotype- and time-of-day-dependent manner. BMC Genomics 16, 329. 25895923 10.1186/s12864-015-1535-z PMC4437445

[CIT0030] Hinchee M , Rottmann W, Mullinax L, Zhang C, Chang S, Cunningham M, Pearson L, Nehra N. 2009. Short-rotation woody crops for bioenergy and biofuels applications. In Vitro Cellular & Developmental Biology 45, 619–629. 10.1007/s11627-009-9235-5 PMC2778772 19936031

[CIT0031] Hu S , Kamimura N, Sakamoto S, et al. 2022. Rerouting of the lignin biosynthetic pathway by inhibition of cytosolic shikimate recycling in transgenic hybrid aspen. The Plant Journal 110, 358–376. 35044002 10.1111/tpj.15674

[CIT0032] Kaling M , Kanawati B, Ghirardo A, Albert A, Winkler JB, Heller W, Barta C, Loreto F, Schmitt-Kopplin P, Schnitzler J-P. 2015. UV-B mediated metabolic rearrangements in poplar revealed by non-targeted metabolomics. Plant, Cell & Environment 38, 892–904. 10.1111/pce.12348 24738572

[CIT0033] Kamimura N , Takahashi K, Mori K, Araki T, Fujita M, Higuchi Y, Masai E. 2017. Bacterial catabolism of lignin-derived aromatics: New findings in a recent decade: Update on bacterial lignin catabolism. Environmental Microbiology Reports 9, 679–705. 29052962 10.1111/1758-2229.12597

[CIT0034] Kim D , Langmead B, Salzberg SL. 2015. HISAT: a fast spliced aligner with low memory requirements. Nature Methods 12, 357–360. 25751142 10.1038/nmeth.3317 PMC4655817

[CIT0035] Kim HW , Wang M, Leber CA, Nothias L-F, Reher R, Kang KB, van der Hooft JJJ, Dorrestein PC, Gerwick WH, Cottrell GW. 2021. NPClassifier: A deep neural network-based structural classification tool for natural products. Journal of Natural Products 84, 2795–2807. 34662515 10.1021/acs.jnatprod.1c00399 PMC8631337

[CIT0036] Kolberg L , Raudvere U, Kuzmin I, Adler P, Vilo J, Peterson H. 2023. g:Profiler-interoperable web service for functional enrichment analysis and gene identifier mapping. Nucleic Acids Research 51, W207–W212. 37144459 10.1093/nar/gkad347 PMC10320099

[CIT0037] Lafarguette F , Leplé J-C, Déjardin A, Laurans F, Costa G, Lesage-Descauses M-C, Pilate G. 2004. Poplar genes encoding fasciclin-like arabinogalactan proteins are highly expressed in tension wood. New Phytologist 164, 107–121. 33873473 10.1111/j.1469-8137.2004.01175.x

[CIT0038] Langholtz MH , Stokes BJ, Eaton LM. 2016. 2016 Billion-ton report: Advancing domestic resources for a thriving bioeconomy. Oak Ridge, TN: U.S. Department of Energy Office of Scientific and Technical Information.

[CIT0039] Liao Y , Smyth GK, Shi W. 2014. featureCounts: an efficient general purpose program for assigning sequence reads to genomic features. Bioinformatics 30, 923–930. 24227677 10.1093/bioinformatics/btt656

[CIT0040] Lin C-Y , Geiselman GM, Liu D, et al. 2022. Evaluation of engineered low-lignin poplar for conversion into advanced bioproducts. Biotechnology for Biofuels and Bioproducts 15, 145. 36567331 10.1186/s13068-022-02245-4 PMC9790118

[CIT0041] Lin S , Miao Y, Huang H, Zhang Y, Huang L, Cao J. 2022. Arabinogalactan proteins: Focus on the role in cellulose synthesis and deposition during plant cell wall biogenesis. International Journal of Molecular Sciences 23, 6578. 35743022 10.3390/ijms23126578 PMC9223364

[CIT0042] Liu B , Liu J, Yu J, Wang Z, Sun Y, Li S, Lin Y-CJ, Chiang VL, Li W, Wang JP. 2021. Transcriptional reprogramming of xylem cell wall biosynthesis in tension wood. Plant Physiology 186, 250–269. 33793955 10.1093/plphys/kiab038 PMC8154086

[CIT0043] Liu C-J , Eudes A. 2022. Lignin synthesis and bioengineering approaches toward lignin modification. Advances in Botanical Research 104, 41–96.

[CIT0044] Ma Y , MacMillan CP, de Vries L, Mansfield SD, Hao P, Ratcliffe J, Bacic A, Johnson KL. 2022. FLA11 and FLA12 glycoproteins fine-tune stem secondary wall properties in response to mechanical stresses. New Phytologist 233, 1750–1767. 34862967 10.1111/nph.17898 PMC9302641

[CIT0045] MacMillan CP , Mansfield SD, Stachurski ZH, Evans R, Southerton SG. 2010. Fasciclin-like arabinogalactan proteins: specialization for stem biomechanics and cell wall architecture in Arabidopsis and *Eucalyptus*. The Plant Journal 62, 689–703. 20202165 10.1111/j.1365-313X.2010.04181.x

[CIT0046] Maeda H , Dudareva N. 2012. The shikimate pathway and aromatic amino acid biosynthesis in plants. Annual Review of Plant Biology 63, 73–105. 10.1146/annurev-arplant-042811-105439 22554242

[CIT0047] Mahon EL , Mansfield SD. 2019. Tailor-made trees: engineering lignin for ease of processing and tomorrow’s bioeconomy. Current Opinion in Biotechnology 56, 147–155. 30529238 10.1016/j.copbio.2018.10.014

[CIT0048] Matthews ML , Wang JP, Sederoff R, Chiang VL, Williams CM. 2020. Modeling cross-regulatory influences on monolignol transcripts and proteins under single and combinatorial gene knockdowns in *Populus trichocarpa*. PLoS Computational Biology 16, e1007197. 32275650 10.1371/journal.pcbi.1007197 PMC7147730

[CIT0049] Mizrachi E , Mansfield SD, Myburg AA. 2012. Cellulose factories: advancing bioenergy production from forest trees. New Phytologist 194, 54–62. 22474687 10.1111/j.1469-8137.2011.03971.x

[CIT0050] Nieminen K , Immanen J, Laxell M, et al. 2008. Cytokinin signaling regulates cambial development in poplar. Proceedings of the National Academy of Sciences, USA 105, 20032–20037. 10.1073/pnas.0805617106 PMC2604918 19064928

[CIT0051] Nishikubo N , Awano T, Banasiak A, et al. 2007. Xyloglucan endo-transglycosylase (XET) functions in gelatinous layers of tension wood fibers in poplar—A glimpse into the mechanism of the balancing act of trees. Plant & Cell Physiology 48, 843–855. 17504814 10.1093/pcp/pcm055

[CIT0052] Pang Z , Chong J, Zhou G, de Lima Morais DA, Chang L, Barrette M, Gauthier C, Jacques P-E, Li S, Xia J. 2021. MetaboAnalyst 5.0: narrowing the gap between raw spectra and functional insights. Nucleic Acids Research 49, W388–W396. 34019663 10.1093/nar/gkab382 PMC8265181

[CIT0054] Pluskal T , Castillo S, Villar-Briones A, Oresic M. 2010. MZmine 2: modular framework for processing, visualizing, and analyzing mass spectrometry-based molecular profile data. BMC Bioinformatics 11, 395. 20650010 10.1186/1471-2105-11-395 PMC2918584

[CIT0055] Pomiès L , Decourteix M, Franchel J, Moulia B, Leblanc-Fournier N. 2017. Poplar stem transcriptome is massively remodelled in response to single or repeated mechanical stimuli. BMC Genomics 18, 300. 28412928 10.1186/s12864-017-3670-1 PMC5392906

[CIT0056] Qu C , Zuo Z, Cao L, Huang J, Sun X, Zhang P, Yang C, Li L, Xu Z, Liu G. 2019. Comprehensive dissection of transcript and metabolite shifts during seed germination and post-germination stages in poplar. BMC Plant Biology 19, 279. 31242858 10.1186/s12870-019-1862-3 PMC6595626

[CIT0057] R Core Team. 2018. R: a language and environment for statistical computing. Vienna, Austria: R Foundation for Statistical Computing. https://www.R-project.org/

[CIT0058] Rains MK , Gardiyehewa de Silva ND, Molina I. 2018. Reconstructing the suberin pathway in poplar by chemical and transcriptomic analysis of bark tissues. Tree Physiology 38, 340–361. 28575526 10.1093/treephys/tpx060

[CIT0059] Ramírez F , Dündar F, Diehl S, Grüning BA, Manke T. 2014. deepTools: a flexible platform for exploring deep-sequencing data. Nucleic Acids Research 42, W187–W191. 24799436 10.1093/nar/gku365 PMC4086134

[CIT0060] Rao X , Barros J. 2023. Modeling lignin biosynthesis: a pathway to renewable chemicals. Trends in Plant Science 29, 546–559. 37802691 10.1016/j.tplants.2023.09.011

[CIT0061] Robinson A , Beets P, Mansfield SD. 2022. Metabolite profiling reveals complex relationship between developing xylem metabolism and intra-ring checking in *Pinus radiata*. Holzforschung 76, 120–132.

[CIT0062] Robinson AR , Dauwe R, Mansfield SD. 2018. Assessing the between-background stability of metabolic effects arising from lignin-related transgenic modifications, in two *Populus* hybrids using non-targeted metabolomics. Tree Physiology 38, 378–396. 29040774 10.1093/treephys/tpx110

[CIT0063] Robinson AR , Dauwe R, Ukrainetz NK, Cullis IF, White R, Mansfield SD. 2009. Predicting the regenerative capacity of conifer somatic embryogenic cultures by metabolomics. Plant Biotechnology Journal 7, 952–963. 19906246 10.1111/j.1467-7652.2009.00456.x

[CIT0064] Robinson AR , Gheneim R, Kozak RA, Ellis DD, Mansfield SD. 2005. The potential of metabolite profiling as a selection tool for genotype discrimination in *Populus*. Journal of Experimental Botany 56, 2807–2819. 16143717 10.1093/jxb/eri273

[CIT0065] Robinson AR , Ukrainetz NK, Kang K-Y, Mansfield SD. 2007. Metabolite profiling of Douglas-fir (*Pseudotsuga menziesii*) field trials reveals strong environmental and weak genetic variation. New Phytologist 174, 762–773. 17504460 10.1111/j.1469-8137.2007.02046.x

[CIT0066] Saint-Vincent PMB , Furches A, Galanie S, et al. 2023. Validation of a metabolite-GWAS network for *Populus trichocarpa* family 1 UDP-glycosyltransferases. Frontiers in Plant Science 14, 1210146. 37546246 10.3389/fpls.2023.1210146 PMC10402742

[CIT0067] Senanayake M , Lin C-Y, Mansfield SD, Eudes A, Davison B, Pingali SV, O’Neill H. 2024. Ectopic production of 3,4-dihydroxybenzoate in planta affects cellulose structure and organization. Biomacromolecules. Biomacromolecules 25, 3542–3553. 38780531 10.1021/acs.biomac.4c00187

[CIT0068] Serrano M , Wang B, Aryal B, Garcion C, Abou-Mansour E, Heck S, Geisler M, Mauch F, Nawrath C, Métraux J-P. 2013. Export of salicylic acid from the chloroplast requires the multidrug and toxin extrusion-like transporter EDS5. Plant Physiology 162, 1815–1821. 23757404 10.1104/pp.113.218156 PMC3729763

[CIT0069] Sulis DB , Jiang X, Yang C, et al. 2023. Multiplex CRISPR editing of wood for sustainable fiber production. Science 381, 216–221. 37440632 10.1126/science.add4514 PMC10542590

[CIT0070] Sundell D , Street NR, Kumar M, et al. 2017. AspWood: high-spatial-resolution transcriptome profiles reveal uncharacterized modularity of wood formation in *Populus tremula*. The Plant Cell 29, 1585–1604. 28655750 10.1105/tpc.17.00153 PMC5559752

[CIT0071] Tsai HH , Schmidt W. 2017. Mobilization of iron by plant-borne coumarins. Trends in Plant Science 22, 538–548. 28385337 10.1016/j.tplants.2017.03.008

[CIT0072] Tuskan GA , Difazio S, Jansson S, et al. 2006. The genome of black cottonwood, *Populus trichocarpa* (Torr. & Gray). Science 313, 1596–1604. 16973872 10.1126/science.1128691

[CIT0073] Ullah C , Chen Y-H, Ortega MA, Tsai C-J. 2023. The diversity of salicylic acid biosynthesis and defense signaling in plants: Knowledge gaps and future opportunities. Current Opinion in Plant Biology 72, 102349. 36842224 10.1016/j.pbi.2023.102349

[CIT0074] Ullah C , Schmidt A, Reichelt M, Tsai C-J, Gershenzon J. 2022. Lack of antagonism between salicylic acid and jasmonate signalling pathways in poplar. New Phytologist 235, 701–717. 35489087 10.1111/nph.18148

[CIT0075] Ullah C , Tsai C-J, Unsicker SB, Xue L, Reichelt M, Gershenzon J, Hammerbacher A. 2019. Salicylic acid activates poplar defense against the biotrophic rust fungus *Melampsora larici-populina* via increased biosynthesis of catechin and proanthocyanidins. New Phytologist 221, 960–975. 30168132 10.1111/nph.15396 PMC6585937

[CIT0076] Umana GE , Perez JM, Unda F, et al. 2022. Biological funneling of phenolics from transgenic plants engineered to express the bacterial 3-dehydroshikimate dehydratase (*qsuB*) gene. Frontiers in Chemical Engineering 4, 1036084.

[CIT0077] Unda F , Mottiar Y, Mahon EL, Karlen SD, Kim KH, Loqué D, Eudes A, Ralph J, Mansfield SD. 2022. A new approach to zip-lignin: 3,4-dihydroxybenzoate is compatible with lignification. New Phytologist 235, 234–246. 35377486 10.1111/nph.18136 PMC9325543

[CIT0078] Vaca E , Behrens C, Theccanat T, Choe J-Y, Dean JV. 2017. Mechanistic differences in the uptake of salicylic acid glucose conjugates by vacuolar membrane-enriched vesicles isolated from *Arabidopsis thaliana*. Physiologia Plantarum 161, 322–338. 28665551 10.1111/ppl.12602

[CIT0080] Voelker SL , Lachenbruch B, Meinzer FC, Strauss SH. 2011. Reduced wood stiffness and strength, and altered stem form, in young antisense 4CL transgenic poplars with reduced lignin contents. New Phytologist 189, 1096–1109. 21158867 10.1111/j.1469-8137.2010.03572.x

[CIT0081] Wang JP , Matthews ML, Naik PP, Williams CM, Ducoste JJ, Sederoff RR, Chiang VL. 2019. Flux modeling for monolignol biosynthesis. Current Opinion in Biotechnology 56, 187–192. 30557780 10.1016/j.copbio.2018.12.003

[CIT0082] Wang JP , Matthews ML, Williams CM, et al. 2018. Improving wood properties for wood utilization through multi-omics integration in lignin biosynthesis. Nature Communications 9, 1579. 10.1038/s41467-018-03863-z PMC5910405 29679008

[CIT0083] Wang JP , Naik PP, Chen H-C, et al. 2014. Complete proteomic-based enzyme reaction and inhibition kinetics reveal how monolignol biosynthetic enzyme families affect metabolic flux and lignin in *Populus trichocarpa*. The Plant Cell 26, 894–914. 24619611 10.1105/tpc.113.120881 PMC4001400

[CIT0084] Wang M , Carver JJ, Phelan VV, et al. 2016. Sharing and community curation of mass spectrometry data with Global Natural Products Social Molecular Networking. Nature Biotechnology 34, 828–837. 10.1038/nbt.3597 PMC5321674 27504778

[CIT0079] Wickham H. 2009. ggplot2: Elegant graphics for data analysis. New York: Springer-Verlag.

[CIT0085] Widhalm JR , Gutensohn M, Yoo H, et al. 2015. Identification of a plastidial phenylalanine exporter that influences flux distribution through the phenylalanine biosynthetic network. Nature Communications 6, 8142. 10.1038/ncomms9142 PMC4647861 26356302

[CIT0086] Wu T , Hu E, Xu S, et al. 2021. clusterProfiler 4.0: a universal enrichment tool for interpreting omics data. Innovation 2, 100141. 34557778 10.1016/j.xinn.2021.100141 PMC8454663

[CIT0087] Xue L-J , Guo W, Yuan Y, et al. 2013. Constitutively elevated salicylic acid levels alter photosynthesis and oxidative state but not growth in transgenic *populus*. The Plant Cell 25, 2714–2730. 23903318 10.1105/tpc.113.112839 PMC3753393

[CIT0088] Yao Y , Sun T, Wang T, Ruebel O, Northen T, Bowen BP. 2015. Analysis of metabolomics datasets with high-performance computing and metabolite atlases. Metabolites 5, 431–442. 26287255 10.3390/metabo5030431 PMC4588804

[CIT0089] Zhang J , Li M, Bryan AC, et al. 2019. Overexpression of a serine hydroxymethyltransferase increases biomass production and reduces recalcitrance in the bioenergy crop *Populus*. Sustainable Energy and Fuels 3, 195–207.

[CIT0090] Zhang J , Tuskan GA, Tschaplinski TJ, Muchero W, Chen J-G. 2020. Transcriptional and post-transcriptional regulation of lignin biosynthesis pathway genes in *Populus*. Frontiers in Plant Science 11, 652. 32528504 10.3389/fpls.2020.00652 PMC7262965

[CIT0091] Zhang J , Yang Y, Zheng K, et al. 2018. Genome-wide association studies and expression-based quantitative trait loci analyses reveal roles of HCT2 in caffeoylquinic acid biosynthesis and its regulation by defense-responsive transcription factors in *Populus*. New Phytologist 220, 502–516. 29992670 10.1111/nph.15297

[CIT0092] Zhang L , Bao H, Meng F, Ren Y, Tian C. 2023. Transcriptome and metabolome reveal the role of flavonoids in poplar resistance to poplar anthracnose. Industrial Crops and Products 197, 116537.

[CIT0093] Zhang W , Wang Y, Zhang T, Zhang J, Shen L, Zhang B, Ding C, Su X. 2022. Transcriptomic analysis of mature transgenic poplar expressing the transcription factor JERF36 gene in two different environments. Frontiers in Bioengineering and Biotechnology 10, 929681. 35774064 10.3389/fbioe.2022.929681 PMC9237257

[CIT0094] Zhao X , Li P, Liu X, Xu T, Zhang Y, Meng H, Xia T. 2022. High temperature increased lignin contents of poplar (*Populus* spp) stem via inducing the synthesis caffeate and coniferaldehyde. Frontiers in Genetics 13, 1007513. 36160001 10.3389/fgene.2022.1007513 PMC9500204

[CIT0095] Zhao Y , Yu X, Lam P-Y, Zhang K, Tobimatsu Y, Liu C-J. 2021. Monolignol acyltransferase for lignin *p*-hydroxybenzoylation in *Populus*. Nature Plants 7, 1288–1300. 34354261 10.1038/s41477-021-00975-1

