# Supplementary material for: Expression of dehydroshikimate dehydratase in poplar induces transcriptional and metabolic changes in the phenylpropanoid pathway
Source: J Exp Bot. 2024 May 29;75(16):4960–77. doi: 10.1093/jxb/erae251 (PMC11349870; doi:10.1093/jxb/erae251)
Supplement: erae251_suppl_Supplementary_Materials [file erae251_suppl_supplementary_materials.zip › erae251_suppl_Supplementary_Dataset_S2.pdf]

Heterologous expression in poplar of dehydroshikimate dehydratase induces transcriptional and metabolic changes in phenylpropanoid pathway

Emine Akyuz Turumtay[1,2,3], Halbay Turumtay[1,2,4], Yang Tian[1,2], Chien-Yuan Lin[1,2], Yen Ning Chai[1,2], Katherine B. Louie[2,5], Yan Chen[1,6], Anna Lipzen[5], Thomas Harwood[2,5], Kavitha Satish Kumar[1,2], Benjamin P. Bowen[2,5], Qian Wang[7,8], Shawn D. Mansfield[7,8,9], Matthew J. Blow[5], Christopher J. Petzold[1,6], Trent R. Northen[2,5], Jenny C. Mortimer[1,2,10], Henrik V. Scheller[1,2,11], Aymerick Eudes[1,2,*]

[1] Feedstocks Division, Joint BioEnergy Institute, Emeryville, CA, USA
[2] Environmental Genomics and Systems Biology Division, Lawrence Berkeley National Laboratory, Berkeley, CA, USA
[3] Recep Tayyip Erdogan University, Department of Chemistry, 53100, Rize, Turkiye
[4] Karadeniz Technical University, Department of Energy System Engineering, 61830, Trabzon, Turkiye
[5] Joint Genome Institute, Lawrence Berkeley National Laboratory, Berkeley, CA, United States
[6] Biological Systems & Engineering Division, Lawrence Berkeley National Laboratory, Berkeley, CA, USA
[7] Department of Wood Science, University of British Columbia, Vancouver, BC, Canada
[8] Department of Botany, University of British Columbia, Vancouver, BC, Canada
[9] DOE Great Lakes Bioenergy Research Center, Wisconsin Energy Institute, Madison, WI 53726, USA
[10] School of Agriculture, Food and Wine & Waite Research Institute, University of Adelaide, Glen Osmond, SA, Australia.
[11] Department of Plant and Microbial Biology, University of California, Berkeley, Berkeley, CA, USA

*Correspondence: Aymerick Eudes, ageudes@lbl.gov

Supplementary Dataset S2

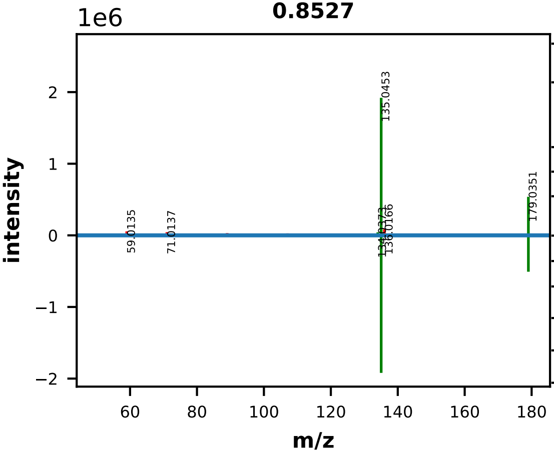

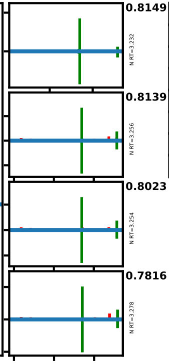

	EMA Compound Info
Name:	caffeic acid
Label:	
Formula:	C9H8O4
Polarity:	negative
Monoisotopic Mass:	180.042258736
Theoretical M/Z:	179.0349587
Adduct:	[M-H]-

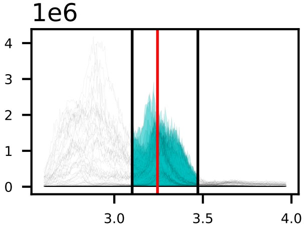

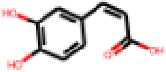

20220926_JGI_AE_507651_Poplar_final_QE-HF_HILICZ_USHXG01885_NEG_MSMS_114_Qsub5-Pholem-whole_C_Rg70to1050-CE102040--S1_Run198.h5

0025_caffeic_acid_negative_M-H179p0350_3p24 [M-H]-
Measured M/Z = 179.0349, 0.4274 ppm difference
Expected Elution of 3.24 minutes, 3.22 min actual

MSMS Scan at 3.259 minutes

Matching M/Zs above 1E-3*max: 134.037, 135.045, 179.035

All Matching M/Zs: 134.037, 135.045, 179.035

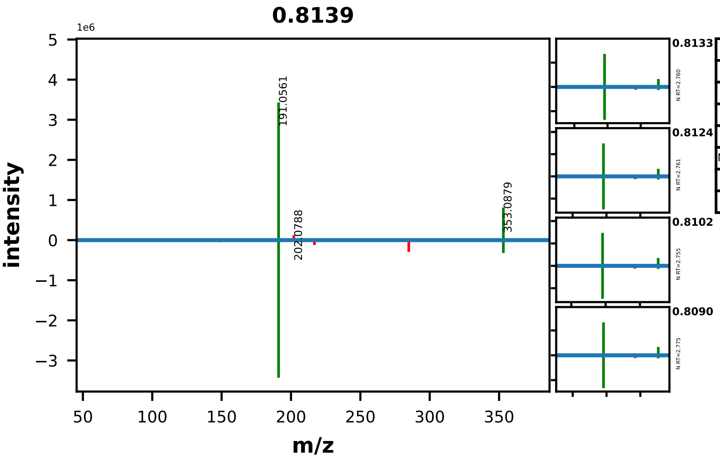

	EMA Compound Info
Name:	chlorogenic acid
Label:	
Formula:	C16H18O9
Polarity:	negative
Monoisotopic Mass:	354.095082156
Theoretical M/Z:	353.0877822
Adduct:	[M-H]-

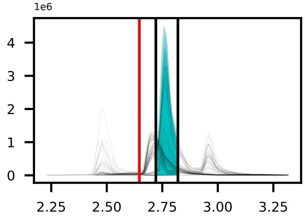

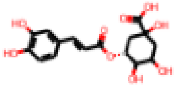

20221014_JGI_AE_507651_Poplar_final_IDX_C18_USDAY63675_NEG_MSMS_24_WT-Pholem-whole_A_Rg80to1200-CE102040--S1_Run68.h5

0050_chlorogenic_acid_negative_M-H353p0878_2p65 [M-H]-
Measured M/Z = 353.0875, 0.8694 ppm difference
Expected Elution of 2.65 minutes, 2.76 min actual

MSMS Scan at 2.761 minutes

Matching M/Zs above 1E-3*max: 161.024, 191.056, 353.088

All Matching M/Zs: 161.024, 191.056, 353.088

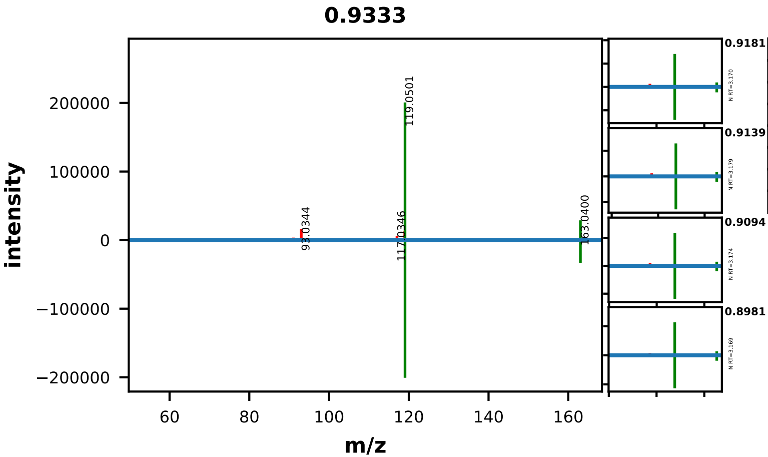

	EMA Compound Info
Name:	4-coumarate
Label:	
Formula:	C9H8O3
Polarity:	negative
Monoisotopic Mass:	164.047344116
Theoretical M/Z:	163.0400441
Adduct:	[M-H]-

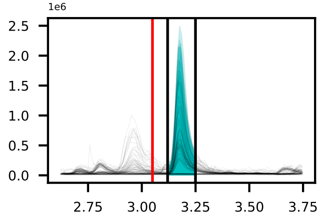

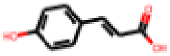

20221014_JGI_AE_507651_Poplar_final_IDX_C18_USDAY63675
_NEG_MSMS_117_Qsub5-Xylem-middle_D_Rg80to1200-CE205060
--S1_Run274.h5

0021_4-coumarate_negative_M-H163p0400_3p05 [M-H]-
Measured M/Z = 163.0401, 0.2951 ppm difference
Expected Elution of 3.05 minutes, 3.17 min actual

MSMS Scan at 3.174 minutes

Matching M/Zs above 1E-3*max: 119.050, 163.040

All Matching M/Zs: 119.050, 163.040

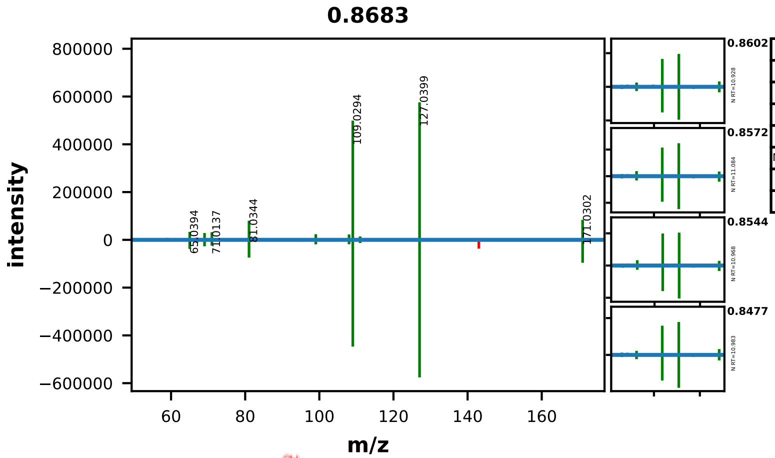

	EMA Compound Info
Name:	3-dehydroshikimic acid
Label:	
Formula:	C7H8O5
Polarity:	negative
Monoisotopic Mass:	172.037173356
Theoretical M/Z:	171.0298734
Adduct:	[M-H]-

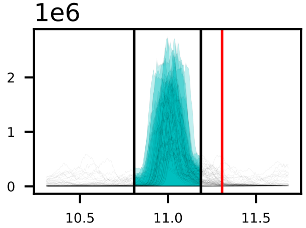

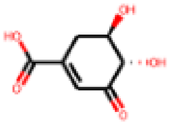

20220926_JGI_AE_507651_Poplar_final_QE-HF_HILICZ_USHXG01885_NEG_MSMS_24_WT-Pholem-whole_A_Rg70to1050-CE102040--S1_Run66.h5

0063_3-dehydroshikimic_acid_negative_M-H171p0299_11p31 [M-H]-
Measured M/Z = 171.0300, 0.6087 ppm difference
Expected Elution of 11.31 minutes, 11.03 min actual

MSMS Scan at 11.025 minutes

Matching M/Zs above 1E-3*max: 59.014, 65.039, 67.019, 69.034, 71.014, 71.050, 81.034, 83.050, 85.029, 92.426, 95.014, 97.030, 99.045, 108.022, 109.029, 111.009, 113.024, 127.040, 171.030

All Matching M/Zs: 59.014, 65.039, 67.019, 69.034, 71.014, 71.050, 81.034, 83.050, 85.029, 92.426, 95.014, 97.030, 99.045, 108.022, 109.029, 111.009, 113.024, 127.040, 171.030

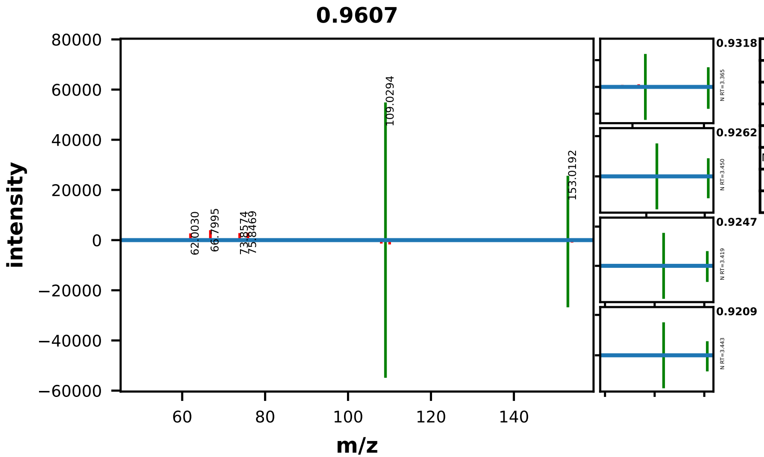

	EMA Compound Info
Name:	2,3-dihydroxybenzoic acid
Label:	
Formula:	C7H6O4
Polarity:	negative
Monoisotopic Mass:	154.026608672
Theoretical M/Z:	153.0193087
Adduct:	[M-H]-

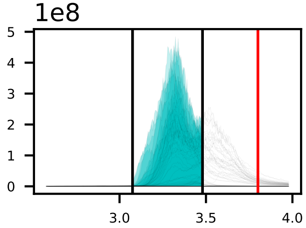

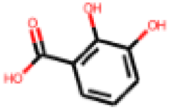

20220926_JGI_AE_507651_Poplar_final_QE-HF_HILICZ_USHXG01885_NEG_MSMS_164_ExCtrl_D_Rg70to1050-CE205060--S1_Run244.h5

0027_2_3-dihydroxybenzoic_acid_negative_M-H153p0193_3p80 [M-H]-
Measured M/Z = 153.0191, 1.3913 ppm difference
Expected Elution of 3.80 minutes, 3.08 min actual

MSMS Scan at 3.352 minutes

Matching M/Zs above 1E-3*max: 109.029, 153.019

All Matching M/Zs: 109.029, 153.019

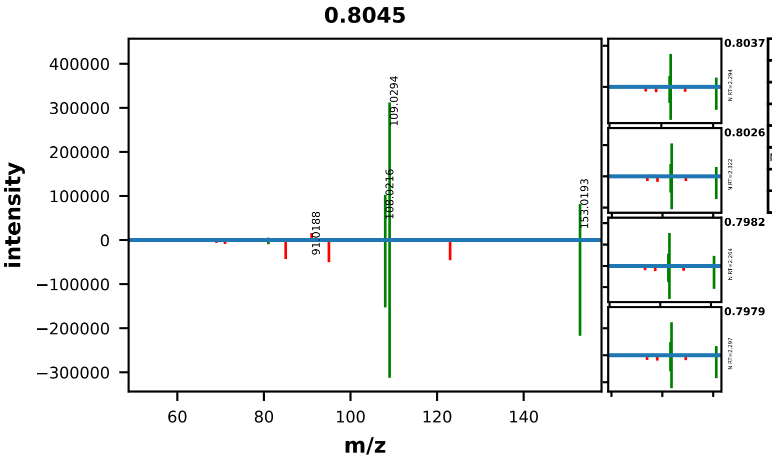

	EMA Compound Info
Name:	2,5-dihydroxybenzoate
Label:	
Formula:	C7H6O4
Polarity:	negative
Monoisotopic Mass:	154.026608672
Theoretical M/Z:	153.01930869999995
Adduct:	[M-H]-

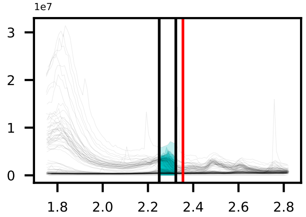

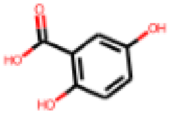

20221014_JGI_AE_507651_Poplar_final_IDX_C18_USDAY63675_NEG_MSMS_78_Qsub14-Xylem-bottom_D_Rg80to1200-CE205060--S1_Run239.h5

0049_2_5-dihydroxybenzoate_negative_M-H153p0193_2p35 [M-H]-
Measured M/Z = 153.0194, 0.8604 ppm difference
Expected Elution of 2.35 minutes, 2.29 min actual

MSMS Scan at 2.253 minutes

Matching M/Zs above 1E-3*max: 81.034, 108.022, 109.029, 153.019

All Matching M/Zs: 81.034, 108.022, 109.029, 153.019

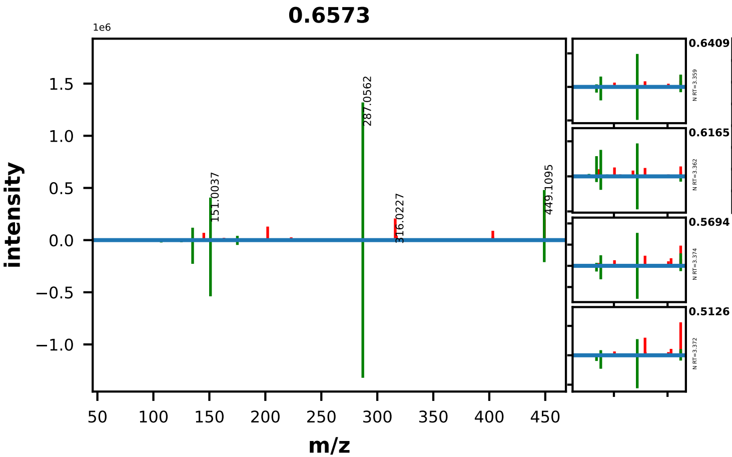

	EMA Compound Info
Name:	eriodictyol-7-o-glucoside
Label:	
Formula:	C21H22O11
Polarity:	negative
Monoisotopic Mass:	450.116211524
Theoretical M/Z:	449.1089115
Adduct:	[M-H]-

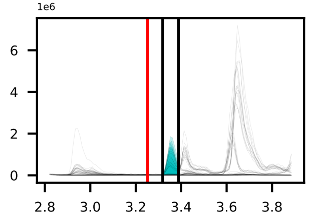

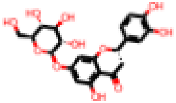

20221014_JGI_AE_507651_Poplar_final_IDX_C18_USDAY63675_NEG_MSMS_30_WT-Bark-whole_B_Rg80to1200-CE102040--S1_Run86.h5

0054_eriodictyol-7-o-glucoside_negative_M-H449p1089_3p25 [M-H]-
Measured M/Z = 449.1089, 0.0270 ppm difference
Expected Elution of 3.25 minutes, 3.35 min actual

MSMS Scan at 3.359 minutes

Matching M/Zs above 1E-3*max: 107.014, 109.030, 125.024, 135.045, 149.025, 151.004, 161.025, 169.014, 175.004, 193.014, 205.014, 269.046, 287.056, 311.056, 313.057, 329.067, 449.110

All Matching M/Zs: 107.014, 109.030, 125.024, 135.045, 149.025, 151.004, 161.025, 169.014, 175.004, 193.014, 205.014, 269.046, 287.056, 311.056, 313.057, 329.067, 449.110

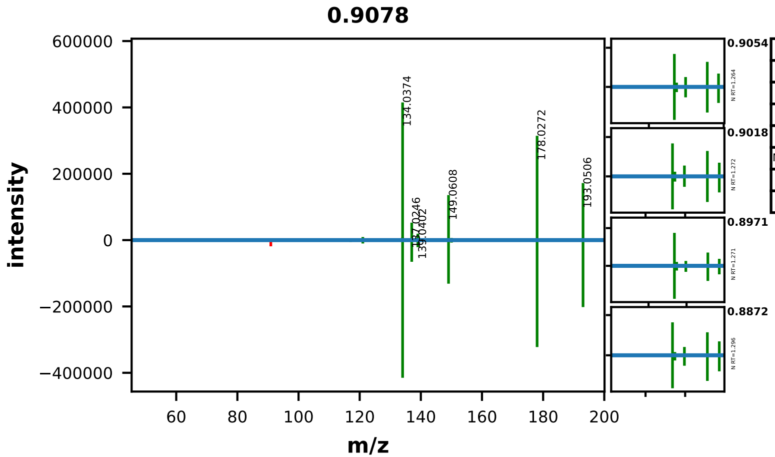

	EMA Compound Info
Name:	ferulic acid
Label:	
Formula:	C10H10O4
Polarity:	negative
Monoisotopic Mass:	194.0579088
Theoretical M/Z:	193.0506088
Adduct:	[M-H]-

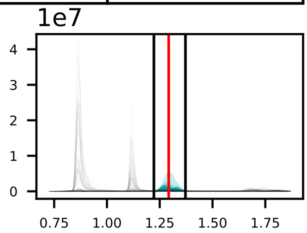

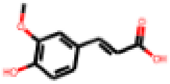

20220926_JGI_AE_507651_Poplar_final_QE-HF_HILICZ_USHXG01885_NEG_MSMS_111_Qsub5-Xylem-top_C_Rg70to1050-CE102040--S1_Run179.h5

0001_ferulic_acid_negative_M-H193p0506_1p29 [M-H]-
Measured M/Z = 193.0506, 0.1171 ppm difference
Expected Elution of 1.29 minutes, 1.28 min actual

MSMS Scan at 1.267 minutes

Matching M/Zs above 1E-3*max: 117.034, 121.030, 134.037, 137.025, 139.040, 149.061, 150.033, 178.027, 193.051

All Matching M/Zs: 117.034, 121.030, 134.037, 137.025, 139.040, 149.061, 150.033, 178.027, 193.051

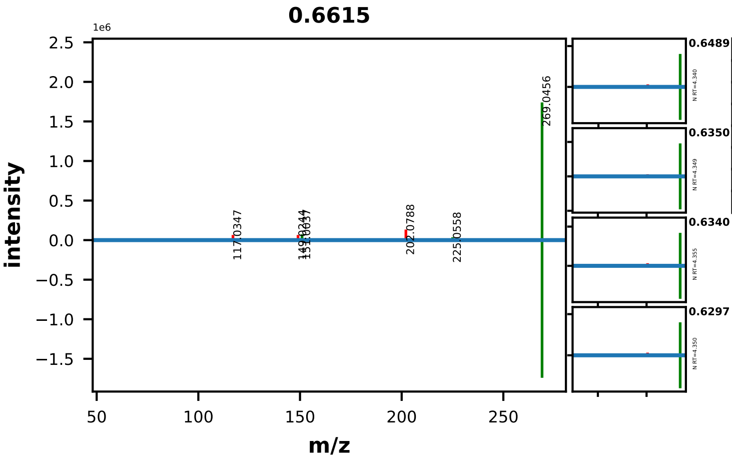

	EMA Compound Info
Name:	genistein, 4',5,7-trihydroxyisoflavone,
Label:	
Formula:	C15H10O5
Polarity:	negative
Monoisotopic Mass:	270.05282342
Theoretical M/Z:	269.0455234
Adduct:	[M-H]-

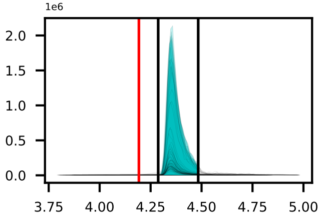

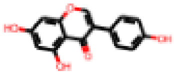

20221014_JGI_AE_507651_Poplar_final_IDX_C18_USDAY63675
_NEG_MSMS_115_Qsub5-Bark-whole_C_Rg80to1200-CE102040--
S1_Run159.h5

0009_genistein_4_5_7-trihydroxyisoflavone_negative_M-H269p0455_4p19 [M-[illegible]
Measured M/Z = 269.0455, 0.0443 ppm difference
Expected Elution of 4.19 minutes, 4.36 min actual

MSMS Scan at 4.349 minutes

Matching M/Zs above 1E-3*max: 65.003, 107.014, 151.004, 159.045, 181.066, 183.045, 197.061, 201.056, 224.048, 225.056, 227.035, 241.051, 269.046

All Matching M/Zs: 65.003, 107.014, 151.004, 159.045, 181.066, 183.045, 197.061, 201.056, 224.048, 225.056, 227.035, 241.051, 269.046

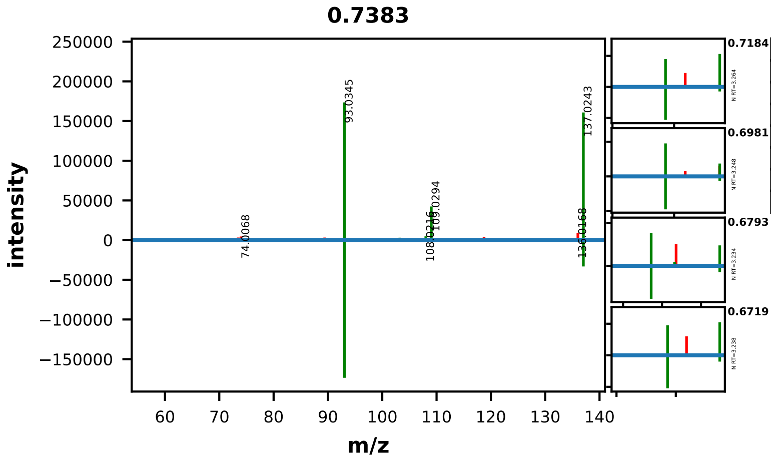

	EMA Compound Info
Name:	3-hydroxybenzoic acid
Label:	
Formula:	C7H6O3
Polarity:	negative
Monoisotopic Mass:	138.031694052
Theoretical M/Z:	137.0243941
Adduct:	[M-H]-

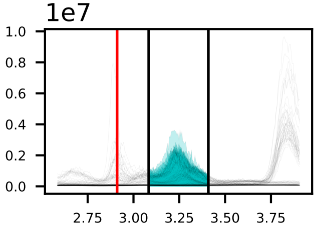

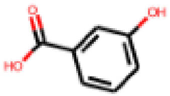

20220926_JGI_AE_507651_Poplar_final_QE-HF_HILICZ_USHXG01885_NEG_MSMS_75_Qsub1-Bark-whole_C_Rg70to1050-CE102040--S1_Run188.h5

0021_3-hydroxybenzoic_acid_negative_M-H137p0244_2p91 [M-H]-
Measured M/Z = 137.0244, 0.4031 ppm difference
Expected Elution of 2.91 minutes, 3.26 min actual

MSMS Scan at 3.286 minutes

Matching M/Zs above 1E-3*max: 93.035, 103.238, 108.022, 109.029, 137.024

All Matching M/Zs: 93.035, 103.238, 108.022, 109.029, 137.024

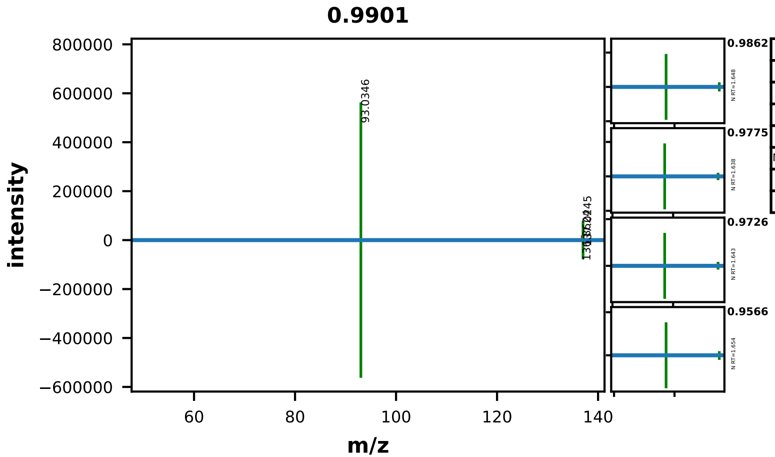

	EMA Compound Info
Name:	4-hydroxybenzoic acid
Label:	
Formula:	C7H6O3
Polarity:	negative
Monoisotopic Mass:	138.031694052
Theoretical M/Z:	137.0243941
Adduct:	[M-H]-

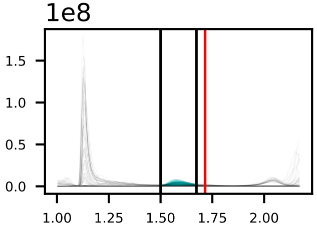

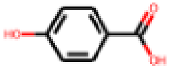

20220926_JGI_AE_507651_Poplar_final_QE-HF_HILICZ_USHXG01885_NEG_MSMS_72_Qsub1-Xylem-middle_C_Rg70to1050-CE102040--S1_Run170.h5

0011_4-hydroxybenzoic_acid_negative_M-H137p0244_1p71 [M-H]-
Measured M/Z = 137.0243, 0.3707 ppm difference
Expected Elution of 1.71 minutes, 1.59 min actual

MSMS Scan at 1.641 minutes

Matching M/Zs above 1E-3*max: 93.035, 137.025

All Matching M/Zs: 93.035, 137.025

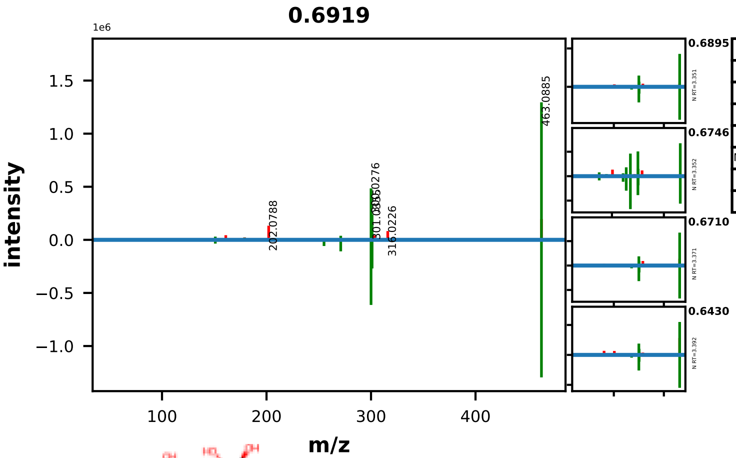

	EMA Compound Info
Name:	isoquercitrine
Label:	
Formula:	C21H20O12
Polarity:	negative
Monoisotopic Mass:	464.09547608
Theoretical M/Z:	463.0881761
Adduct:	[M-H]-

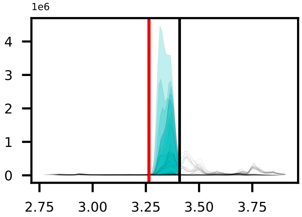

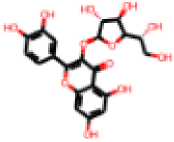

20221014_JGI_AE_507651_Poplar_final_IDX_C18_USDAY63675_NEG_MSMS_35_WT-Bark-whole_C_Rg80to1200-CE102040--S1_Run206.h5

0056_isoquercitrine_negative_M-H463p0882_3p27 [M-H]-
Measured M/Z = 463.0883, 0.2749 ppm difference
Expected Elution of 3.27 minutes, 3.37 min actual

MSMS Scan at 3.357 minutes

Matching M/Zs above 1E-3*max: 151.004, 178.999, 255.030, 271.025, 300.028, 301.035, 463.088

All Matching M/Zs: 151.004, 178.999, 255.030, 271.025, 300.028, 301.035, 463.088

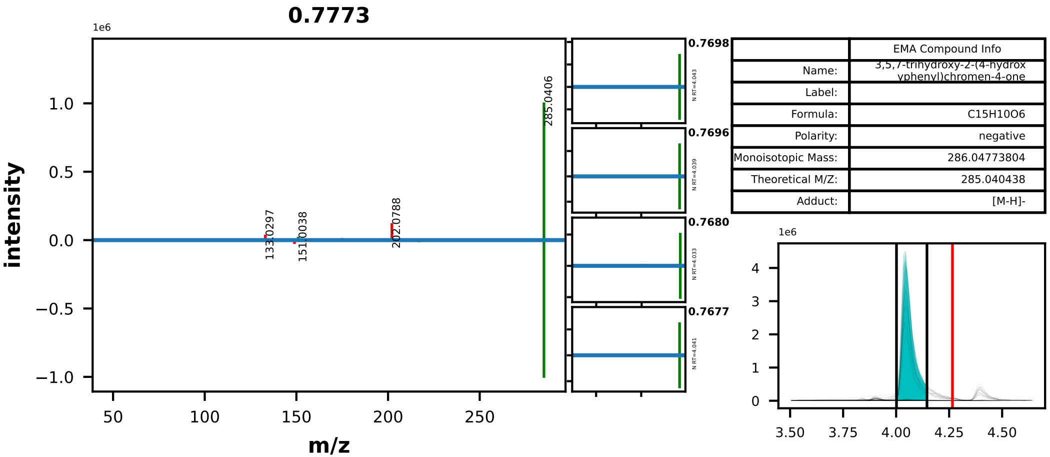

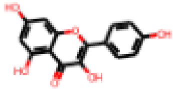

20221014_JGI_AE_507651_Poplar_final_IDX_C18_USDAY63675_NEG_MSMS_35_WT-Bark-whole_C_Rg80to1200-CE102040--S1_Run206.h5

0006_3_5_7-trihydroxy-24-hydroxyphenyl_chromen-4-one_negative_M-H285p0
Measured M/Z = 285.0404, 0.0195 ppm difference
Expected Elution of 4.27 minutes, 4.05 min actual

MSMS Scan at 4.094 minutes

Matching M/Zs above 1E-3*max: 151.004, 213.056, 285.041

All Matching M/Zs: 151.004, 213.056, 285.041

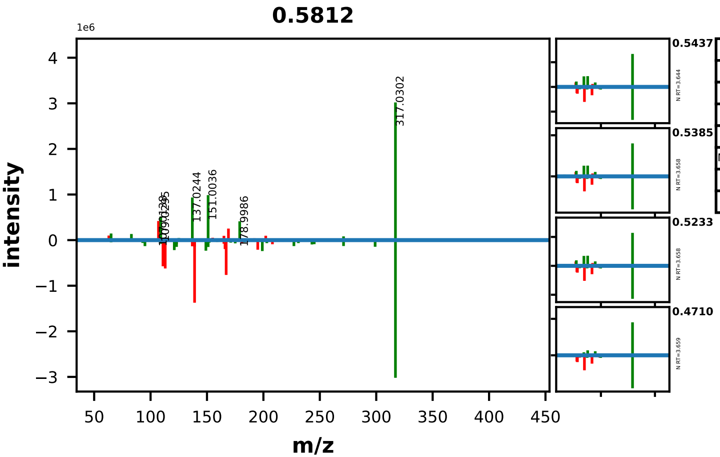

	EMA Compound Info
Name:	gossypetin
Label:	
Formula:	C15H10O8
Polarity:	negative
Monoisotopic Mass:	318.03756728
Theoretical M/Z:	317.0302673
Adduct:	[M-H]-

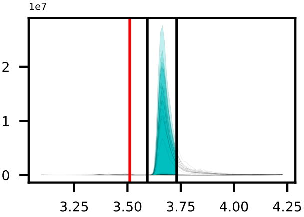

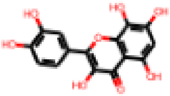

20221014_JGI_AE_507651_Poplar_final_IDX_C18_USDAY63675_NEG_MSMS_160_Qsub15-Bark-whole_D_Rg80to1200-CE205060--S1_Run245.h5

0063_gossypetin_negative_M-H317p0303_3p51 [M-H]-
Measured M/Z = 317.0302, 0.3534 ppm difference
Expected Elution of 3.51 minutes, 3.66 min actual

MSMS Scan at 3.656 minutes

Matching M/Zs above 1E-3*max: 65.003, 83.014, 93.035, 95.014, 108.022, 108.993, 109.029, 121.030, 123.009, 124.017, 125.024, 135.009, 136.017, 137.024, 143.050, 147.009, 147.045, 149.024, 151.004, 151.040, 152.011, 155.051, 157.029, 159.045, 161.025, 163.004, 164.012, 171.045, 173.061, 174.032, 175.040, 177.019, 178.999, 183.045, 185.024, 187.041, 189.019, 189.056, 191.035, 199.040, 201.020, 203.035, 215.036, 217.014, 217.051, 219.030, 227.035, 230.022, 231.030, 243.030, 245.045, 255.030, 261.040, 271.025, 273.040, 289.036, 299.020, 317.030

[illegible]

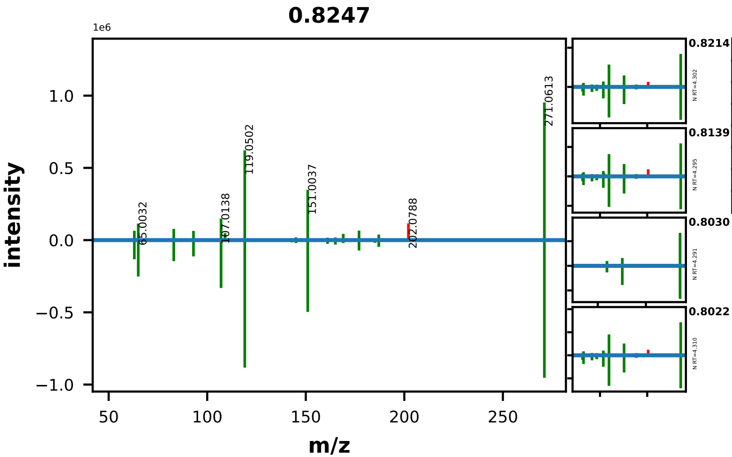

	EMA Compound Info
Name:	5,7-dihydroxy-2-(4-hydroxyphenyl)chroman-4-one
Label:	
Formula:	C15H12O5
Polarity:	negative
Monoisotopic Mass:	272.068473484
Theoretical M/Z:	271.0611735
Adduct:	[M-H]-

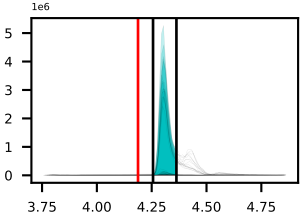

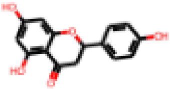

20221014_JGI_AE_507651_Poplar_final_IDX_C18_USDAY63675_NEG_MSMS_80_Qsub14-Bark-whole_D_Rg80to1200-CE205060--S1_Run215.h5

0002_5_7-dihydroxy-24-hydroxyphenyl_chroman-4-one_negative_M-H271p061
Measured M/Z = 271.0612, 0.0396 ppm difference
Expected Elution of 4.19 minutes, 4.30 min actual

MSMS Scan at 4.296 minutes

Matching M/Zs above 1E-3*max: 63.024, 65.003, 68.998, 81.034, 83.014, 93.034, 95.014, 107.014, 108.022, 108.993, 109.030, 117.035, 119.050, 121.030, 125.024, 133.030, 137.025, 143.050, 145.029, 151.004, 157.066, 161.061, 165.019, 169.014, 177.019, 185.061, 187.040, 227.072, 229.050, 271.061

All Matching M/Zs: 63.024, 65.003, 68.998, 81.034, 83.014, 93.034, 95.014, 107.014, 108.022, 108.993, 109.030, 117.035, 119.050, 121.030, 125.024, 133.030, 137.025, 143.050, 145.029, 151.004, 157.066, 161.061, 165.019, 169.014, 177.019, 185.061, 187.040, 227.072, [illegible]

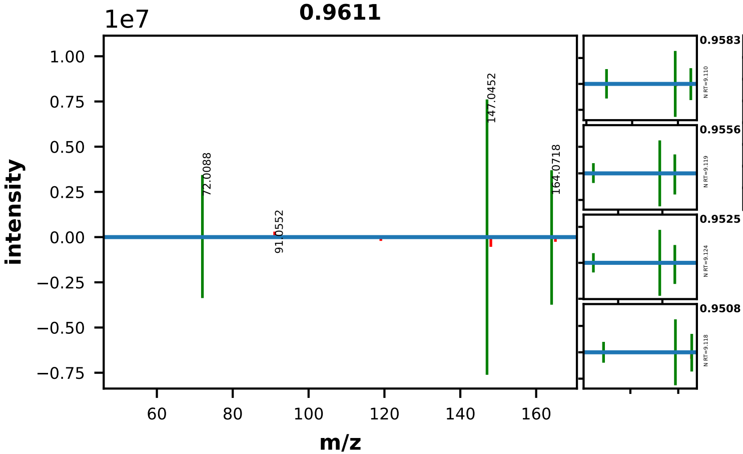

	EMA Compound Info
Name:	phenylalanine
Label:	
Formula:	C9H11NO2
Polarity:	negative
Monoisotopic Mass:	165.078978592
Theoretical M/Z:	164.0716786
Adduct:	[M-H]-

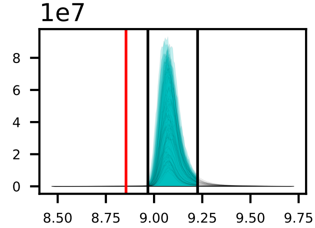

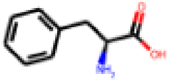

20220926_JGI_AE_507651_Poplar_final_QE-HF_HILICZ_USHXG01885_NEG_MSMS_157_Qsub15-Xylem-middle_D_Rg70to1050-CE205060--S1_Run237.h5

0046_phenylalanine_negative_M-H164p0717_8p85 [M-H]-
Measured M/Z = 164.0717, 0.0183 ppm difference
Expected Elution of 8.85 minutes, 9.07 min actual

MSMS Scan at 9.104 minutes

Matching M/Zs above 1E-3*max: 72.009, 147.045, 164.072

All Matching M/Zs: 72.009, 147.045, 164.072

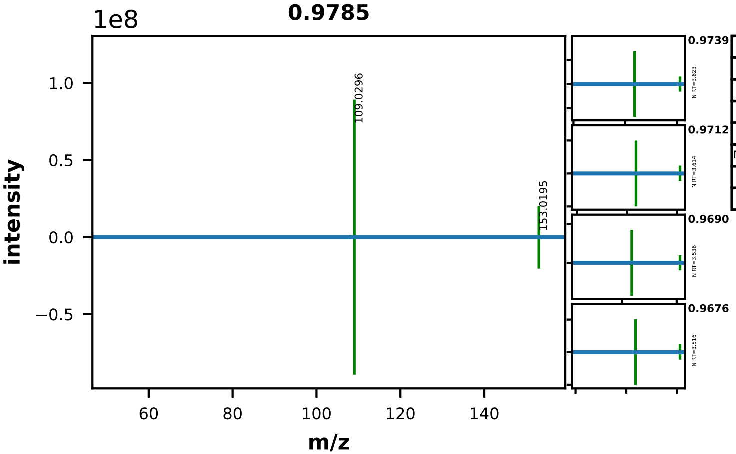

	EMA Compound Info
Name:	3,4-dihydroxybenzoic acid
Label:	
Formula:	C7H6O4
Polarity:	negative
Monoisotopic Mass:	154.026608672
Theoretical M/Z:	153.0193087
Adduct:	[M-H]-

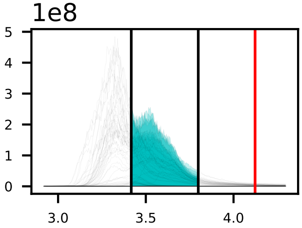

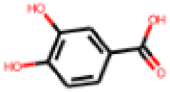

20220926_JGI_AE_507651_Poplar_final_QE-HF_HILICZ_USHXG01885_NEG_MSMS_73_Qsub1-Xylem-bottom_C_Rg70to1050-CE102040--S1_Run182.h5

0028_3_4-dihydroxybenzoic_acid_negative_M-H153p0193_4p12 [M-H]-
Measured M/Z = 153.0193, 0.1976 ppm difference
Expected Elution of 4.12 minutes, 3.49 min actual

MSMS Scan at 3.645 minutes

Matching M/Zs above 1E-3*max: 81.034, 108.022, 109.030, 153.019

All Matching M/Zs: 81.034, 108.022, 109.030, 153.019

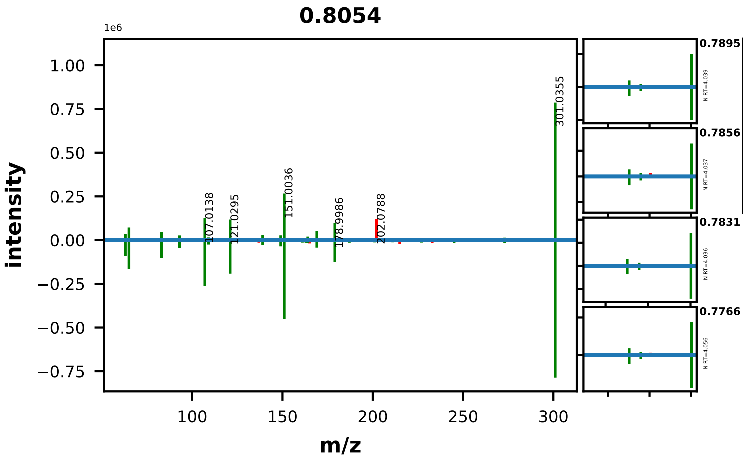

	EMA Compound Info
Name:	2-(3,4-dihydroxyphenyl)-3,5,<br>7-trihydroxychromen-4-one
Label:	
Formula:	C15H10O7
Polarity:	negative
Monoisotopic Mass:	302.04265266
Theoretical M/Z:	301.0353527
Adduct:	[M-H]-

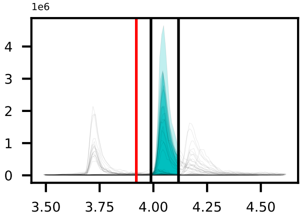

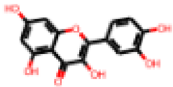

20221014_JGI_AE_507651_Poplar_final_IDX_C18_USDAY63675
_NEG_MSMS_40_WT-Bark-whole_D_Rg80to1200-CE205060--
S1_Run259.h5

0013_23_4-dihydroxyphenyl3_5_7-trihydroxychromen-4-one_negative_M-H301
Measured M/Z = 301.0353, 0.0176 ppm difference
Expected Elution of 3.92 minutes, 4.05 min actual

MSMS Scan at 4.036 minutes

Matching M/Zs above 1E-3*max: 63.024, 65.003, 83.014, 93.034, 95.014, 107.014, 108.993, 109.029, 121.029, 124.017, 125.025, 135.046, 139.040, 148.017, 149.024, 151.004, 152.012, 159.045, 161.024, 163.004, 163.040, 164.012, 169.014, 173.061, 175.041, 177.056, 178.999, 183.045, 187.040, 193.014, 201.056, 211.040, 227.035, 229.051, 243.030, 245.045, 255.031, 273.041, 301.036

All Matching M/Zs: 63.024, 65.003, 83.014, 93.034, 95.014, 107.014, 108.993, 109.029, 121.029, 124.017, 125.025, 135.046, 139.040, 148.017, 149.024, 151.004, 152.012, 159.045, [illegible]

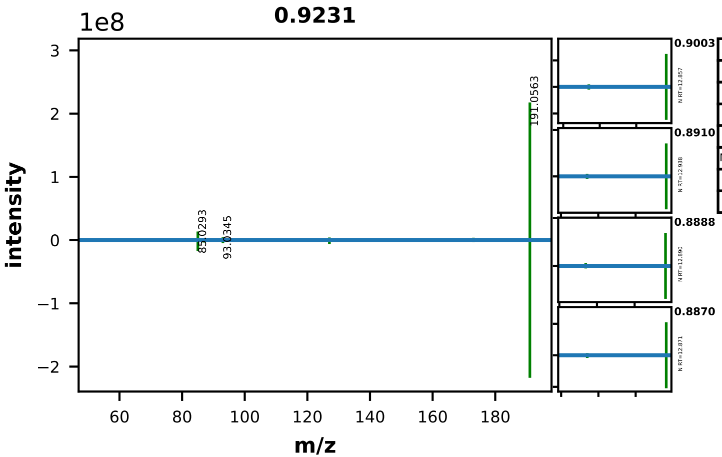

	EMA Compound Info
Name:	quinic acid
Label:	
Formula:	C7H12O6
Polarity:	negative
Monoisotopic Mass:	192.063388104
Theoretical M/Z:	191.0560881
Adduct:	[M-H]-

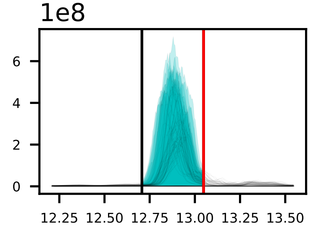

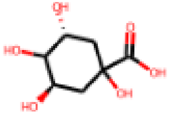

20220926_JGI_AE_507651_Poplar_final_QE-HF_HILICZ_USHXG01885_NEG_MSMS_21_WT-Xylem-top_A_Rg70to1050-CE102040--S1_Run75.h5

0068_quinic_acid_negative_M-H191p0561_13p05 [M-H]-
Measured M/Z = 191.0562, 0.3918 ppm difference
Expected Elution of 13.05 minutes, 12.90 min actual

MSMS Scan at 12.957 minutes

Matching M/Zs above 1E-3*max: 59.013, 71.013, 85.029, 87.009, 93.035, 99.045, 109.029, 111.045, 127.040, 171.030, 173.046, 191.056

All Matching M/Zs: 59.013, 71.013, 85.029, 87.009, 93.035, 99.045, 109.029, 111.045, 127.040, 171.030, 173.046, 191.056

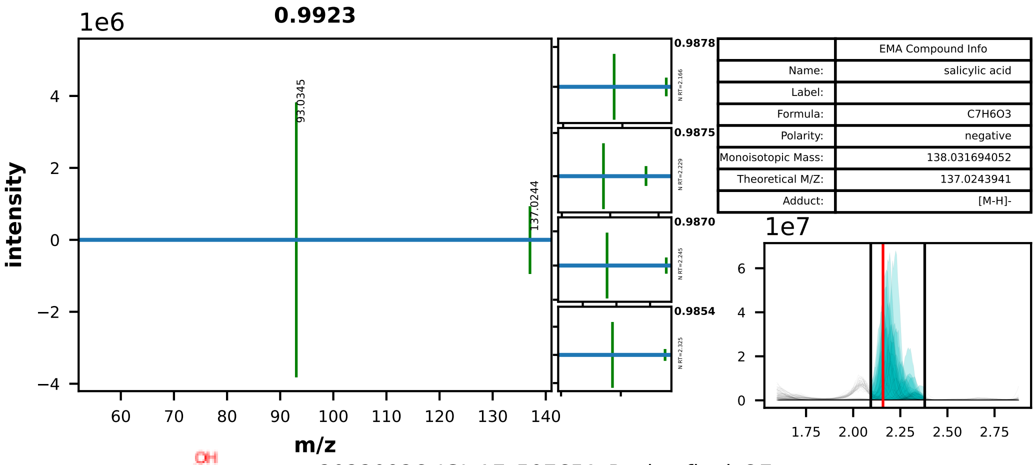

	EMA Compound Info
Name:	salicylic acid
Label:	
Formula:	C7H6O3
Polarity:	negative
Monoisotopic Mass:	138.031694052
Theoretical M/Z:	137.0243941
Adduct:	[M-H]-

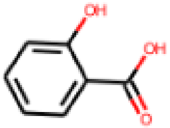

20220926_JGI_AE_507651_Poplar_final_QE-HF_HILICZ_USHXG01885_NEG_MSMS_70_Qsub1-Bark-whole_B_Rg70to1050-CE102040--S1_Run136.h5

0013_salicylic_acid_negative_M-H137p0244_2p16 [M-H]-
Measured M/Z = 137.0243, 0.3878 ppm difference
Expected Elution of 2.16 minutes, 2.18 min actual

MSMS Scan at 2.211 minutes

Matching M/Zs above 1E-3*max: 93.035, 137.024

All Matching M/Zs: 93.035, 137.024

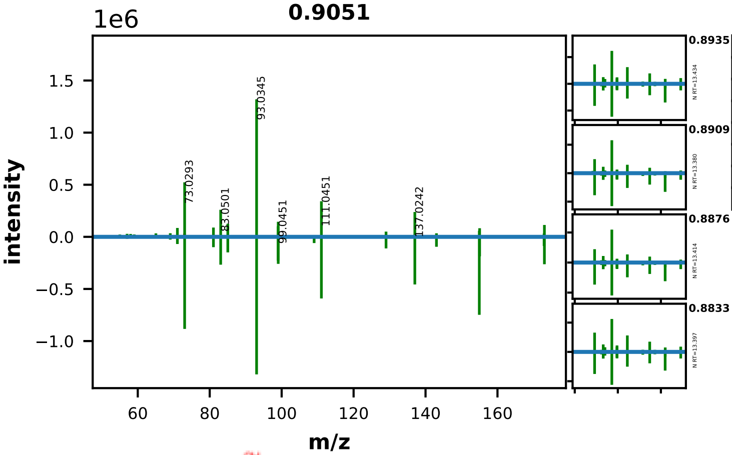

	EMA Compound Info
Name:	shikimic acid
Label:	
Formula:	C7H10O5
Polarity:	negative
Monoisotopic Mass:	174.05282342
Theoretical M/Z:	173.0455234
Adduct:	[M-H]-

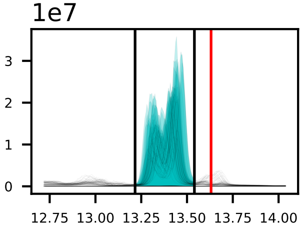

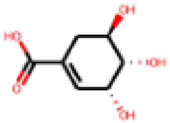

20220926_JGI_AE_507651_Poplar_final_QE-HF_HILICZ_USHXG01885_NEG_MSMS_120_Qsub5-Bark-whole_D_Rg70to1050-CE205060--S1_Run222.h5

0076_shikimic_acid_negative_M-H173p0455_13p63 [M-H]-
Measured M/Z = 173.0453, 1.4209 ppm difference
Expected Elution of 13.63 minutes, 13.41 min actual

MSMS Scan at 13.396 minutes

Matching M/Zs above 1E-3*max: 55.018, 57.034, 58.006, 59.013, 65.039, 69.034, 71.014, 73.029, 73.036, 81.034, 83.050, 85.029, 93.035, 99.009, 99.045, 101.024, 109.030, 111.045, 129.019, 137.024, 143.035, 154.947, 155.035, 172.957, 173.045

All Matching M/Zs: 55.018, 57.034, 58.006, 59.013, 65.039, 69.034, 71.014, 73.029, 73.036, 81.034, 83.050, 85.029, 93.035, 99.009, 99.045, 101.024, 109.030, 111.045, 129.019, 137.024, 143.035, 154.947, 155.035, 172.957, 173.045

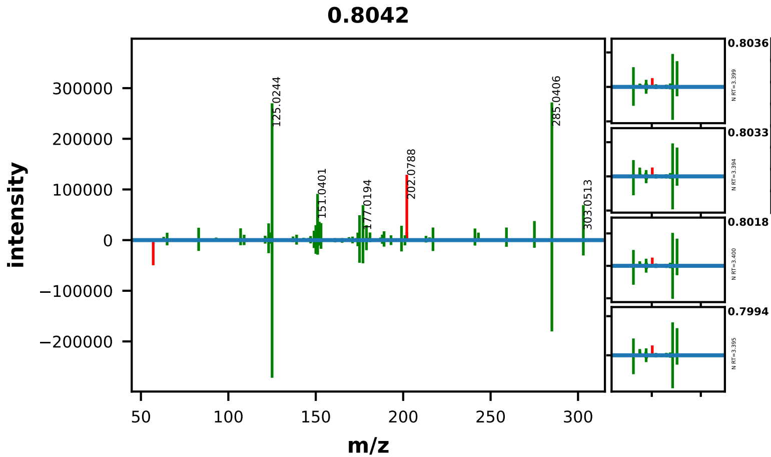

	EMA Compound Info
Name:	taxifolin
Label:	
Formula:	C15H12O7
Polarity:	negative
Monoisotopic Mass:	304.058302724
Theoretical M/Z:	303.0510027
Adduct:	[M-H]-

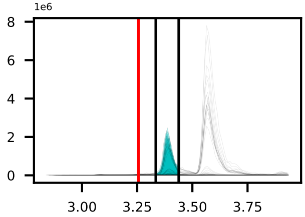

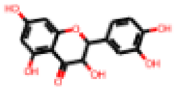

20221014_JGI_AE_507651_Poplar_final_IDX_C18_USDAY63675_NEG_MSMS_120_Qsub5-Bark-whole_D_Rg80to1200-CE205060--S1_Run227.h5

0039_taxifolin_negative_M-H303p0510_3p26 [M-H]-
Measured M/Z = 303.0510, 0.0993 ppm difference
Expected Elution of 3.26 minutes, 3.38 min actual

MSMS Scan at 3.395 minutes

Matching M/Zs above 1E-3*max: 63.024, 65.003, 81.035, 83.014, 93.035, 107.014, 108.022, 109.030, 119.050, 121.030, 122.037, 123.045, 124.017, 125.024, 133.030, 133.066, 137.024, 139.040, 143.051, 146.037, 147.045, 149.025, 150.032, 151.004, 151.040, 152.012, 153.019, 161.060, 164.048, 165.056, 169.014, 171.045, 172.053, 173.025, 174.032, 175.040, 176.012, 177.019, 178.999, 181.014, 185.062, 187.040, 188.048, 189.056, 193.051, 199.040, 201.020, 213.056, 215.035, 216.043, 217.051, 231.067, 241.051, 243.030, 259.061, 275.056, 285.041, 303.051

[illegible]

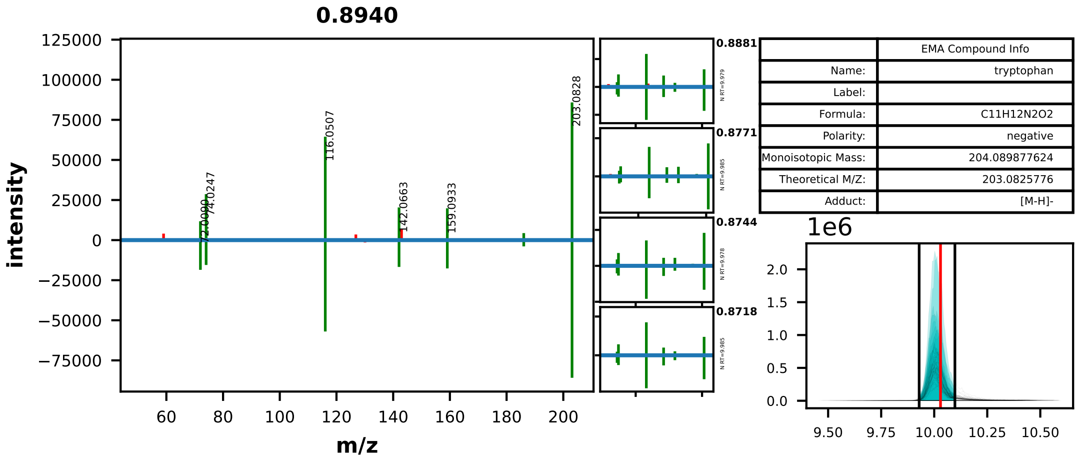

	EMA Compound Info
Name:	tryptophan
Label:	
Formula:	C11H12N2O2
Polarity:	negative
Monoisotopic Mass:	204.089877624
Theoretical M/Z:	203.0825776
Adduct:	[M-H]-

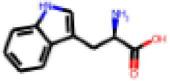

20220926_JGI_AE_507651_Poplar_final_QE-HF_HILICZ_USHXG01885_NEG_MSMS_29_WT-Pholem-whole_B_Rg70to1050-CE102040--S1_Run115.h5

0057_tryptophan_negative_M-H203p0826_10p03 [M-H]-
Measured M/Z = 203.0826, 0.2577 ppm difference
Expected Elution of 10.03 minutes, 10.00 min actual

MSMS Scan at 9.977 minutes

Matching M/Zs above 1E-3*max: 72.009, 74.025, 116.051, 142.066, 159.093, 186.056, 203.083

All Matching M/Zs: 72.009, 74.025, 116.051, 142.066, 159.093, 186.056, 203.083

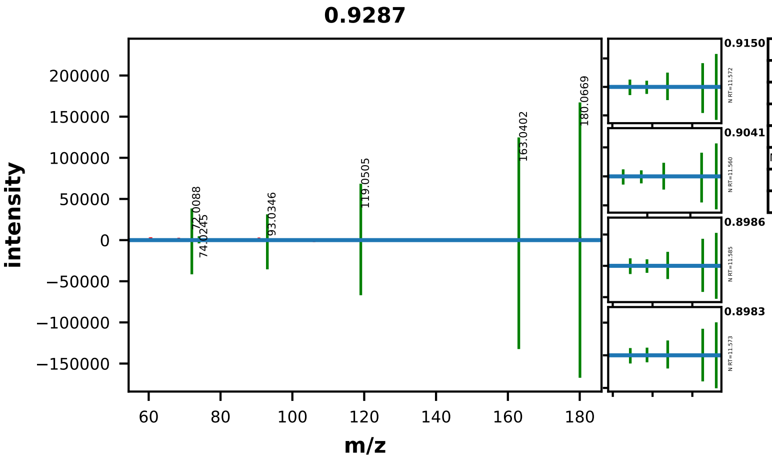

	EMA Compound Info
Name:	tyrosine
Label:	
Formula:	C9H11NO3
Polarity:	negative
Monoisotopic Mass:	181.073893212
Theoretical M/Z:	180.0665932
Adduct:	[M-H]-

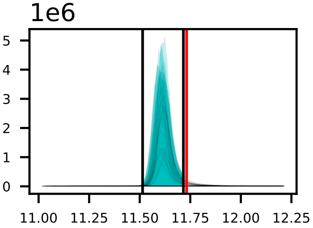

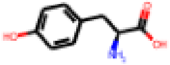

20220926_JGI_AE_507651_Poplar_final_QE-HF_HILICZ_USHXG01885_NEG_MSMS_64_Qsub1-Pholem-whole_A_Rg70to1050-CE102040--S1_Run50.h5

0066_tyrosine_negative_M-H180p0666_11p73 [M-H]-
Measured M/Z = 180.0667, 0.3673 ppm difference
Expected Elution of 11.73 minutes, 11.62 min actual

MSMS Scan at 11.607 minutes

Matching M/Zs above 1E-3*max: 72.009, 74.024, 93.035, 119.050, 163.040, 180.067

All Matching M/Zs: 72.009, 74.024, 93.035, 119.050, 163.040, 180.067

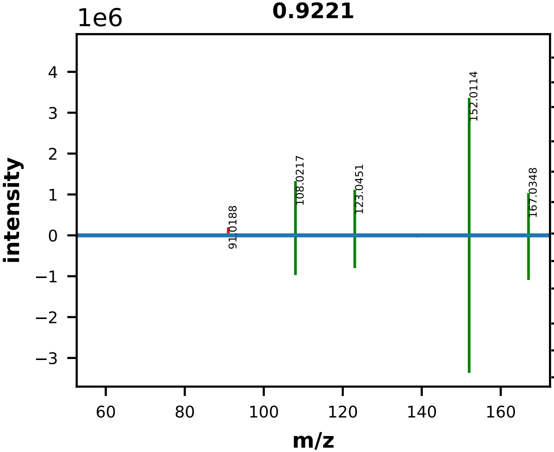

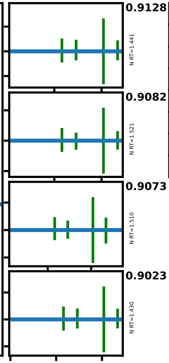

	EMA Compound Info
Name:	vanillic acid
Label:	
Formula:	C8H8O4
Polarity:	negative
Monoisotopic Mass:	168.042258736
Theoretical M/Z:	167.0349587
Adduct:	[M-H]-

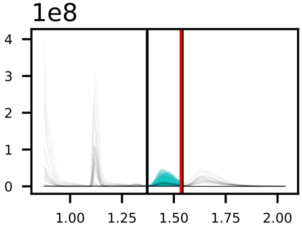

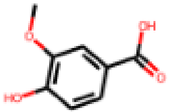

20220926_JGI_AE_507651_Poplar_final_QE-HF_HILICZ_USHXG01885_NEG_MSMS_153_Qsub15-Xylem-bottom_C_Rg70to1050-CE102040--S1_Run142.h5

0006_vanillic_acid_negative_M-H167p0350_1p53 [M-H]-
Measured M/Z = 167.0349, 0.4210 ppm difference
Expected Elution of 1.53 minutes, 1.45 min actual

MSMS Scan at 1.455 minutes

Matching M/Zs above 1E-3*max: 91.025, 108.022, 123.045, 152.011, 167.035

All Matching M/Zs: 91.025, 108.022, 123.045, 152.011, 167.035
