# Supplementary material for: Expression of dehydroshikimate dehydratase in poplar induces transcriptional and metabolic changes in the phenylpropanoid pathway
Source: J Exp Bot. 2024 May 29;75(16):4960–77. doi: 10.1093/jxb/erae251 (PMC11349870; doi:10.1093/jxb/erae251)
Supplement: erae251_suppl_Supplementary_Materials [file erae251_suppl_supplementary_materials.zip › erae251_suppl_Supplementary_Dataset_S8.pdf]

Heterologous expression in poplar of dehydroshikimate dehydratase induces transcriptional and metabolic changes in phenylpropanoid pathway

Emine Akyuz Turumtay[1,2,3], Halbay Turumtay[1,2,4], Yang Tian[1,2], Chien-Yuan Lin[1,2], Yen Ning Chai[1,2], Katherine B. Louie[2,5], Yan Chen[1,6], Anna Lipzen[5], Thomas Harwood[2,5], Kavitha Satish Kumar[1,2], Benjamin P. Bowen[2,5], Qian Wang[7,8], Shawn D. Mansfield[7,8,9], Matthew J. Blow[5], Christopher J. Petzold[1,6], Trent R. Northen[2,5], Jenny C. Mortimer[1,2,10], Henrik V. Scheller[1,2,11], Aymerick Eudes[1,2,*]

[1] Feedstocks Division, Joint BioEnergy Institute, Emeryville, CA, USA
[2] Environmental Genomics and Systems Biology Division, Lawrence Berkeley National Laboratory, Berkeley, CA, USA
[3] Recep Tayyip Erdogan University, Department of Chemistry, 53100, Rize, Turkiye
[4] Karadeniz Technical University, Department of Energy System Engineering, 61830, Trabzon, Turkiye
[5] Joint Genome Institute, Lawrence Berkeley National Laboratory, Berkeley, CA, United States
[6] Biological Systems & Engineering Division, Lawrence Berkeley National Laboratory, Berkeley, CA, USA
[7] Department of Wood Science, University of British Columbia, Vancouver, BC, Canada
[8] Department of Botany, University of British Columbia, Vancouver, BC, Canada
[9] DOE Great Lakes Bioenergy Research Center, Wisconsin Energy Institute, Madison, WI 53726, USA
[10] School of Agriculture, Food and Wine & Waite Research Institute, University of Adelaide, Glen Osmond, SA, Australia.
[11] Department of Plant and Microbial Biology, University of California, Berkeley, Berkeley, CA, USA

*Correspondence: Aymerick Eudes, ageudes@lbl.gov

Supplementary Dataset S8: Nontargeted metabolomic analysis using reverse phase C_{18} chromatography.
Number of figures: 8

C18 positive mode (17,023 unique features)

A

	WT	QsuB1	QsuB5	QsuB15
**Xylem bottom**	9,581	11,189	9,906	10,600
**Xylem middle**	9,991	11,526	10,252	11,193
**Xylem top**	10,290	12,318	10,811	11,553
**Phloem**	13,467	14,277	13,957	14,517
**Periderm**	13,600	14,363	14,082	14,407

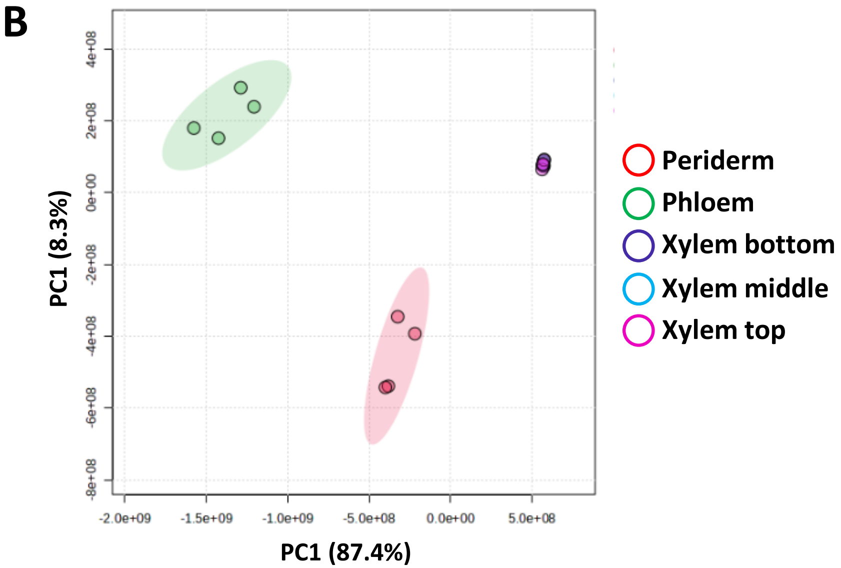

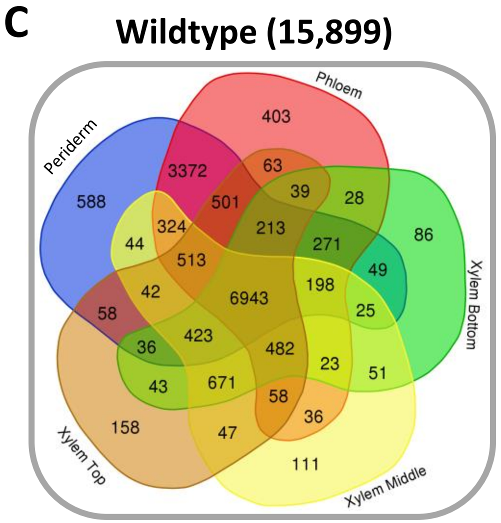

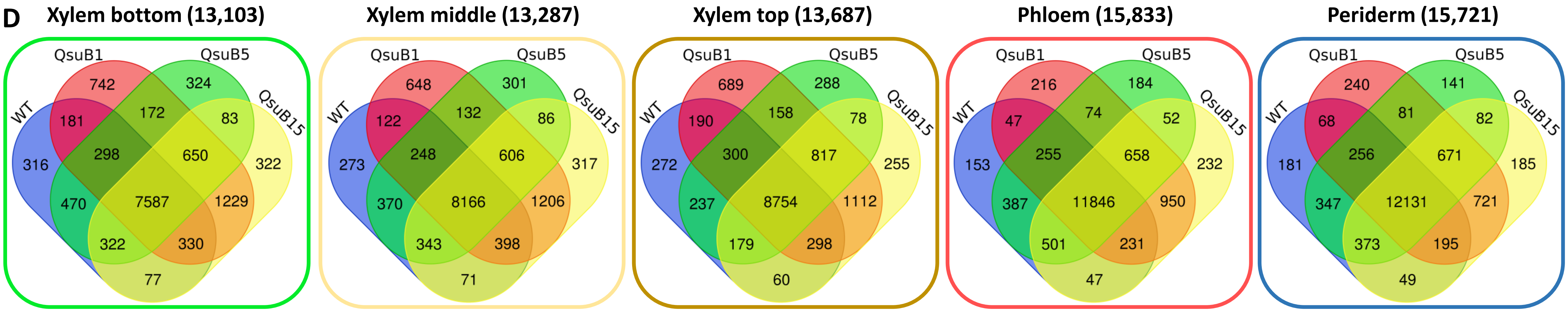

Dataset S8.1. Features detected in WT and transgenic QsuB poplar lines using C_{18} chromatography (positive ionization mode). Number of features detected in each tissue from the different lines (**A**). PCA plot (**B**) and venn diagram (**C**) of features detected in different tissues from WT stems. Venn diagram of features detected in WT and QsuB lines for each tissue (**D**). The number of unique features is indicated in brackets for each tissue.

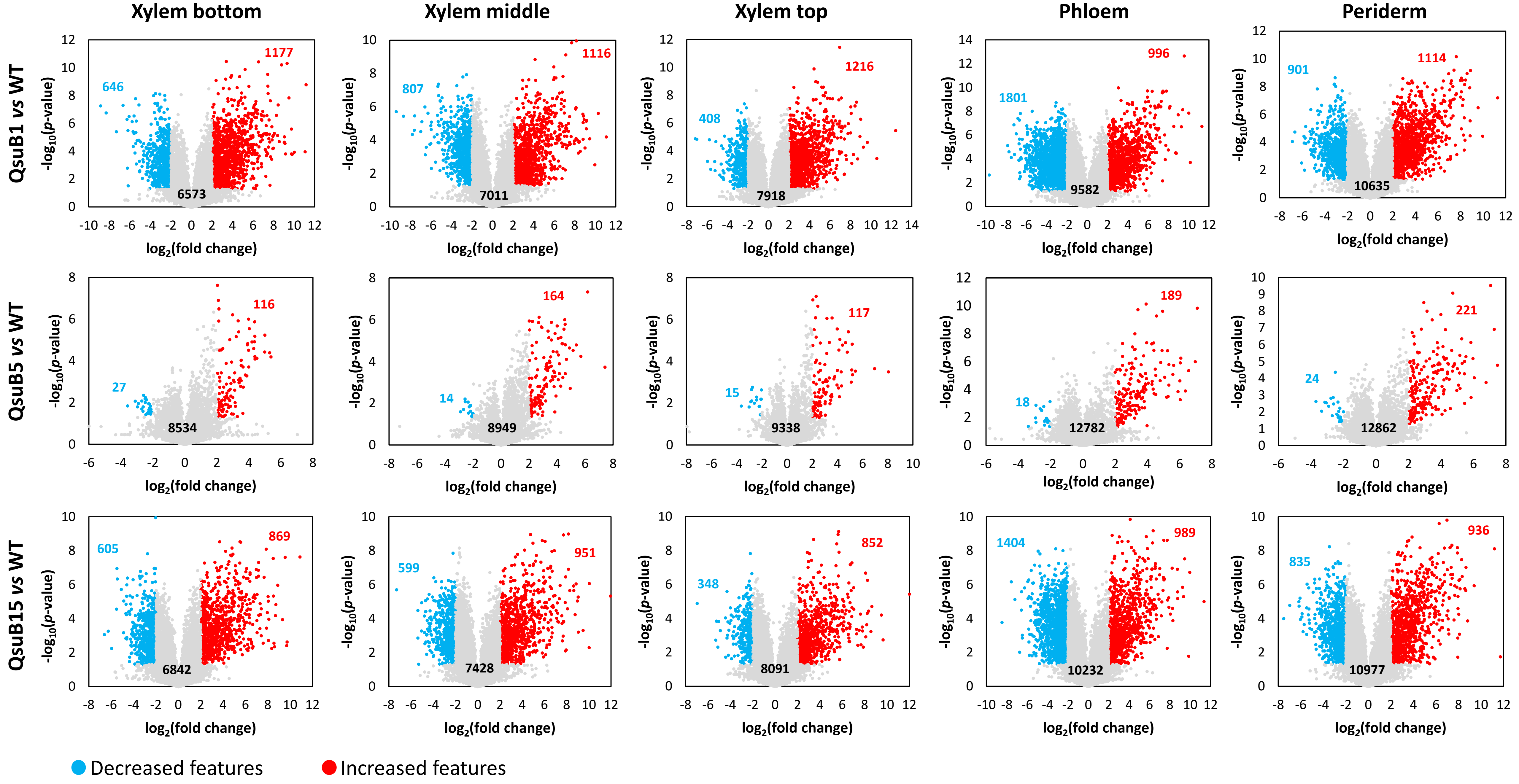

Dataset S8.2. Volcano plots of features detected in WT and QsuB transgenic lines in different stem tissues using C_{18} chromatography (positive ionization mode). Gray dots represent features not differentially abundant.

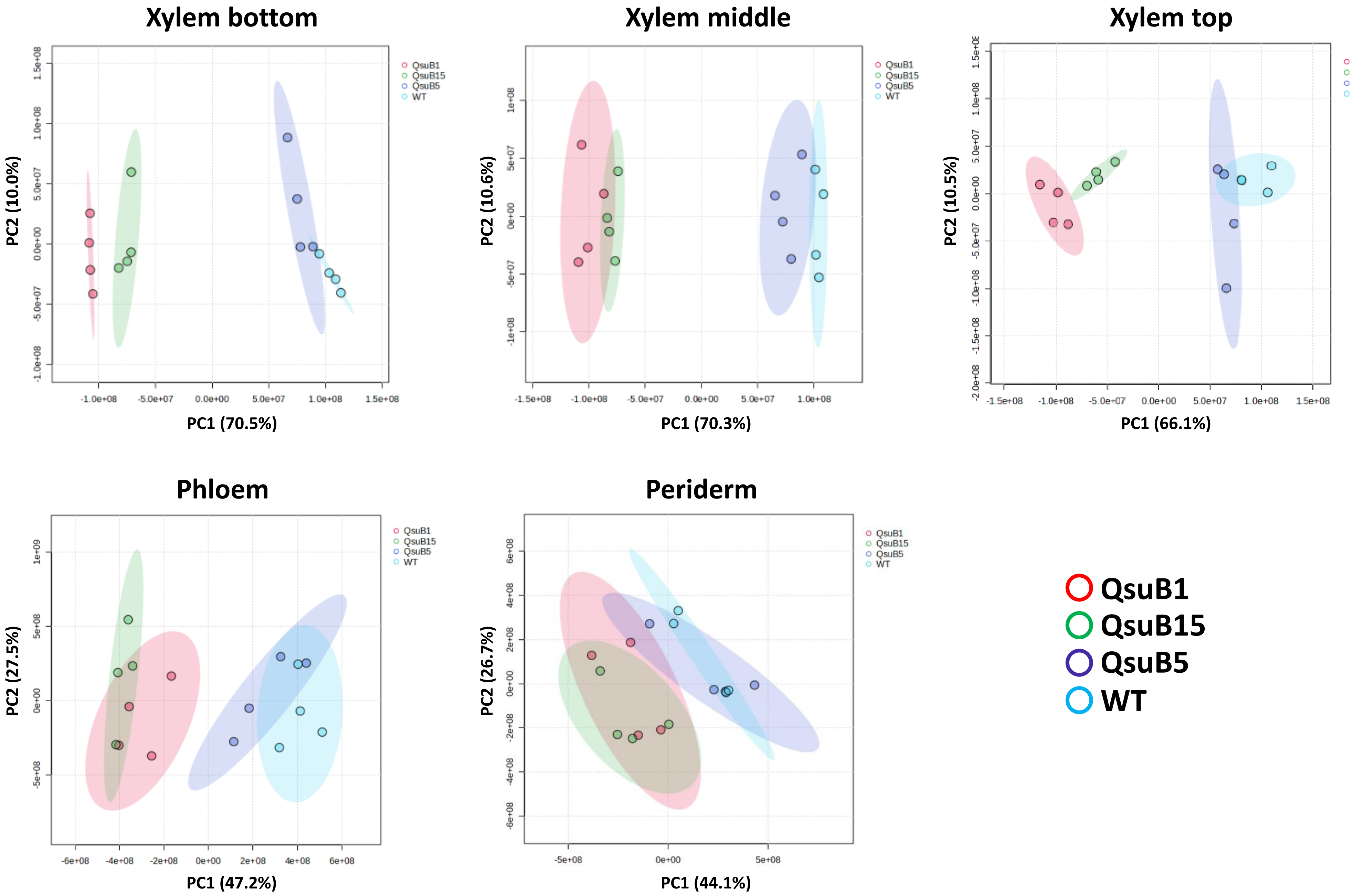

Dataset S8.3. PCA plots of the features detected in WT and QsuB lines in each tissue (C_{18} chromatography, positive ionization mode).

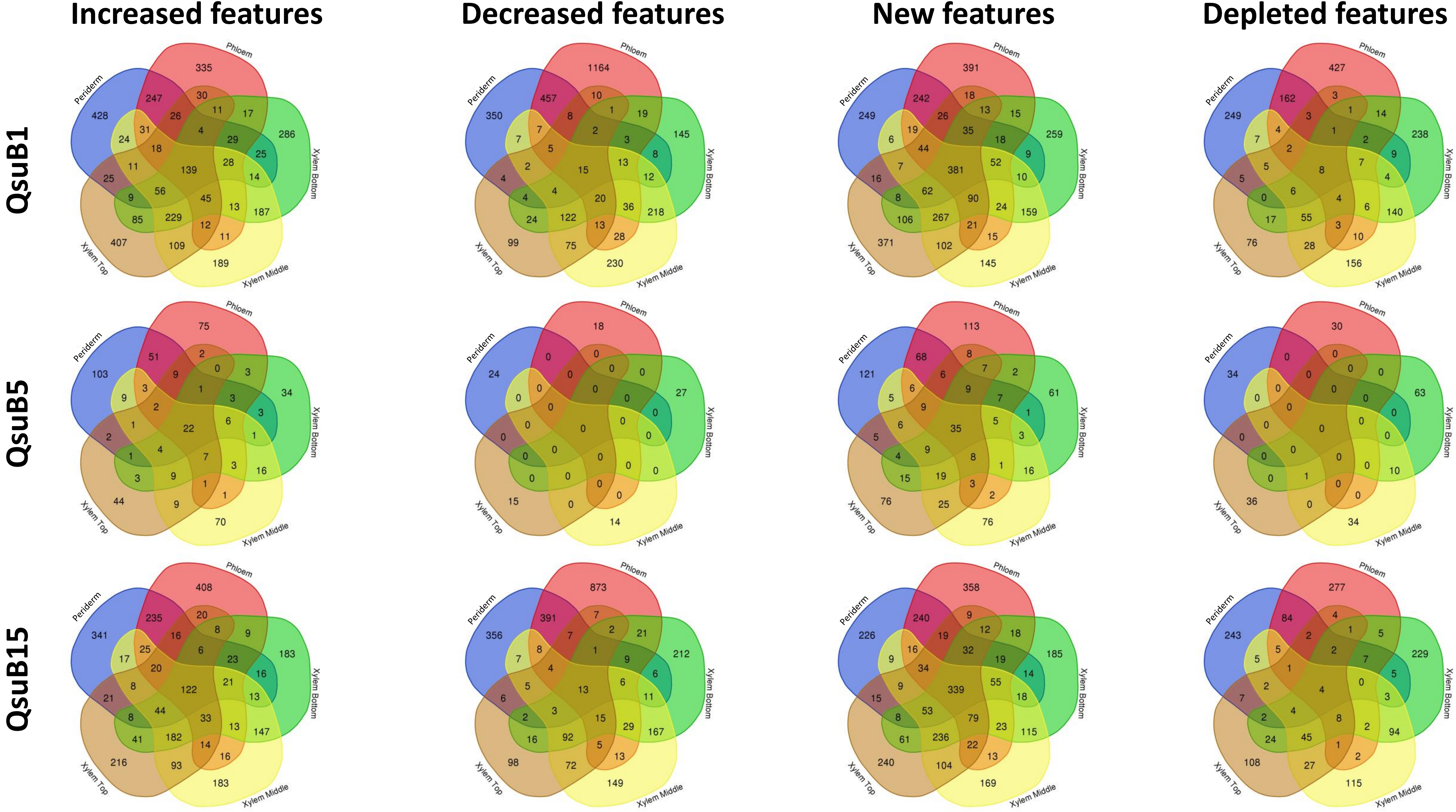

Dataset S8.4. Venn diagrams of features more abundant, less abundant, new, and depleted in different tissues of the QsuB lines (C_{18} positive ionization mode).

Dataset S8.5. Classification of differentially abundant metabolites identified in each tissue of the QsuB lines using C_{18} chromatography (positive ionization mode). For each class, the number of metabolites is indicated inside the corresponding slice of the pie chart. A breakdown of new and depleted metabolites (upper panels) and of increased and decreased metabolites (lower panels) is indicated next to each color class symbol.

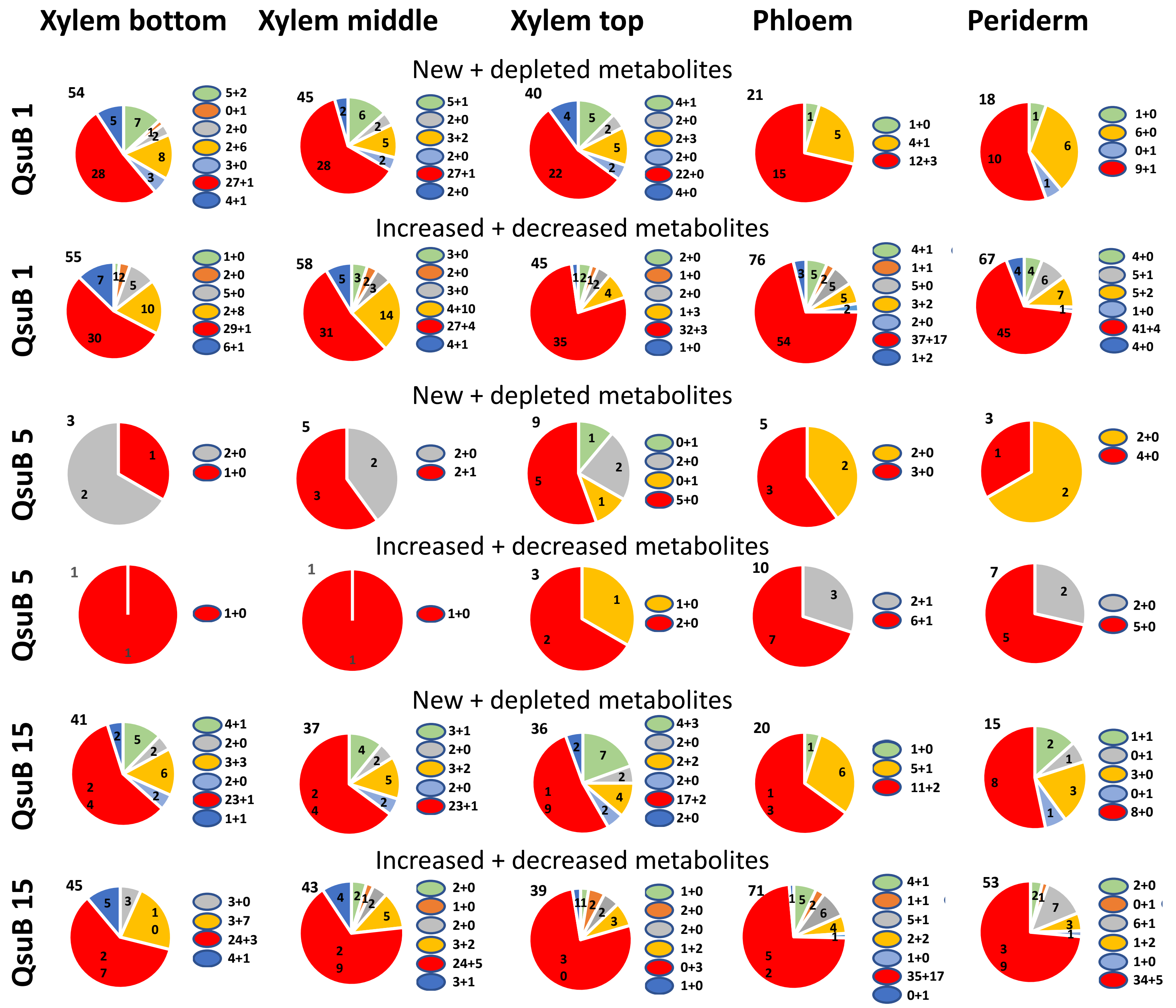

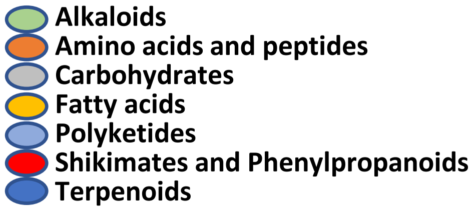

C18 negative mode (8,512 unique features)

A

	WT	QsuB1	QsuB5	QsuB15
**Xylem bottom**	4,941	5,931	5,044	5,629
**Xylem middle**	4,980	6,049	5,285	5,935
**Xylem top**	5,087	6,341	5,460	5,968
**Phloem**	6,825	7,492	7,202	7,551
**Periderm**	7,051	7,590	7,405	7,637

B **Wildtype (7,882)**

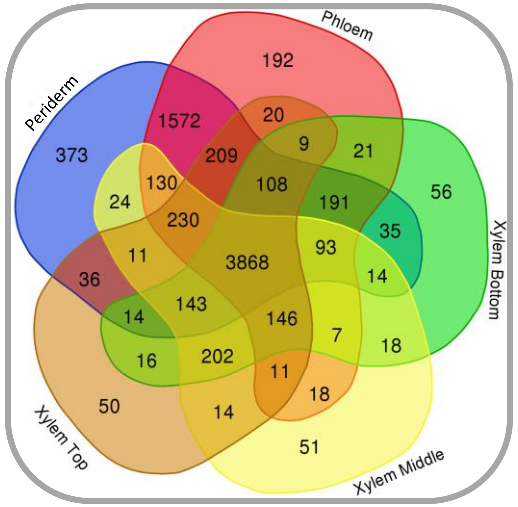

D

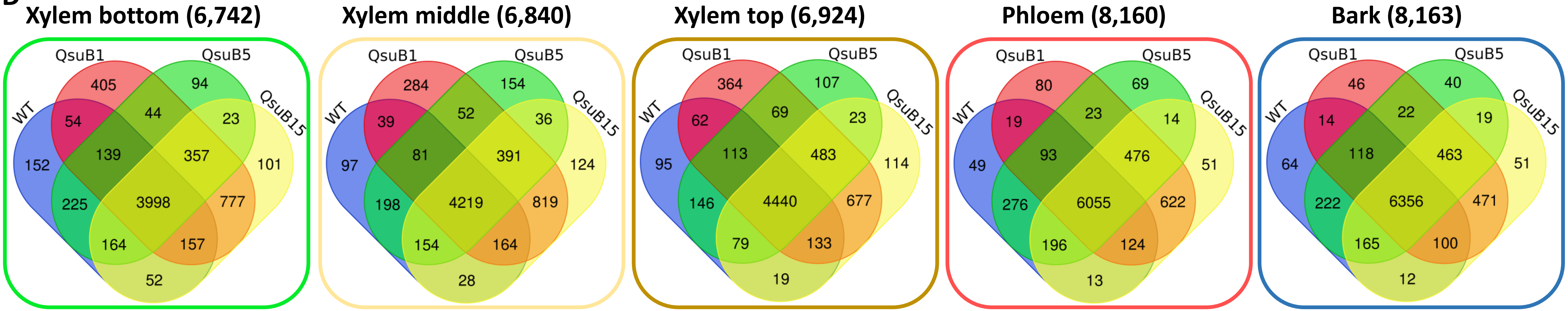

Dataset S8.6. Features detected in WT and transgenic QsuB poplar lines using C_{18} chromatography (negative ionization mode). Number of features detected in each tissue from the different lines (**A**) . Venn diagram of features detected in different tissues from WT stems (**B**). Venn diagram of features detected in WT and QsuB lines for each tissue (**C**). The number of unique features is indicated in brackets for each tissue.

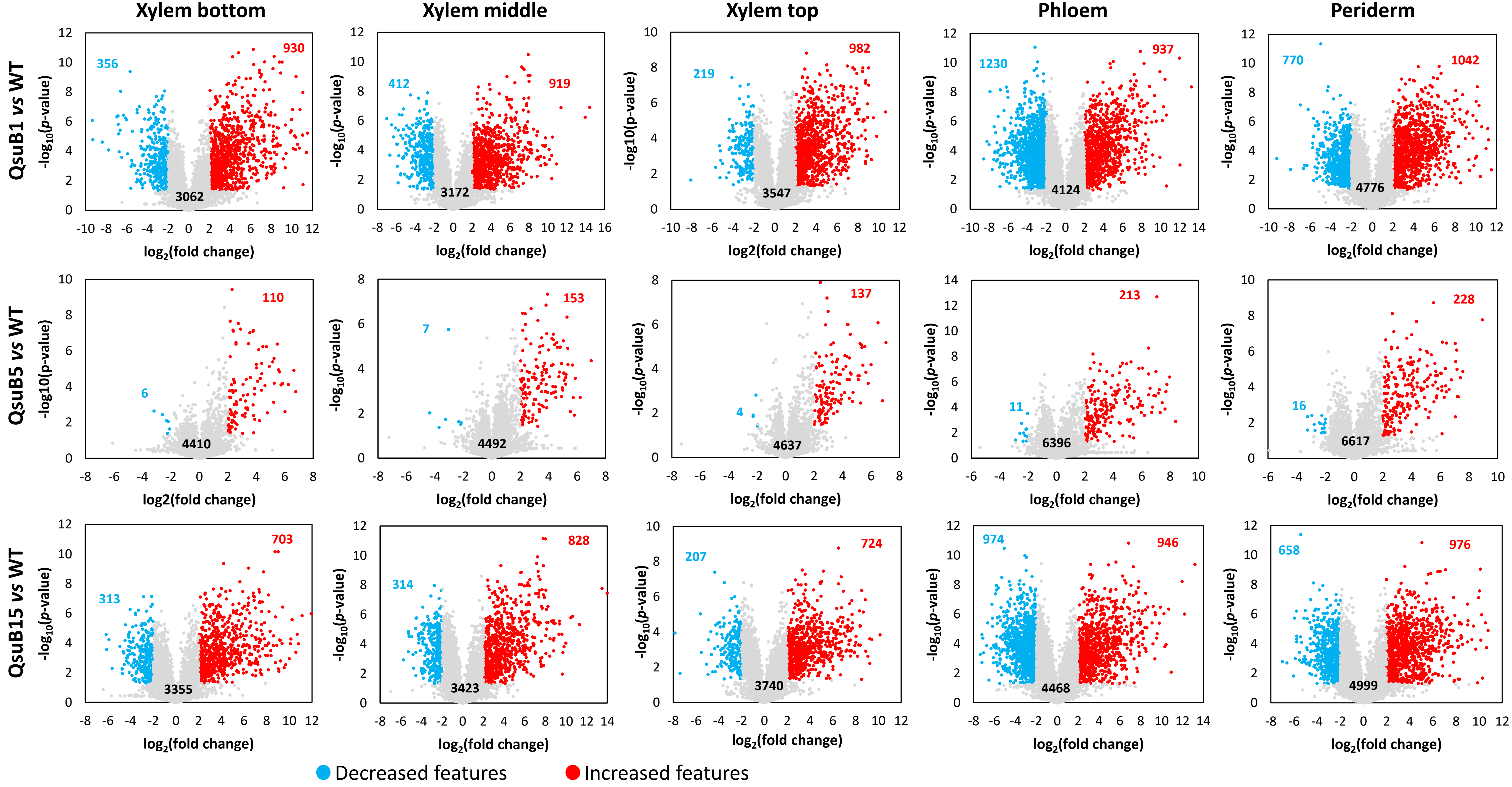

Dataset S8.7. Volcano plots of features detected in wildtype and QsuB transgenic lines in different stem tissues using C_{18} chromatography (negative ionization mode). Gray dots represent features not differentially abundant.

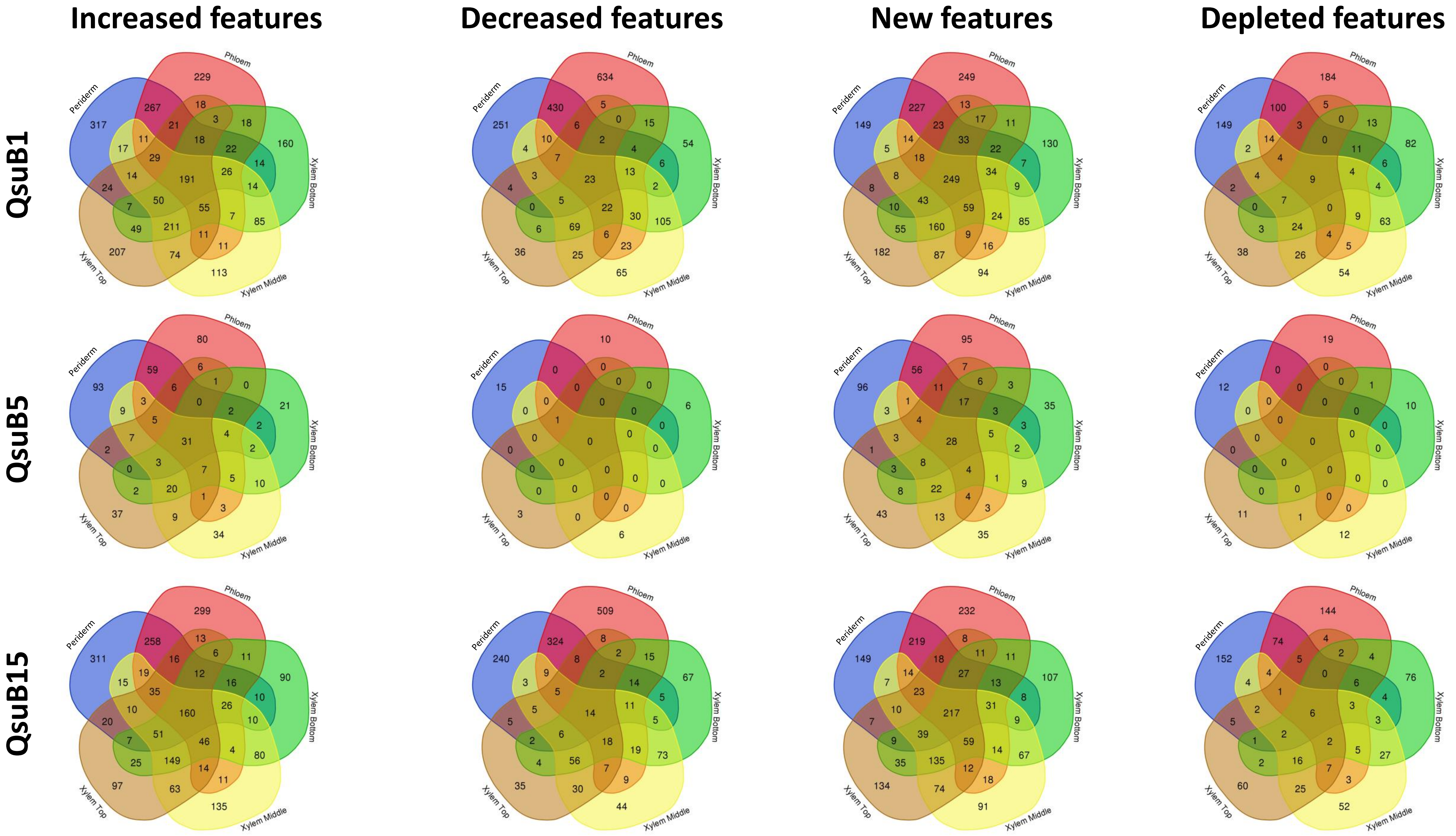

Dataset S8.8. Venn diagrams of features more abundant, less abundant, new, and depleted in different tissues of the QsuB lines (C_{18} negative ionization mode).
