# Supplementary material for: Expression of dehydroshikimate dehydratase in poplar induces transcriptional and metabolic changes in the phenylpropanoid pathway
Source: J Exp Bot. 2024 May 29;75(16):4960–77. doi: 10.1093/jxb/erae251 (PMC11349870; doi:10.1093/jxb/erae251)
Supplement: erae251_suppl_Supplementary_Materials [file erae251_suppl_supplementary_materials.zip › erae251_suppl_Supplementary_Figures_S1-S10.pdf]

Heterologous expression in poplar of dehydroshikimate dehydratase induces transcriptional and metabolic changes in phenylpropanoid pathway

Emine Akyuz Turumtay[1,2,3], Halbay Turumtay[1,2,4], Yang Tian[1,2], Chien-Yuan Lin[1,2], Yen Ning Chai[1,2], Katherine B. Louie[2,5], Yan Chen[1,6], Anna Lipzen[5], Thomas Harwood[2,5], Kavitha Satish Kumar[1,2], Benjamin P. Bowen[2,5], Qian Wang[7,8], Shawn D. Mansfield[7,8,9], Matthew J. Blow[5], Christopher J. Petzold[1,6], Trent R. Northen[2,5], Jenny C. Mortimer[1,2,10], Henrik V. Scheller[1,2,11], Aymerick Eudes[1,2,*]

[1] Feedstocks Division, Joint BioEnergy Institute, Emeryville, CA, USA
[2] Environmental Genomics and Systems Biology Division, Lawrence Berkeley National Laboratory, Berkeley, CA, USA
[3] Recep Tayyip Erdogan University, Department of Chemistry, 53100, Rize, Turkiye
[4] Karadeniz Technical University, Department of Energy System Engineering, 61830, Trabzon, Turkiye
[5] Joint Genome Institute, Lawrence Berkeley National Laboratory, Berkeley, CA, United States
[6] Biological Systems & Engineering Division, Lawrence Berkeley National Laboratory, Berkeley, CA, USA
[7] Department of Wood Science, University of British Columbia, Vancouver, BC, Canada
[8] Department of Botany, University of British Columbia, Vancouver, BC, Canada
[9] DOE Great Lakes Bioenergy Research Center, Wisconsin Energy Institute, Madison, WI 53726, USA
[10] School of Agriculture, Food and Wine & Waite Research Institute, University of Adelaide, Glen Osmond, SA, Australia.
[11] Department of Plant and Microbial Biology, University of California, Berkeley, Berkeley, CA, USA

*Correspondence: Aymerick Eudes, ageudes@lbl.gov

Supplementary Figures S1–S10

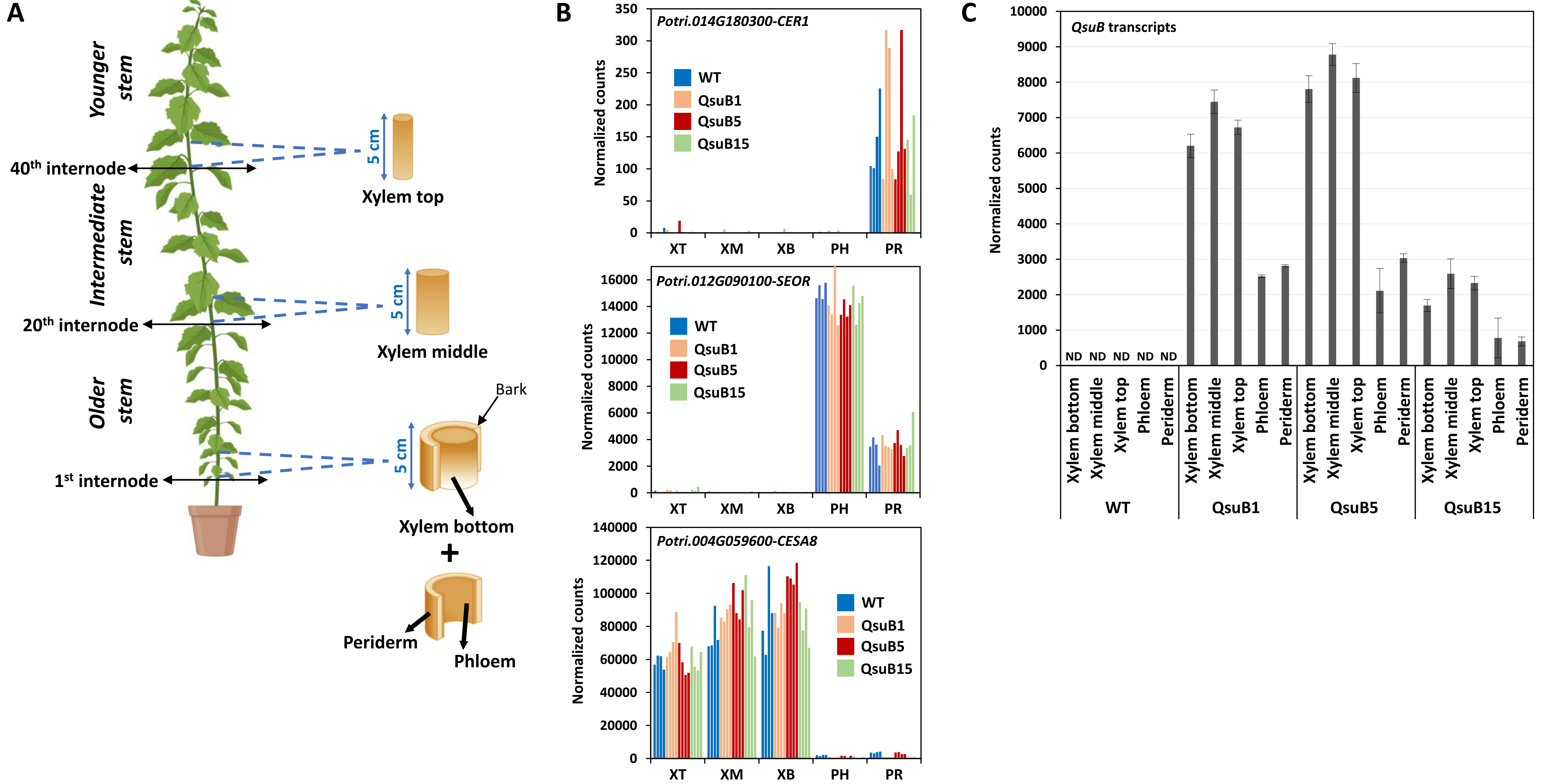

Figure S1. Stem tissue sampling approach used in this study. (**A**) Schematic diagram of tissue samples collected. Developing xylem tissues from the older, intermediate, and younger parts of the stem are referred to as xylem bottom, xylem middle, and xylem top, respectively. Phloem and periderm tissues were collected from the older part of the stem. Created with BioRender.com. (**B**) Expression analysis (DESeq2 normalized counts) of *PtCER1* (*Potri.014G180300*), *PtSEOR* (*Potri.012G090100*), and *PtCESA8* (*Potri.004G059600*) in each tissue types collected from WT and QsuB lines (four biological replicates each). XT, xylem top; XM, xylem middle, XB, xylem bottom, PH, phloem; PR, periderm. (**C**) *QsuB* transcript levels in the different tissue types from WT and QsuB lines. Values are means ±SE of four biological replicates. ND, not detected.

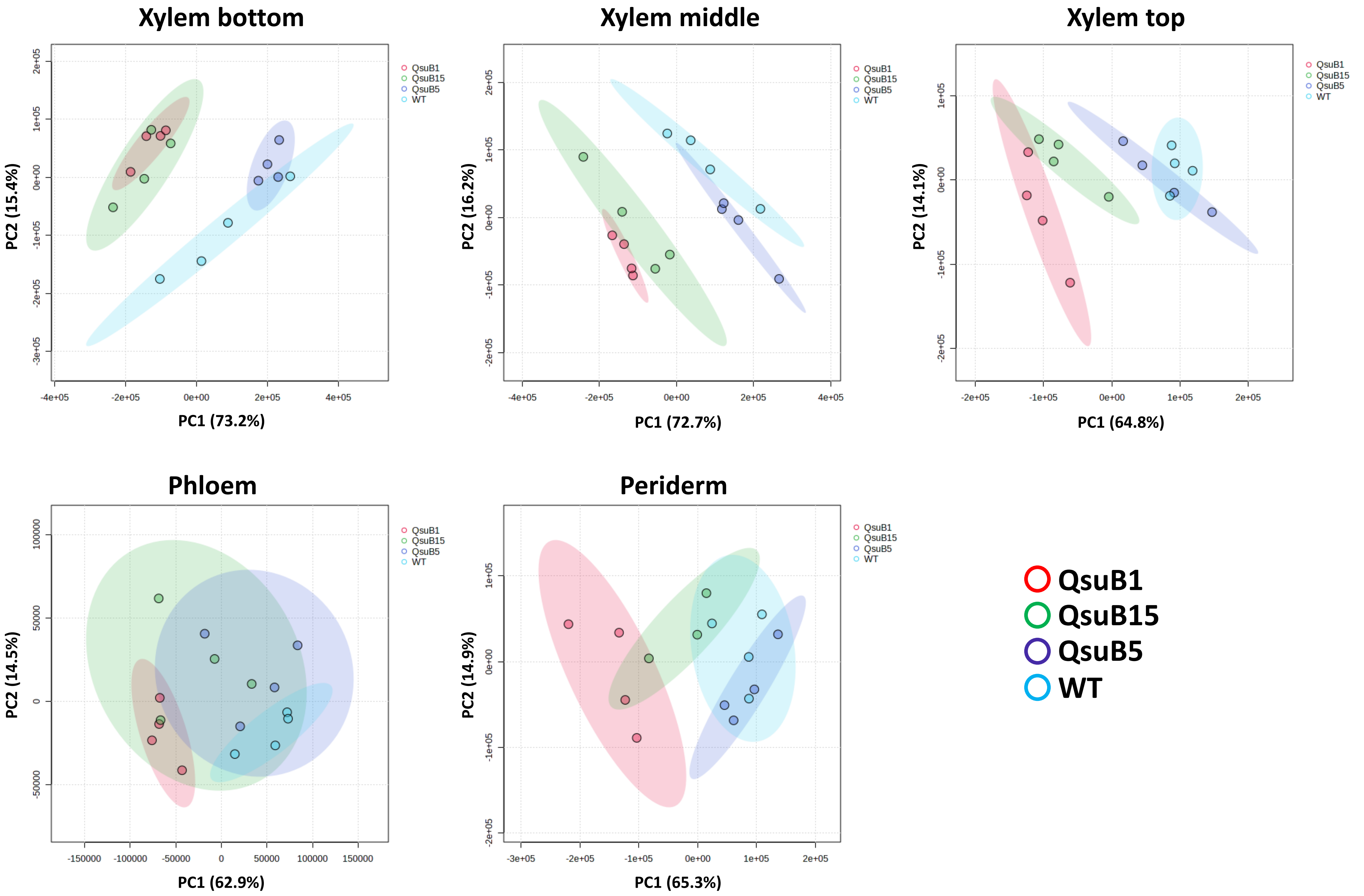

Figure S2. PCA plots of the transcripts identified in WT and QsuB lines in each tissue.

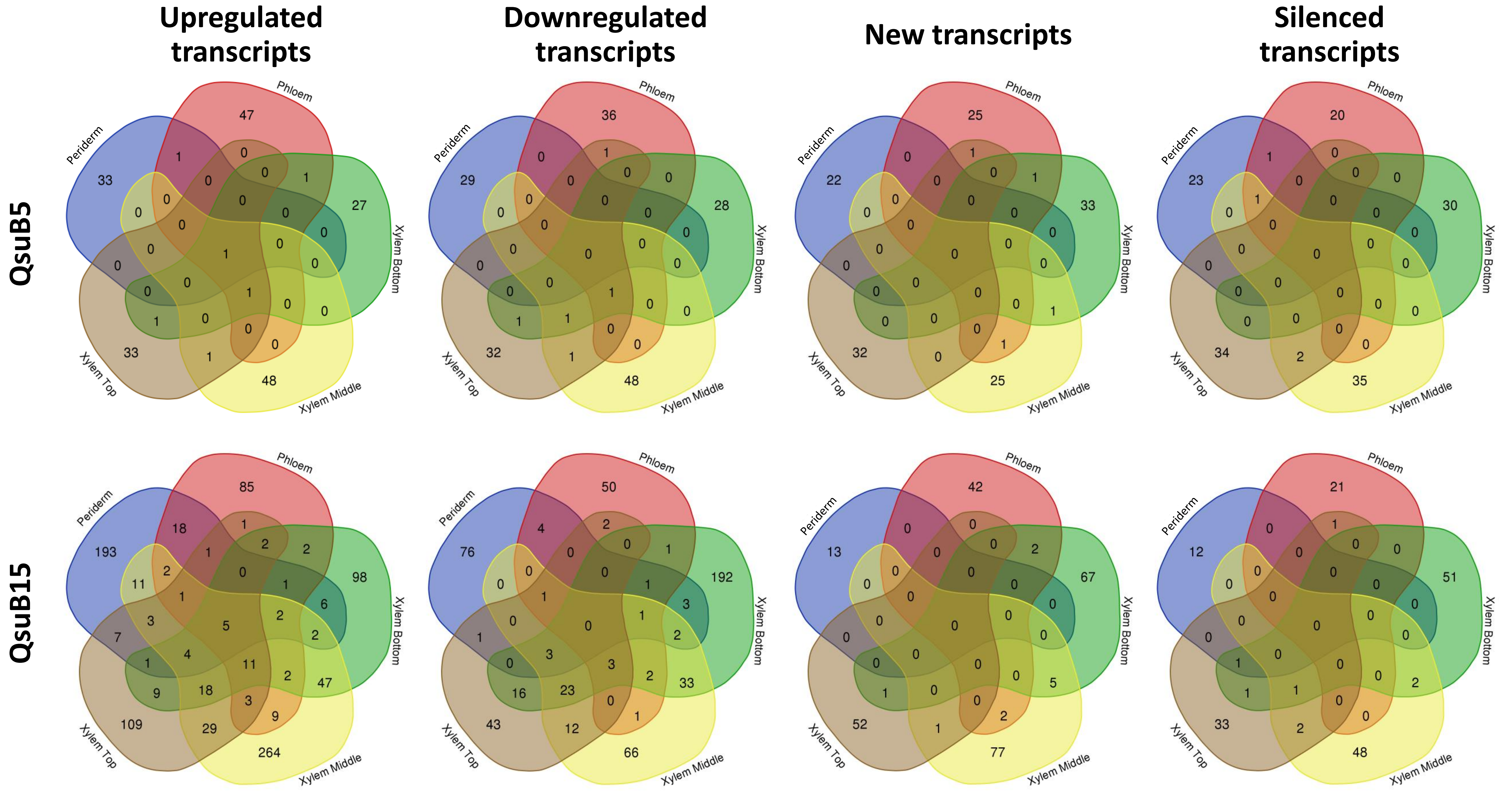

Figure S3. Venn diagrams of upregulated, downregulated, new, and silenced transcripts in different tissues of transgenic lines QsuB5 and QsuB15.

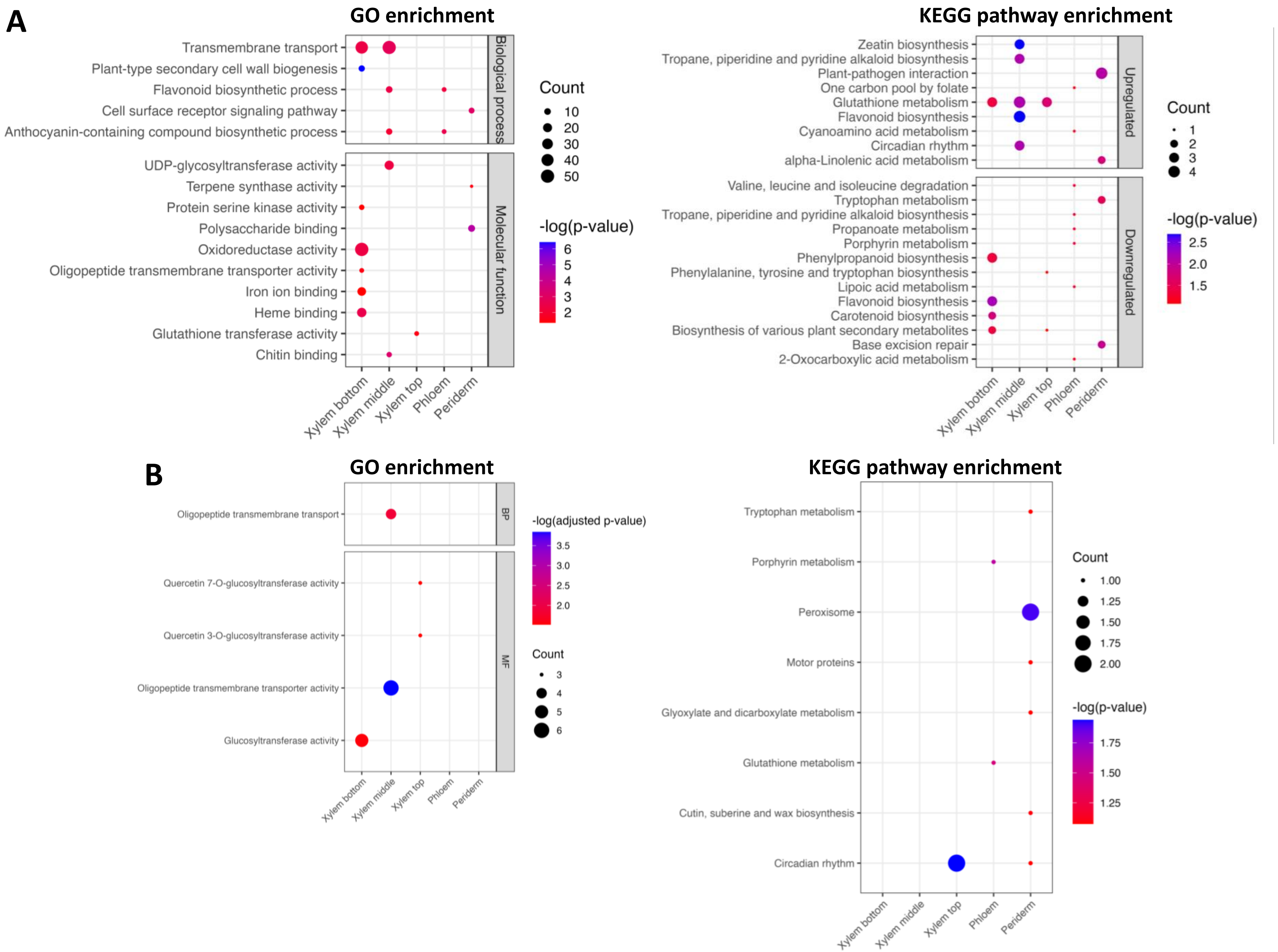

Figure S4. Dot plots of GO and KEGG enrichment analyses of DEGs identified in line QsuB15 (**A**) and QsuB5 (**B**). The size of the dots represents the number of genes associated with each ontology term and pathway.

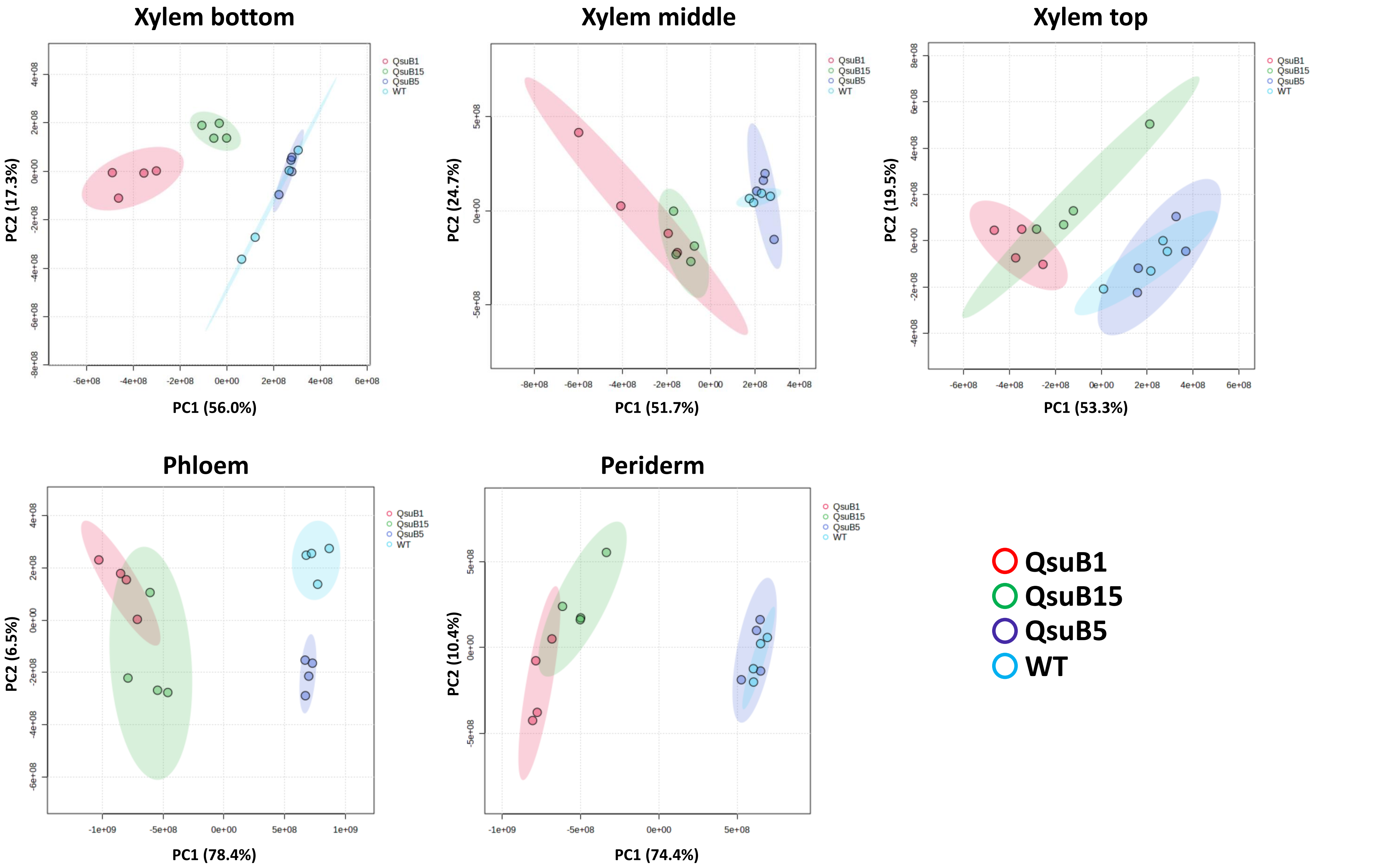

Figure S5. PCA plots of the features detected in WT and QsuB lines in each tissue (HILIC chromatography, positive ionization mode).

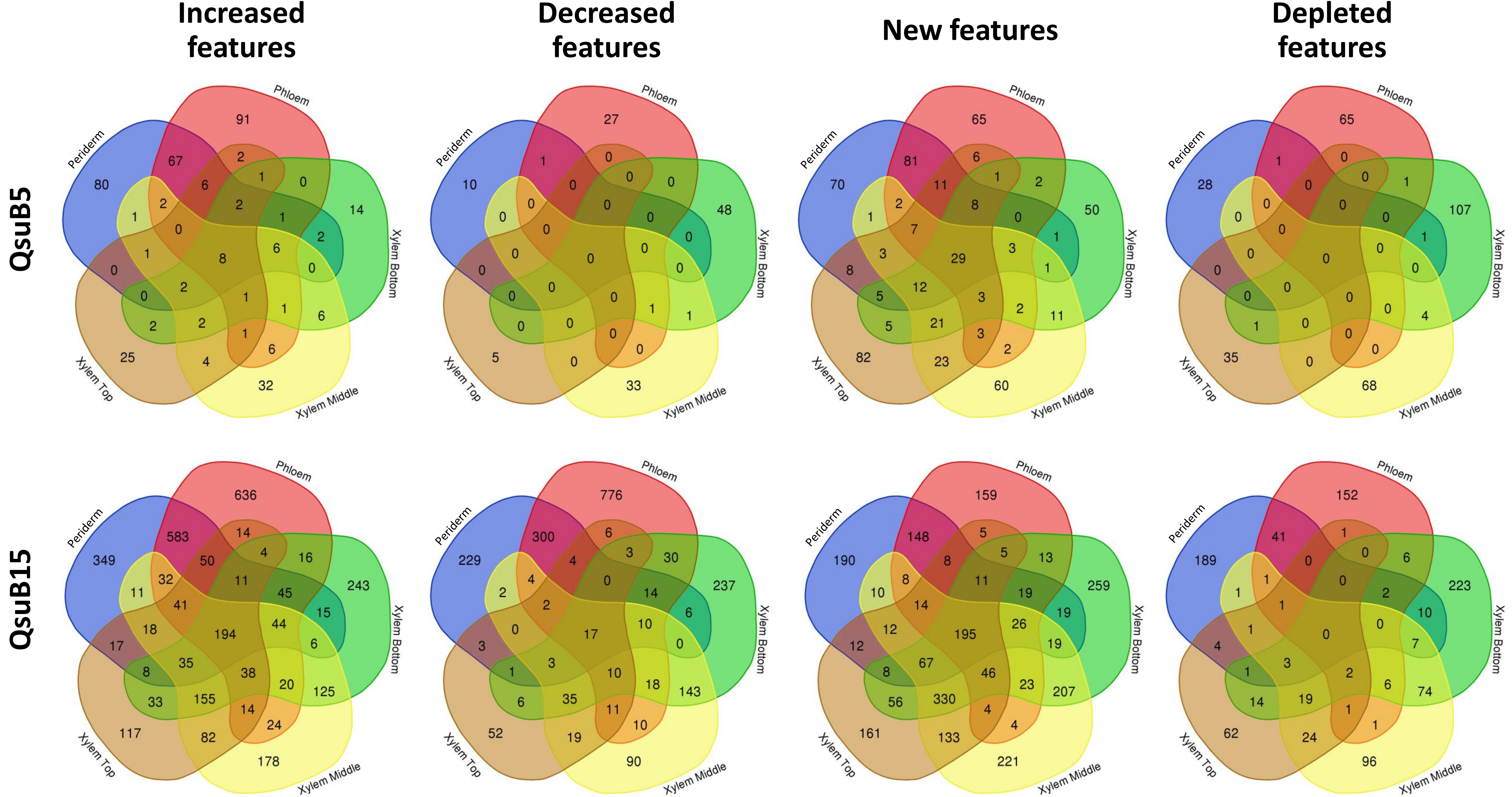

Figure S6. Venn diagrams of features more abundant, less abundant, new, and depleted in different tissues of lines QsuB5 and QsuB15 (HILIC positive ionization mode).

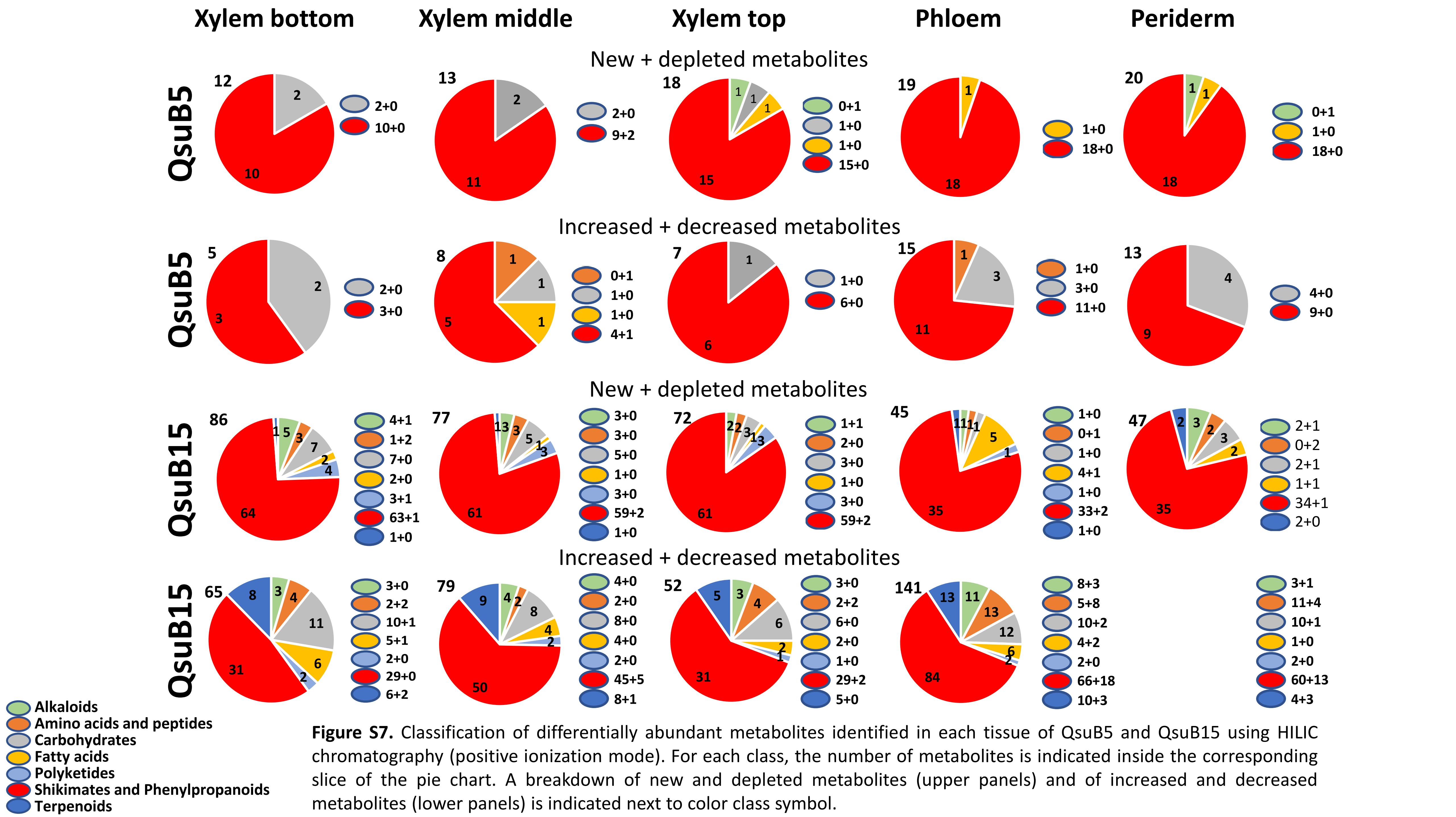

Figure S7. Classification of differentially abundant metabolites identified in each tissue of QsuB5 and QsuB15 using HILIC chromatography (positive ionization mode). For each class, the number of metabolites is indicated inside the corresponding slice of the pie chart. A breakdown of new and depleted metabolites (upper panels) and of increased and decreased metabolites (lower panels) is indicated next to color class symbol.

HILIC negative mode (10,962 unique features)

A

	WT	QsuB1	QsuB5	QsuB15
**Xylem bottom**	7,270	9,063	7,433	8,660
**Xylem middle**	7,497	9,211	7,563	9,001
**Xylem top**	7,667	9,399	8,207	9,022
**Phloem**	9,565	10,336	10,041	10,311
**Periderm**	9,590	10,256	10,014	10,226

B **Wildtype (10,424)**

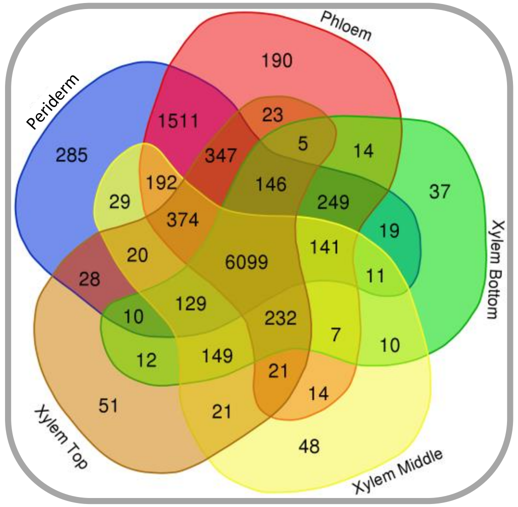

C **Xylem bottom (9,760)** **Xylem middle (9,848)** **Xylem top (9,919)** **Phloem (10,751)** **Periderm (10,680)**

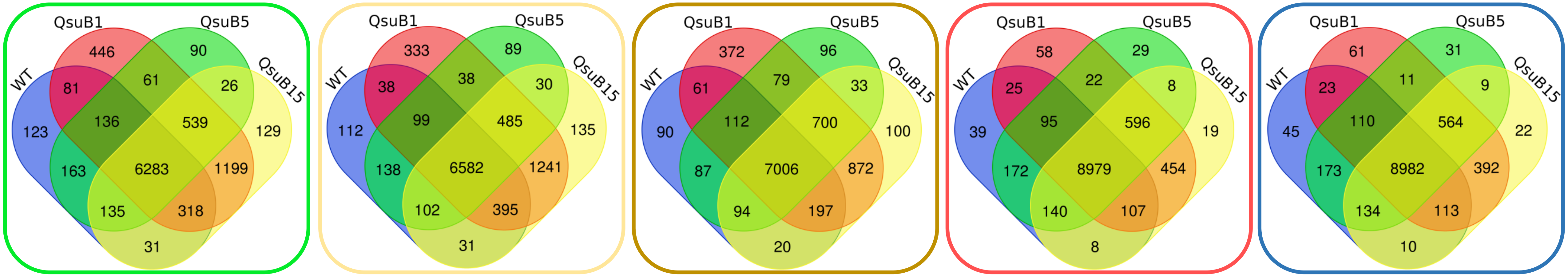

Figure S8. Features detected in WT and transgenic QsuB poplar lines using HILIC (negative ionization mode). Number of features detected in each tissue from the different lines (**A**). Venn diagram of features detected in different tissues from WT stems (**B**). Venn diagram of features detected in WT and QsuB lines for each tissue (**C**). The number of unique features is indicated in brackets for each tissue.

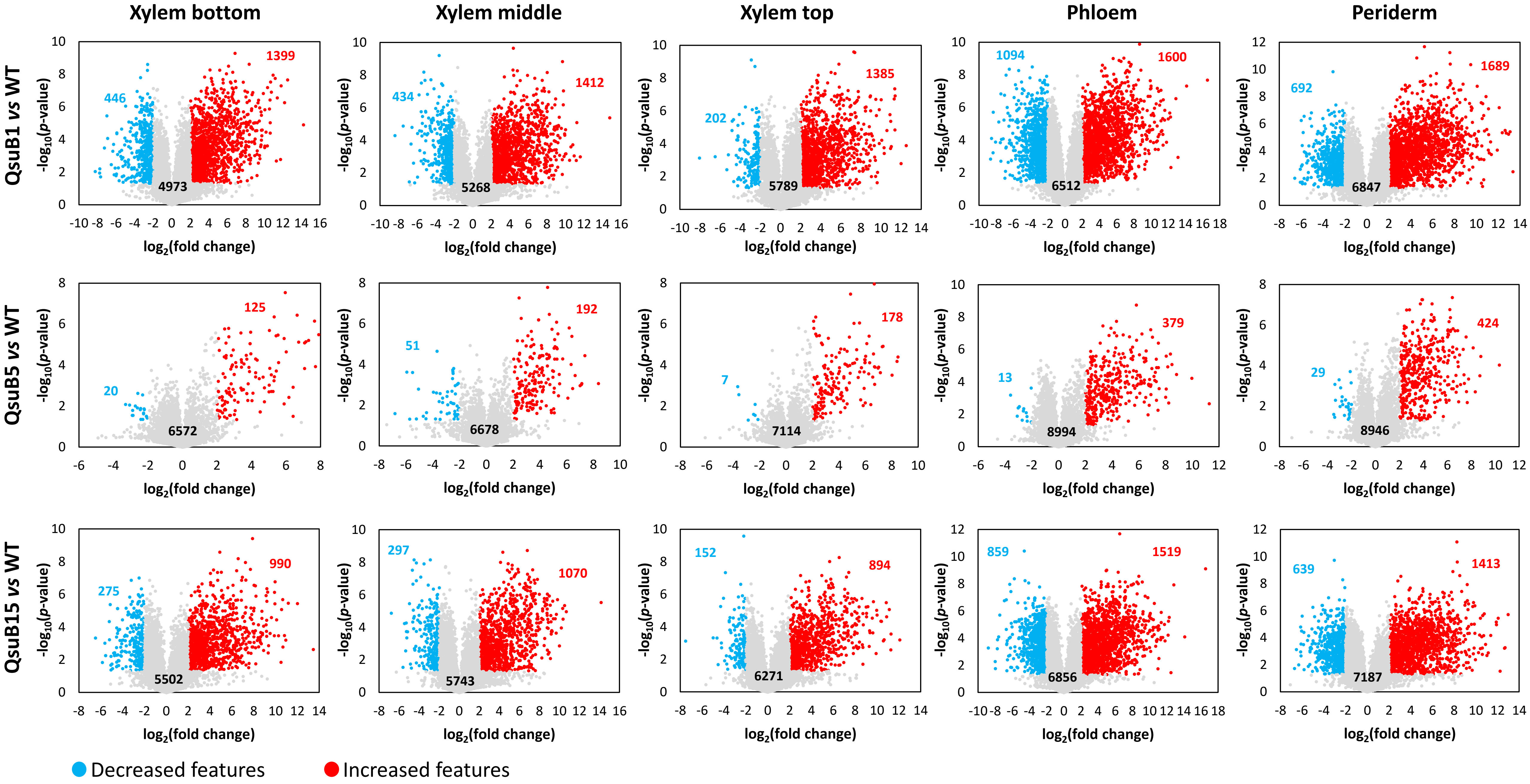

Figure S9. Volcano plots of features detected in WT and QsuB transgenic lines in different stem tissues using HILIC chromatography (negative ionization mode). Gray dots represent features not differentially expressed.

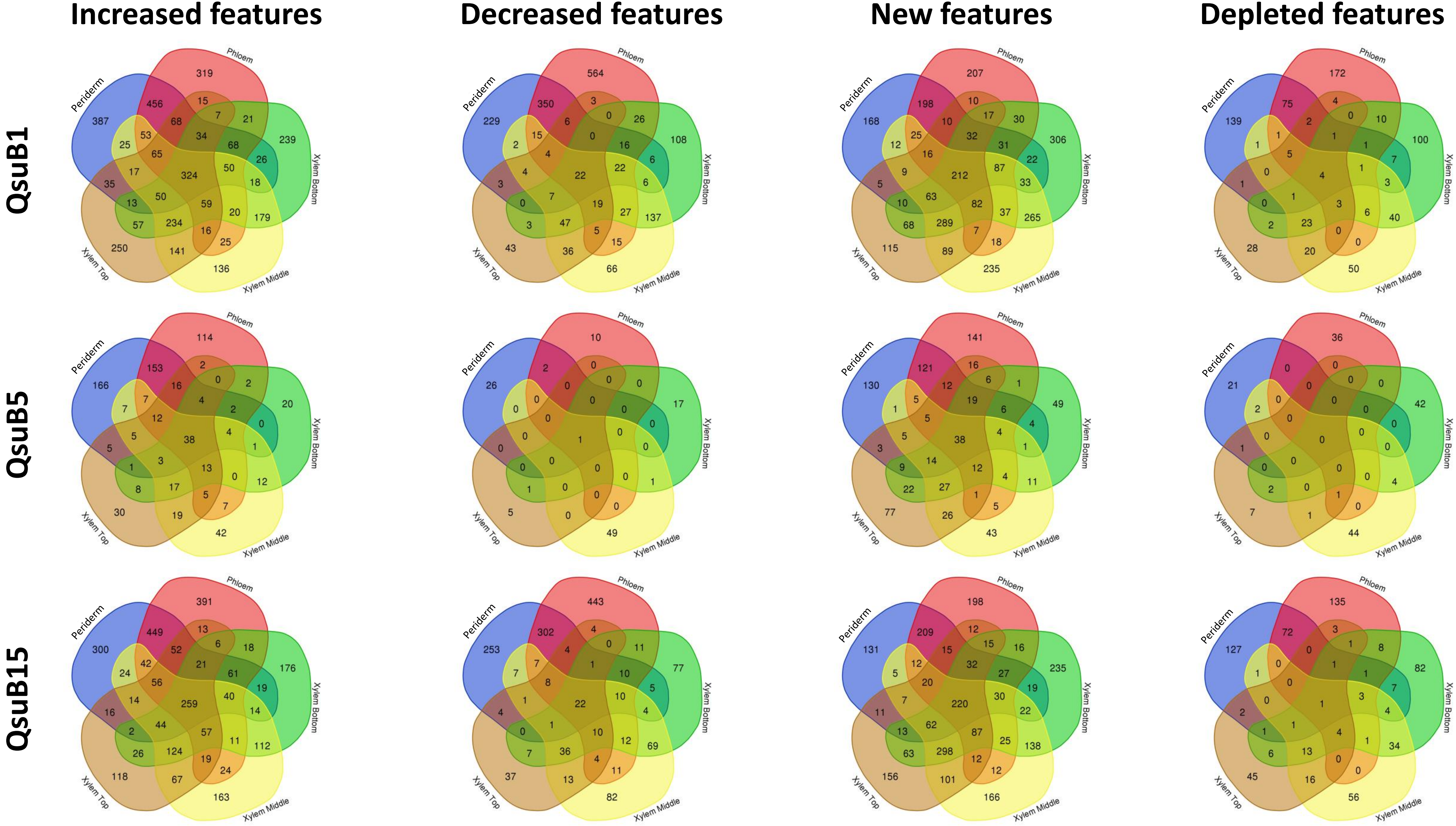

Figure S10. Venn diagrams of features more abundant, less abundant, new, and depleted in different tissues of the QsuB lines (HILIC negative ionization mode).
